# The deubiquitinating enzyme Cezanne stabilizes BRCA1 by counteracting APC/C and Ube2S-dependent Lys11-linked ubiquitination

**DOI:** 10.1371/journal.pbio.3003545

**Published:** 2025-12-08

**Authors:** Longqiang Wang, Xiao Wu, Atanu Paul, Jun Yao, Bin Wang

**Affiliations:** 1 Department of Genetics, The University of Texas MD Anderson Cancer Center, Houston, Texas, United States of America; 2 Genetics and Epigenetics Program, The University of Texas MD Anderson Cancer Center UT Health Houston Graduate School of Biomedical Sciences, Houston, Texas, United States of America; 3 Department of Molecular and Cellular Oncology, The University of Texas MD Anderson Cancer Center, Houston, Texas, United States of America; The Univ. of Texas at Austin, UNITED STATES OF AMERICA

## Abstract

The breast and ovarian tumor suppressor BRCA1 is a cell cycle-regulated protein and tumors with reduced BRCA1 protein level may share molecular features of *BRCA1*-mutant tumor and respond to PARPi therapy. Here, we identify that BRCA1 protein stability is controlled through ubiquitin lysine 11 (K11)-linkage modification under the regulation of Cezanne deubiquitinating enzyme, APC/C E3 ligase, and Ube2S E2 conjugating enzyme in a cell cycle-dependent manner. Cezanne-deficiency leads to increased BRCA1 K11-ubiquitination, decreased BRCA1 protein level, and increased cellular sensitivity to PARPi. The BRCA1 K11-linked ubiquitination is carried out through a degron on BRCA1 that is recognized by APC/C cofactor Cdh1. Tumor expression and mutational analyses indicate that Cezanne low or Ube2S high expression is associated with “BRCAness” and correlated with poor prognosis in breast cancer patients. Thus, our study has demonstrated a ubiquitin K11-linked ubiquitination pathway that regulates BRCA1 protein stability, dysregulation of which predicts BRCA1-deficiency that may be effectively targeted with PARPi therapy.

## Abstract 2

The levels of the breast and ovarian tumor suppressor BRCA1 fluctuate throughout the cell cycle and its depletion can induce tumors. This study identifies a new ubiquitin modification pathway that regulates BRCA1 protein stability during cell cycle, and shows that its dysregulation in tumors is associated with features of BRCA-mutated breast cancer.

## Introduction

BRCA1 plays critical roles in DNA homologous recombination repair (HR), cell cycle, and other cellular processes for the maintenance of genome stability. It has been indicated that BRCA1 protein level is regulated in a cell cycle-dependent manner via ubiquitination and proteasomal degradation of BRCA1 resulting in low-steady state level of BRCA1 in resting and early G1 and high level in S and G2 phase of the cell cycle [1,2]. Whereas an abundance of BRCA1 protein in S/G2 phase promotes HR, the low level of BRCA1 in G1 is necessary to prevent cells undergo illegitimate interhomolog or interchromosomal DNA recombination when sister chromatid is not available as a template [3]. Despite the findings that BRCA1 can be degraded by several ubiquitin and proteasome-mediated pathways involving E3 ligases, such as SCF^FBXO44^, HERC2, and HUWE1 [4–6], the mechanism by which BRCA1 protein levels is regulated in a cell cycle-dependent manner is still not clear.

Cezanne (also known as OTUD7B) is a member of the ovarian tumor (OTU) subfamily deubiquitinating enzyme (DUB) with a preference to cleave ubiquitin lysine 11 (K11)-linked ubiquitin conjugates [7–10]. Although it has also been reported that Cezanne possesses ability to disassemble K48 and K63-linked ubiquitin chains [11,12], recent studies indicate that the K11-linked DUB activity of Cezanne is crucial for DNA damage response and cell cycle regulation [13–15]. K11-linked polyubiquitination was first identified as a product of the Ube2S E2-conjugating enzyme [16]. In response to DNA double-strand breaks (DSBs), our previous work has shown that Cezanne antagonizes the activity of Ube2S and RNF8 E3 ligase in K11-linkage ubiquitin modification of chromatin bound proteins including H2A/H2AX on damaged chromatin [13]. We also have shown that Cezanne mediates the crosstalk between K63- and K11-linked ubiquitin signaling at DSBs through binding to K63-linked ubiquitin chains and disassembling K11-linked ubiquitin chains for the recruitment of K63-linked ubiquitin modification-dependent recruitment of DNA damage repair proteins, such as Abraxas/BRCA1-A complex, Rad18 and 53 BP1 [14]. During cell cycle progression, it has been shown that Cezanne acts as a cell cycle-regulated DUB counteracting the K11-linked ubiquitination catalyzed by Ube2S and the anaphase-promoting complex/cyclosome (APC/C) in the regulation of the degradation of APC/C substrates [15]. APC/C is a large multisubunit RING-finger E3 ligase complex which plays a major function during cell cycle progression targeting mitotic and G1 cell cycle-specific regulators for proteasomal degradation [17,18]. It has been shown that APC/C employs two E2 conjugating enzymes, Ube2S and Ube2C, to assemble K11-linked ubiquitin chain, in which Ube2C initiates and Ube2S elongates K11-linked polyubiquitin chain on APC/C substrates [19–23]. APC/C complex includes a catalytic core along with two additional co-activators, Cdc20 and Cdh1, which recruit substrates to the APC/C ligase complex during mitosis and G1 phases, respectively [17,18]. The APC/C substrates often possess a degron sequence that can be recognized by the activators, such as the D-box recognized by Cdc20 or the KEN-box recognized by Cdh1 [24–26]. It is not known whether BRCA1 protein level is subjected to the regulation of APC/C activity during cell cycle.

*BRCA1* is frequently mutated in familial breast and ovarian cancer. In sporadic tumors, while a *BRCA1* mutation is rare, reduced expression of BRCA1 through promoter methylation, somatic mutations and gene deletion have been observed in tumors without a *BRCA1* mutation [27–30]. Since the discovery of synthetic lethal interaction between inhibition of poly(ADP-ribose) polymerase (PARP) and *BRCA1* or *BRCA2* mutation [31,32], clinically approved PARP inhibitors (PARPi) have shown promising activity in the treatment of *BRCA1/2* mutated breast cancer [33,34]. Tumors that share molecular features of *BRCA*-mutant tumors—tumors with “BRCAness”—may also be effectively targeted with PARPi therapy [35,36]. Reduced BRCA1 protein levels might result in insufficient or loss of BRCA1 function that contribute to BRCA1 deficiency and tumors with reduced protein levels of BRCA1 may share molecular features of *BRCA1*-mutant tumor and respond to PARPi therapy.

In this study, we identify that BRCA1 undergoes K11-linked polyubiquitination and is subjected to the regulation of Cezanne, APC/C and Ube2S for ubiquitination and protein stability. Cezanne-deficiency leads to increased BRCA1 K11-ubiquitination, decreased BRCA1 protein level, and increased cellular sensitivity to PARPi. In addition, BRCA1 contains a KEN-box at its N-terminus and it interacts with APC/C coactivator Cdh1 through the KEN-box, indicating that BRCA1 is a substrate of APC/C. Cezanne antagonizes APC/C and Ube2S-dependent K11 ubiquitination of BRCA1, regulating BRCA1 protein level in the G1 and S phase of the cell cycle. Tumor expression and mutational analyses reveal that Cezanne low or Ube2S high expression in tumors is associated with BRCAness and correlated with poor prognosis in breast cancer patients. Furthermore, using a mouse xenograft model, we show that tumors with Cezanne-deficiency exhibited increased sensitivity to PARPi treatment. Thus, our study has demonstrated a ubiquitin K11-linked ubiquitination pathway regulating BRCA1 protein stability, in which Cezanne maintains BRCA1 protein stability through counteracting the activity of APC/C and Ube2S on BRCA1 ubiquitination and degradation. Dysregulation of this pathway due to reduced Cezanne or elevated expression of Ube2S predicts BRCA1-deficiency that may be effectively targeted with PARPi therapy.

## Results

### Cezanne regulates BRCA1 protein level through protein ubiquitination and degradation

**Fig 1:**
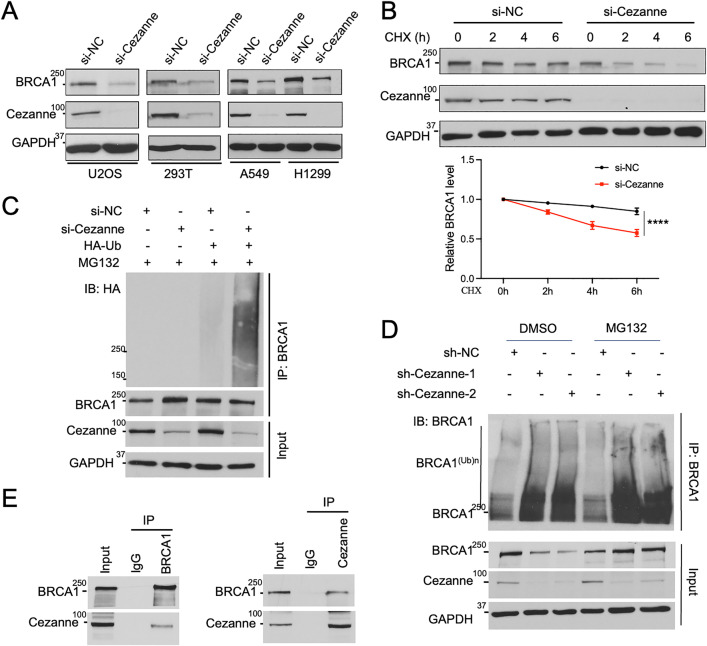
Cezanne regulates BRCA1 protein level through protein ubiquitination and degradation. **(A)** Knockdown of Cezanne reduces BRCA1 protein level in various cell lines. Cells were transfected with control (siNC) or Cezanne siRNAs (si-Cezanne-1). **(B)** Reduced BRCA1 protein stability in Cezanne knockdown cells. U2OS cells transfected with indicated siRNAs were treated with cycloheximide (CHX, 50 µg/ml). Relative BRCA level is measured by Image J and quantified from three independent experiments. Ordinary Two-way Anova was used for statistics, **** *p* < 0.0001. **(C)** Increased BRCA1 ubiquitination in Cezanne depleted cells. U2OS cells were transfected with indicated siRNAs in combination with or without HA-tagged ubiquitin (HA-Ub). Cells were treated with MG132 (20 µM, 6 h) before harvest. BRCA1 IP was performed under denaturing condition. **(D)** Increased ubiquitinated BRCA1 in the absence of Cezanne. BRCA1 IP was performed under denaturing condition with lysates of U2OS cells treated with indicated shRNAs. Ubiquitinated BRCA1 (BRCA^(Ub)n^) was detected as higher molecular weight “smears” in the western blot with BRCA1 antibody. **(E)** BRCA1 interacts with Cezanne. Immunoprecipitations were carried out with antibodies to BRCA1, Cezanne or normal mouse immunoglobulin G (IgG) (negative control) with U2OS cell lysates. GAPDH in **(A) (B) (C) (D)** was used as a loading control. Western blots shown are representative of three independent experiments. The data underlying the graph in the figure can be found in S1 Data.

We found that BRCA1 protein levels are significantly reduced in various cell lines treated with Cezanne siRNAs or shRNAs (Figs 1A, S1A, and S1B). The effect of Cezanne in regulating BRCA1 protein levels appears not to be influenced by DNA damage since the reduction of BRCA1 protein level occurred similarly when Cezanne siRNA-treated cells were treated or not treated with ionizing radiation (IR) (S1C Fig). Treatment with MG132, a proteasome inhibitor, restores the reduced BRCA1 protein amount in Cezanne siRNA-treated cells to the control level, suggesting that proteasome-mediated protein degradation may be involved (S1D Fig). By monitoring the stability of protein over time using cycloheximide chase assay, it is apparent that the stability of BRCA1 protein in Cezanne-deficient cells was significantly decreased (Fig 1B). We ruled out the possibility that the reduced BRCA1 abundance is due to a reduction of *Brca1* gene transcription. A comparison of *BRCA1* mRNA levels in the control or Cezanne depleted cells in multiple cell lines revealed that loss of Cezanne does not lead to a reduction of *BRCA1* mRNA levels (S1E Fig).

Since Cezanne is a DUB, we tested the possibility that Cezanne deubiquitinates BRCA1 and examined whether BRCA1 ubiquitination is affected when Cezanne is depleted. In cells transfected with HA-tagged ubiquitin (Ub), by performing BRCA1 immunoprecipitation under denaturing condition, we found that endogenous BRCA1 protein ubiquitination detected by HA antibody was significantly increased in Cezanne-depleted cells (**Fig 1C**). In addition to the smear of Ub-containing proteins bigger than BRCA1, there also appears smear that migrates faster than full-length BRCA1 (at 250 kDa), which could be due to antibody non-specificity or existence of BRCA1 isoforms or degraded BRCA1 fragments. To confirm that increased ubiquitinated form of BRCA1 (higher molecular weight smear) is seen in Cezanne-deficient cells, we also blotted the immunoprecipitated BRCA1 (under denaturing condition) with BRCA1 antibody. It shows that higher-molecular weight-smear of BRCA1 is indeed increased upon depletion of Cezanne (**Fig 1D**). These data indicate that BRCA1 ubiquitination is substantially increased in Cezanne-deficient cells. In addition, reciprocal co-immunoprecipitation experiment showed that endogenous BRCA1 interacts with Cezanne (**Fig 1E**). When we dissected the region on Cezanne that is responsible for the interaction with BRCA1 using Cezanne deletion mutants that we previously generated [14], it showed that the zinc-finger domain at the C-terminus of Cezanne is critical for the interaction (S1F Fig).

Thus, Cezanne likely interacts with BRCA1 as a DUB that inhibits BRCA1 ubiquitination.

### BRCA1 undergoes K11-linked ubiquitination that is regulated by Cezanne and Ube2S

**Fig 2:**
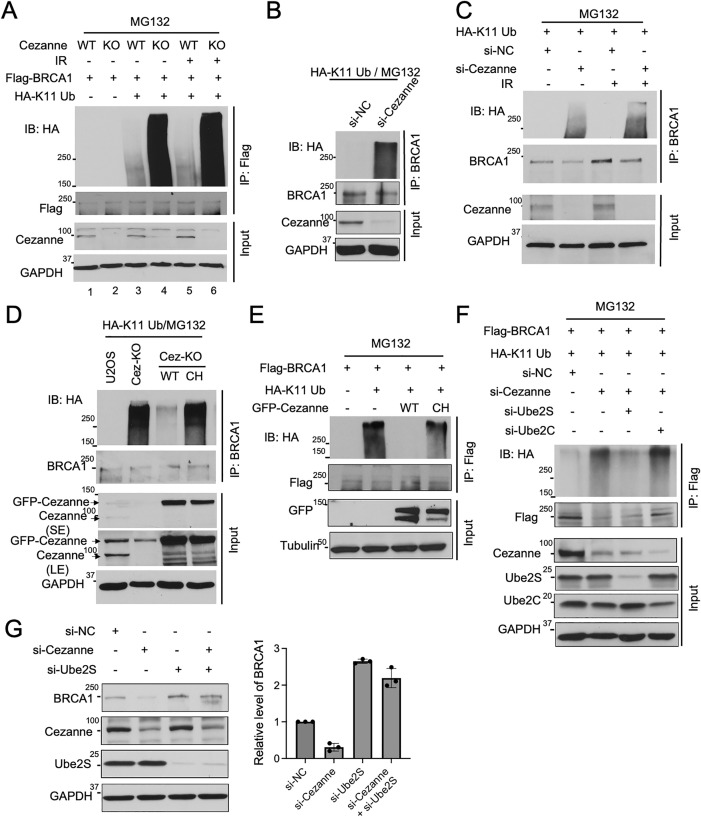
BRCA1 undergoes K11-linked ubiquitination that is regulated by Cezanne and Ube2S. **(A)** Increased K11-ubiquitination of BRCA1 in *Cezanne* KO cells untreated or treated with IR. Flag IP was performed under denaturing condition using lysates of WT or *Cezanne* KO U2OS cells expressing Flag-BRCA1 in combination with or without HA-K11 Ub. Cells were either untreated or treated with IR (10 Gy, 2 h). MG132 was added 6 h before harvest of cells. **(B)** Cezanne knockdown leads to increased endogenous BRCA1 K11-ubiquitination. BRCA1 IP was performed under denaturing condition. U2OS cells expressing HA-K11 Ub were treated with indicated siRNAs. MG132 was added 6 h before harvest of cells. **(C)** Increased endogenous BRCA1 K11-ubiquitination in Cezanne-depleted cells is independent of IR. U2OS cells were transfected with HA-K11-Ub and treated with indicated siRNA. Cells were also untreated or treated with IR (10 Gy, 2 **h)**. MG132 was added 6 h before harvest of cells. BRCA1 IP was performed under denaturing condition. **(D)** Cezanne DUB activity is required for K11 ubiquitination of BRCA1. U2OS WT or *Cezanne* KO (Cez-KO) cells, as well as Cez-KO cells complemented with expression of Cezanne WT or DUB-inactive CH mutant, were transfected with HA-K11 Ub. MG132 was added 6 h before harvest of cells. “SE”, short exposure; “LE” long exposure. BRCA1 IP was performed under denaturing condition. **(E)** Overexpression of Cezanne decreases BRCA1 K11-ubiquitination. U2OS cells were transfected with Flag-BRCA1 and with or without HA-K11-Ub, GFP-tagged wild-type (WT) or CH mutant of Cezanne. MG132 was added 6 h before harvest of cells. Flag IP was performed under denaturing condition. **(F)** Depletion of Ube2S reduces the increased K11-ubiquitination of BRCA1 in Cezanne-deficient cells. U2OS cells were transfected with HA-K11-Ub and Flag-BRCA1, followed by transfection with indicated siRNAs. MG132 was added 6 h before harvest of cells. Flag IP was performed under denaturing condition. **(G)** Depletion of Ube2S restores BRCA1 protein level in Cezanne-siRNA-treated cells. Total lysates of cells treated with indicated siRNAs were examined by western blots. Relative amount of BRCA1 is measured by Image J and quantified from three independent experiments. GAPDH in **(A) (B) (C) (D) (F) (G)** and tubulin in **(E)** was used as a loading control. Western blots shown are representative of three independent experiments. The data underlying the graph shown in the figure can be found in S1 Data.

Since Cezanne is identified as a K11-linkage-specific DUB [10], we tested whether BRCA1 undergoes K11-linked ubiquitination. We found that exogenously expressed Flag-tagged BRCA1 could be conjugated by a K11-linkage-specific ubiquitin mutant (K11 Ub, a ubiquitin mutant that only contains lysine 11 residue intact) (**Fig 2A**, lane 3, 5) and depletion of Cezanne by knockout (KO) greatly enhanced BRCA1 K11 Ub conjugation (**Fig 2A**, lane 4, 6). We also compared BRCA1 K11- with K48- or K63-linkage-specific ubiquitination using K48- or K63-ubiquitin mutant (which only contains lysine 48 or 63 residue intact, respectively). Whereas Cezanne depletion leads to increased BRCA1 K11 ubiquitination, it does not appear to affect K48- or K63-ubiquitin conjugation of endogenous BRCA1 (S2A Fig). Endogenous BRCA1 K11 ubiquitination is also substantially increased when Cezanne is depleted (Fig 2B). In addition, the effect of Cezanne on BRCA1 K11 ubiquitination is independent of IR (Figs 2A, 2C, and S2B). The elevated BRCA1 K11 ubiquitination upon loss of Cezanne is repeatedly observed in various cell lines, supporting the critical role of Cezanne in counteracting K11 ubiquitination of BRCA1 (S2C Fig). Thus, the effect of loss of Cezanne on BRCA1 ubiquitination is specific for K11-linkage ubiquitination. We then examined whether the DUB activity of Cezanne is required for BRCA1 K11 ubiquitination. By complementing *Cezanne* KO cells with expression of WT or a DUB inactive mutant of Cezanne (CH), we found that the elevated K11 ubiquitination of BRCA1 in *Cezanne* KO cells is reduced by expression of WT but not CH mutant of Cezanne, supporting that Cezanne reduces K11 ubiquitination of BRCA1 through its DUB activity (**Fig 2D**). In addition, overexpression of GFP-tagged Cezanne WT, but not CH mutant, largely reduced the K11-ubiquitination of BRCA1 in cells (**Fig 2E**). Thus, the DUB activity of Cezanne is critical in regulating K11-linked ubiquitination of BRCA1. To confirm that K11-ubiquitin modified BRCA1 is a substrate of Cezanne DUB activity, we purified Flag-BRCA1 under denaturing condition from cells expressing Flag-BRCA1 and myc-K11 Ub and performed an in vitro DUB assay with HA-tagged Cezanne WT or CH mutant immunoprecipitated from cells. It shows that Cezanne WT but not CH mutant was able to cleave the K11-Ub modified BRCA1 (S2D and S2E Fig). Together, these results indicate that BRCA1 undergoes K11-linked ubiquitin modification and Cezanne disassembles K11-linked ubiquitin conjugates from BRCA1.

We then examined the effect of Ube2S, a K11-linkage-specific ubiquitin conjugating enzyme [19,22,23], on the level of BRCA1 protein. If K11-ubiquitination of BRCA1 depends on Ube2S, depletion of Ube2S should reduce the overly increased ubiquitin conjugation on BRCA1 due to Cezanne-deficiency. We found that this is indeed the case. When Ube2S was knocked down in Cezanne siRNA-treated cells, the elevated K11 ubiquitination of BRCA1 was decreased to the control level (**Fig 2F**). Knocking down Ube2S in Cezanne-depleted cells also restored the levels of BRCA1 protein (**Fig 2G**). Thus, the K11-ubiquitin conjugation of BRCA1 is likely carried out by Ube2S and antagonized by Cezanne. Ube2C has been shown functioning together with Ube2S in APC/C mediated K11-conjugation of substrate proteins. We found that knocking down Ube2C had minimal effect on the elevated K11 ubiquitin conjugation of BRCA1 in Cezanne-depleted cells (**Fig 2F**). Knocking down Ube2S alone mildly increased the levels of BRCA1, consistent with Ube2S’ role in K11-ubiquitination mediated protein degradation of BRCA1 (S2F Fig). Together, these data indicate that BRCA1 undergoes K11 ubiquitination that is regulated by a deubiquitinating enzyme Cezanne and an E2 conjugating enzyme Ube2S.

### BRCA1 K11 ubiquitination is regulated by APC/C^Cdh1^

**Fig 3:**
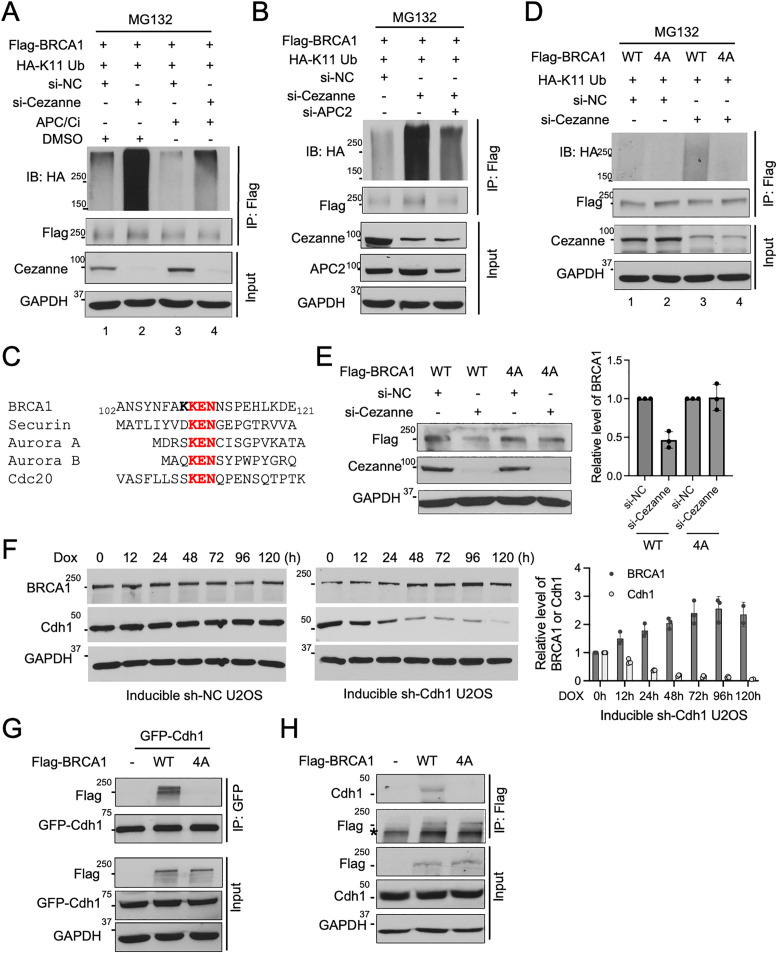
BRCA1 K11 ubiquitination is regulated by APC/C^Cdh1^. **(A)** APC/C inhibitor (APC/Ci) treatment reduces the elevated BRCA1 K11-ubiquitination due to Cezanne deficiency. U2OS cells expressing Flag-BRCA1 and HA-K11 Ub were transfected with indicated siRNA and treated with DMSO or APC/C inhibitor proTAME. Cells were treated with MG132 (20 µM, 6 h) before harvest. FLAG IP was performed under denaturing condition. **(B)** Knockdown of APC2 reduces the elevated BRCA1 K11 ubiquitination in Cezanne-deficient cells. U2OS cells transfected with Flag-BRCA1 and HA-K11-ub were transfected with indicated siRNAs. Cells were treated with MG132 (20 µM, 6 h) before harvest. FLAG IP was performed under denaturing condition. **(C)** BRCA1 possesses KEN box at its N-terminus. Alignment of KEN box of BRCA1 and several KEN box-containing proteins. **(D)** Mutation of KKEN abolishes BRCA1 K11 ubiquitination. U2OS cells were transfected with HA-K11-Ub and Flag-BRCA1 WT or 4A (KKEN/AAAA) mutant followed by transfection with control or Cezanne siRNAs. Cells were treated with MG132 (20 µM, 6 h) before harvest. FLAG IP was performed under denaturing condition. **(E)** BRCA1 KKEN mutant is resistant to Cezanne regulation. U2OS cells expressing Flag-BRCA1 WT or KKEN (4A) mutant were transfected with control or Cezanne siRNAs. Relative amount of BRCA1 is measured by Image J and quantified from three independent experiments. **(F)** Inducible knockdown of Cdh1 leads to increased BRCA1 protein level. U2OS cells with inducible expression of control or Cdh1 shRNA upon doxycycline (Dox, 2 µg/ml) at indicated times were analyzed. Relative amount of BRCA1 and Cdh1 protein for Inducible sh-Cdh1 cells were measured with Image J and quantified from three independent experiments. **(G)** GFP-Cdh1 interacts with Flag-BRCA1. 293T cells were transfected with GFP-Cdh1 and Flag-BRCA1 WT or 4A mutant. **(H)** Flag-BRCA1 interacts with endogenous Cdh1. 293T cells were transfected with Flag-BRCA1 WT or 4A mutant. GAPDH in **(A) (B) (D) (E) (F) (G) (H)** was used as a loading control. Western blots shown are representative of three independent experiments. The data underlying the graphs shown in the figure can be found in S1 Data.

It has been shown that Ube2S functions with APC/C catalyzing K11 ubiquitination of cell cycle regulators [19–23]. In addition, our earlier studies indicate that Ube2S functions with RNF8 in catalyzing K11-linked ubiquitin modification on damaged chromatin [13]. We ruled out RNF8 as the E3 ligase for BRCA1 ubiquitination since knockdown of RNF8 did not lead to increased BRCA1 protein level, nor did it restore BRCA1 levels in Cezanne-deficient cells (S3A and S3B Fig). Instead, we found that inhibition of APC/C by proTAME, an APC/C inhibitor, appeared to restore the decreased BRCA1 protein level in Cezanne-depleted cells (S3C Fig). Indeed, the elevated BRCA1 K11-ubiquitination in Cezanne siRNA-treated cells was much reduced upon treatment with proTAME (Fig 3A, compare lane 2 and 4). Knockdown of Apc2, a core component of the APC/C E3 ligase complex also reduced the elevated K11 ubiquitination of BRCA1 in Cezanne-depleted cells (Fig 3B). Like the effect of Ube2S knockdown, APC/C inactivation by proTAME or Apc2 depletion mildly increased BRCA1 protein levels (S3D and S3E Fig). Thus, APC/C activity is involved in the regulation of BRCA1 K11-ubiquitination and protein levels.

APC/C becomes activated upon sequential binding to the CDC20 and Cdh1 adaptor proteins [17,18]. We found a putative KEN box at the N-terminus of BRCA1 protein (Fig 3C), suggesting that BRCA1 may be regulated through Cdh1. Since the lysine residue in front of the KEN box is also conserved in several mammalian species (S3F Fig), we constructed a KKEN mutant (4A, all four residues mutated to alanine) and compared the K11-ubiquitin conjugation of BRCA1 WT and 4A mutant. The elevated BRCA1 K11-ubiquitination caused by Cezanne knockdown only occurs to WT (Fig 3D, lane 3) but not to the 4A mutant of BRCA1 (Fig 3D, lane 4), indicating that Cezanne counteracts the KKEN sequence-dependent K11 ubiquitination of BRCA1. In addition, when we compared the effect of depletion of Cezanne on exogenously expressed BRCA1 WT or 4A mutant, it appears that while Flag-tagged BRCA1 WT was decreased upon Cezanne knockdown, the 4A mutant protein level was less affected by Cezanne depletion (Fig 3E).

To determine whether Cdh1 mediates the degradation of BRCA1, we examined BRCA1 protein levels upon knockdown of Cdh1. Considering Cdh1 is a key regulator in cell cycle regulation targeting many substrates for degradation, we generated an inducible system to knock down Cdh1 using doxycycline (Dox) induced-shRNA expression. Upon induction of Cdh1 shRNAs, as Cdh1 protein level decreased, BRCA1 protein level showed a significant increase, demonstrating a role of Cdh1 in regulating BRCA1 protein levels (Fig 3F). In addition, co-immunoprecipitation experiments showed that GFP-Cdh1 interacts with WT but not 4A mutant of BRCA1 (Fig 3G) and that Flag-BRCA1 WT but not 4A mutant interacts with endogenous Cdh1 (Fig 3H). Thus, Cdh1 interacts with BRCA1 and mediates the K11 ubiquitination of BRCA1. BRCA1 heterodimerizes through the N-terminal RING domain (20–68 aa) with BARD1 [37]. The BRCA1 4A mutant retains the ability to heterodimerize with BARD1, suggesting that the BRCA1-BARD1 heterodimer forms independently of BRCA1’s binding to Cdh1 (S3G Fig). Consistently, an established cancer derived BARD1-binding-deficient-BRCA1 C61G mutant [38] still interacts with Cdh1 (S3H Fig). Together, these results indicate that BRCA1 ubiquitination is dependent on APC/C through Cdh1 recognition of the KEN box presented on BRCA1.

### Cezanne regulates BRCA1 protein level at G1 and S but not G2 phase of the cell cycle

**Fig 4:**
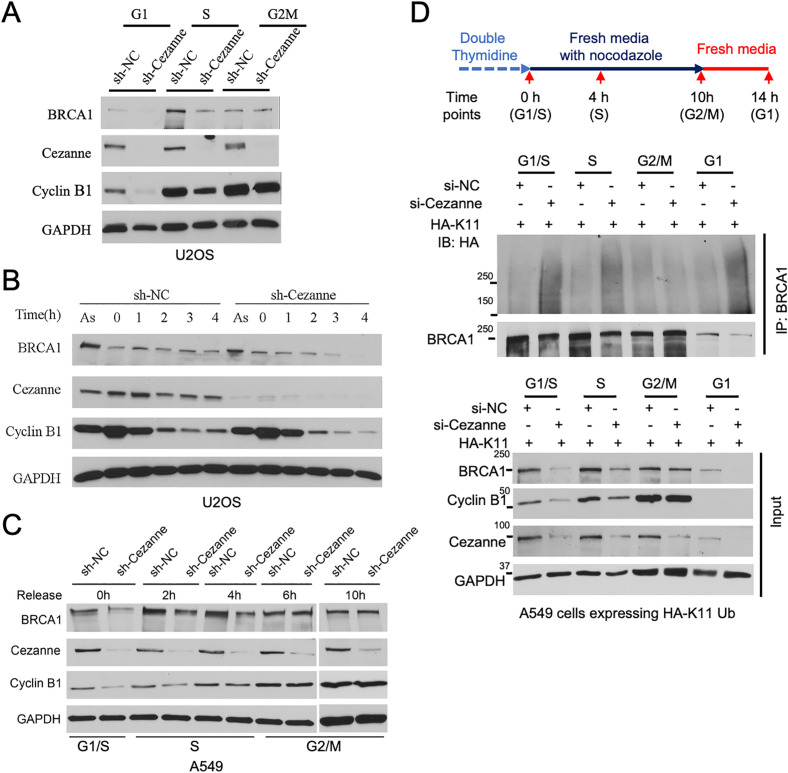
Regulation of BRCA1 by Cezanne is cell-cycle dependent. **(A)** Cezanne knockdown leads to decreased BRCA1 protein level in cells synchronized in G1 and S phase of the cell cycle. Cell cycle distribution for indicated samples is shown in S4A Fig. **(B)** BRCA1 protein level reduction in Cezanne-deficient cells during mitotic exit to the G1 phase of cell cycle. Samples from asynchronous (As) cells and cells released from nocodazole arrest (G2/M) at indicated times are shown. Cell cycle distribution for indicated samples is shown in S4B Fig. **(C)** Reduced BRCA1 protein level in G1/S and S phase Cezanne-deficient cells. A549 Cells were synchronized at G1/S with double thymidine block and released into medium containing nocodazole at indicated times. Cell synchronization is shown in S4E Fig. **(D)** Cezanne regulates BRCA1 K11-ubiquitination and protein level in G1, S, but not G2/M phase of the cell cycle. A549 cells expressing HA-K11 Ub and transfected with indicated siRNAs were synchronized following the illustrated scheme. BRCA1 IP was performed under denaturing condition. Cell cycle distribution for indicated samples is shown in S5A Fig. GAPDH in **(A) (B) (C) (D)** was used as a loading control. Western blots shown are representative of three independent experiments.

APC/C-Cdh1 (APC/C^Cdh1^) plays a critical role in the cell exit of mitosis and throughout G1 phase [17,18,39]. It has been shown that Cezanne antagonizes APC/C activity in the K11 ubiquitination and degradation of several established APC/C substrates [15]. We examined whether the regulation of BRCA1 protein level by Cezanne is cell cycle dependent. By synchronizing cells and monitoring BRCA1 protein level when majority of the cells are in G1, S or G2/M phase of the cell cycle, it appears that Cezanne deficiency-induced BRCA1 protein level reduction mainly occurs in G1 and S but not G2 phase (Figs 4A and S4A). Cyclin B1, a known substrate of APC/C^Cdh1^, which was used as a control, showed a similar pattern. To further analyze it, we first examined the regulation of BRCA1 by Cezanne during mitotic exit and in G1 phase by synchronizing cells at prometaphase with nocodazole and monitoring BRCA1 protein level overtime after cells were released into fresh media. Upon mitotic exit, in two different Cezanne-deficient cells, BRCA1 protein abundance showed accelerated reduction as cells progress into G1 phase (Figs 4B and S4B–S4D). By synchronizing cells at G1/S with double-thymidine block and releasing them into fresh media containing nocodazole, we monitored the effect of Cezanne loss on BRCA1 levels when cells progress from G1/S to S and G2 phase. Loss of Cezanne led to reduced BRCA1 levels at G1/S (0 h) and when cells are progressing through S phase (2 and 4 h). Once cells progress into G2 (6 h) and arrest at G2/M phase with nocodazole (10 h), the effect of Cezanne loss on BRCA1 levels disappeared (Figs 4C and S4E). Similar results were seen with a different cell line (S4F and S4G Fig). These results support that the effect of Cezanne on regulating BRCA1 protein levels is mainly in G1 and S phase of the cell cycle.

We then monitored BRCA1 ubiquitination throughout cell cycle by following the synchronization strategy shown in Fig 4D. Cells were synchronized with double thymidine block at G1/S, released into fresh media containing nocodazole until cells were synchronized at G2/M, followed by releasing into fresh media. Cells transfected with linkage-specific HA-tagged K11- or K48 Ub and treated with either control or Cezanne siRNAs were collected at indicated cell cycle stages (S5A and S5B Fig). Corresponding to the reduction of BRCA1 protein level in Cezanne-deficient cells at G1/S, S, and the next G1 phase after arrest, BRCA1 K11-ubiquitination levels in Cezanne-deficient cells were robustly increased when compared to that of control siRNA-treated cells (Fig 4D). At G2/M, however, not much difference was seen between the control and Cezanne-depleted cells in the amount of K11 ubiquitination (Fig 4D) or BRCA1 protein levels (Fig 4D, input). These data indicate that BRCA1 K11-ubiquitination is robustly inhibited by Cezanne during G1 and S but not G2/M phase. In comparison, Cezanne depletion does not affect the K48-ubiquitination of BRCA1 throughout the cell cycle (S5B and S5C Fig).

Together, these data indicate that Cezanne-dependent regulation of BRCA1 controls BRCA1 protein levels at G1, S, but not G2 phase of the cell cycle.

### Cezanne downregulation is linked with “BRCAness” in tumors and correlated with poor prognosis

**Fig 5:**
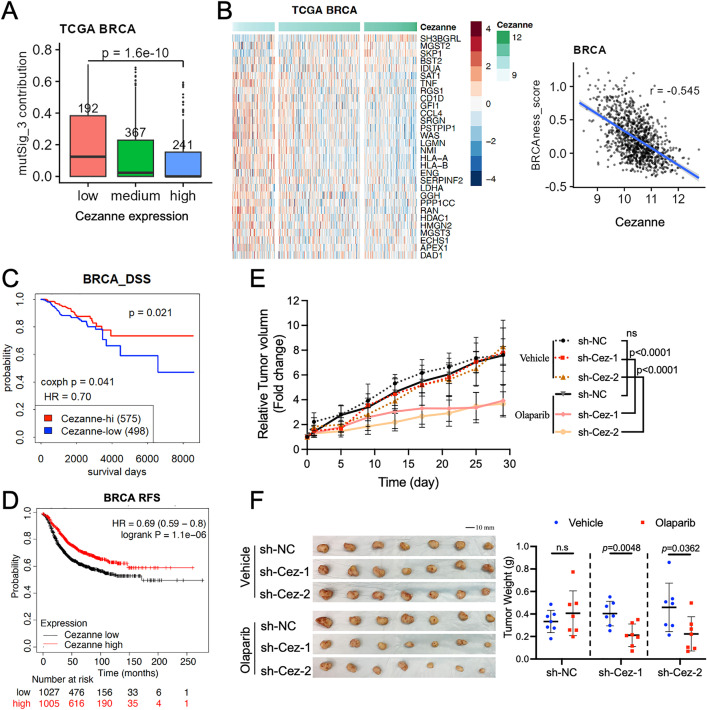
Downregulation of Cezanne is linked with BRCAness and associated with poor prognosis in human cancers. **(A)** Contribution of the mutational signature 3 (associated with HR deficiency) in TCGA BRCA patients stratified by Kmeans 3 separation of Cezanne expression levels (patient numbers as depicted). *P* value was generated by Student *t* test of low and high groups. **(B)** Correlation of Cezanne expression to a 30-gene BRCAness signature in breast tumors. Left, heatmap of BRCAness signature genes in TCGA BRCA tumors ordered by Cezanne expression. Right, scatter plot of Cezanne expression against BRCAness signature scores. Pearson correlation coefficient is shown. **(C)** Kaplan–Meier disease-specific survival (DSS) from TCGA BRCA patients stratified using Kmeans 2 separation of Cezanne expression levels. Cox proportional hazards regression analyses (coxph *p* value and hazard ratio) are also shown. **(D)** Kaplan–Meier relapse-free survival (RFS) plot of breast cancer patients stratified by Cezanne expression level by median using the KM Plotter. Affymetrix ID229488_at was used for analyses. Data were from breast cancer patients with low (*n* = 1,027) and high (*n* = 1,005) Cezanne expression. Patient number at risk at different times of analyses is indicated at the bottom of the plots. Similar results were obtained with Affymetrix ID227436_at and 1555139_at. **(E)** Tumor growth curve of vehicle- or Olaparib-treated xenograft breast tumors formed by injection of control or Cezanne shRNAs-expressing MDA-MB-231 cells. Mice (*n* = 7) were randomly assigned to groups treated with vehicle or olaparib (50 mg/kg/day intraperitoneally). Tumor size was measured every 4 days and relative tumor volume fold change was quantified and shown with mean ± SD. Two-way Anova with Turkey’s multiple comparisons test was used for statistics. **(F)** Reduced tumor growth of Cezanne-deficient breast tumors after olaparib treatment. Tumor (*n* = 7) weight from indicated groups was measured at the end of the treatment and shown with mean ± SD. Student *t* test was used for statistics. The data underlying the graphs shown in the figure can be found in S1 Data.

Previously, we have shown that cells depleted of Cezanne are defective in HR using a reporter assay [14]. Since Cezanne-deficiency leads to decreased BRCA1 levels, we reasoned that breast tumors with low expression of Cezanne may be associated with HR deficiency displaying “BRCAness” in tumors. To determine if this is the case, we examined breast cancer data from the Cancer Genome Atlas (TCGA) on both mutational and gene expression signatures related to BRCAness. We first queried the prevalence of mutation signature 3, which is indicative of HR deficiency (mSignatureDB, [40,41]. As a control, signature 3 contribution is much increased in BRCA1-deficient tumors versus control [42,43] (S6A Fig). The analyses revealed that there is a significant increase of mutational signature 3 contribution in Cezanne low tumors compared to Cezanne high tumors, implying that Cezanne low expression is associated with HR deficiency (Fig 5A).

Next, we used a 30-gene BRCAness gene expression signature derived from a previous study [44] to calculate BRCAness score for each tumor. As Cezanne expression level increases (indicated by darkened green color, top bar in Fig 5B left panel), the BRCAness-associated genes expression is largely reduced (from brown-red to blue, Fig 5B left panel), revealing a strong negative correlation of Cezanne expression with BRCAness scores in breast cancer (Fig 5B). Similar negative correlations can also be observed in many other tumor types, including cancers from lung, kidney, brain, thyroid, and soft tissues (S6B and S6C Fig). These results indicate that BRCAness is associated with Cezanne low tumors. Since BRCA1-deficiency predicts poor survival, we investigated whether Cezanne expression correlates with patient survival in the TCGA dataset [45]. The analysis of TCGA BRCA dataset showed that Cezanne low expression is associated with poor disease-free survival (Fig 5C). Analysis of breast or lung tumors using online Kaplan–Meier Plotter (https://kmplot.com/analysis/) [46,47] also showed that Cezanne low expression is associated with poor breast cancer relapse-free survival and lung cancer overall survival (Figs 5D and S6D). Interestingly, this association is more profound in estrogen receptor (ER) positive (ER+) breast cancers but minimal in ER negative (ER−) cancers (S6E and S6F Fig). Together, these data indicate that Cezanne low-expression tumors are associated with signatures of dysfunctional HR and BRCAness and correlated with poor prognosis in at least breast and lung cancers.

Lack of BRCA1 and dysfunctional HR lead cells sensitive to PARPi. The breast cancer cell line MDA-MB-231 exhibited increased cell sensitivity to PARPi upon depletion of BRCA1 or Cezanne (S7A Fig). To investigate the role of Cezanne in tumor growth and tumor response to PARPi treatment in vivo, we utilized a murine xenograft model by injecting control or Cezanne-deficient MDA-MB-231 cells into Swiss nude mice followed by treatment with PARPi olaparib. Depletion of Cezanne did not have much effect on the formation of tumors from injected cells. Four weeks after injection when tumors reached about 100 mm^3^ in size, mice were randomly assigned to groups treated with vehicle or olaparib given daily. When treated with vehicle, Cezanne-deficient tumors showed similar growth as the control tumors, indicating that Cezanne-deficiency has little effect on tumor growth. However, whereas the control tumors were resistant to the treatment with Olaparib, Cezanne-deficient tumors were sensitive to olaparib treatment and tumor growth was significantly suppressed when compared to the control (Figs 5E, 5F and S7B). Thus, Cezanne-deficiency sensitizes xenograft tumors to PARPi treatment.

### Upregulation of Ube2S leads to reduced BRCA1 and increased cellular sensitivity to PARPi

**Fig 6:**
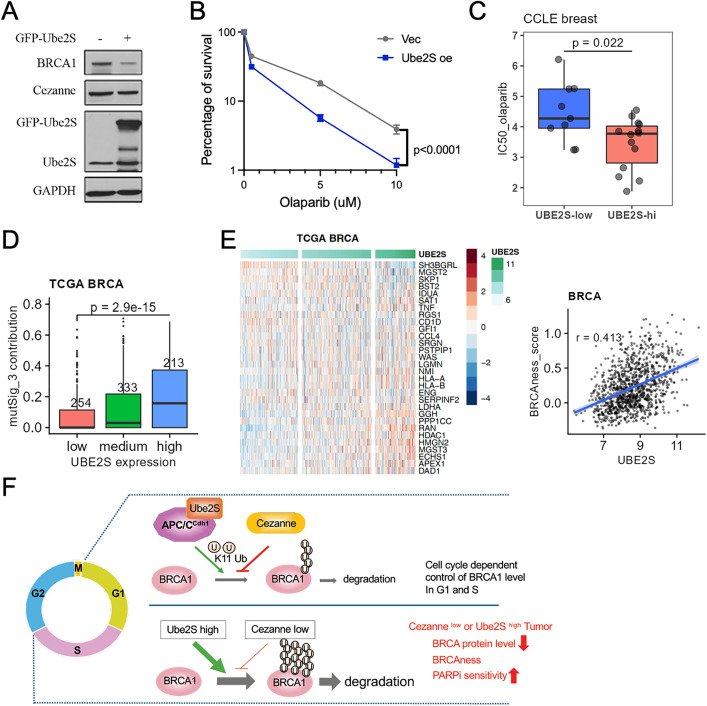
Upregulation of Ube2S leads to reduced BRCA1 and is associated with BRCAness and poor prognosis in human cancers. **(A)** Ube2S overexpression reduces BRCA1 protein level. Total cell lysates from U2OS cells with or without expression of GFP-Ube2S were analyzed with western blots. GAPDH was used as a loading control. Western blots shown are representative of three independent experiments. **(B)** Upregulation of Ube2S in U2OS cells leads to increased cellular sensitivity to olaparib detected by colony formation assay. Quantifications are shown with mean ± SD. Ordinary Two-way Anova was used for statistics. Data are representative of three independent experiments. **(C)** Increased drug sensitivity to olaparib in UBE2S-high breast cancer cell lines. CCLE breast cancer cell lines were stratified by Kmeans 2 separation of UBE2S expression. IC50 data were obtained from GDSC. P value was generated by Student *t* test. **(D)** Contribution of the mutational signature 3 in TCGA BRCA patients stratified by Kmeans 3 separation of Ube2S expression levels (patient numbers as depicted). *P* value was generated by Student *t* test of low and high groups. **(E)** Correlation of Ube2S expression to a 30-gene BRCAness signature in breast tumors. Left, heatmap of BRCAness signature genes in TCGA BRCA tumors ordered by Ube2S expression. Right, scatter plot of Ube2S expression against BRCAness signature scores. Pearson correlation coefficient is shown. **(F)** A proposed model for cell cycle-dependent regulation of BRCA1 protein level in G1 and S phase by the countering activities of APC/C^Cdh1^/Ube2S and Cezanne in K11-linkage specific ubiquitination of BRCA1. Dysregulation of this pathway due to loss of Cezanne or upregulation of Ube2S leads to reduced BRCA1 protein level and is associated with BRCAness in tumors. The data underlying the graphs shown in the figure can be found in S1 Data.

Since increased K11-polyubiquitination of BRCA1 and decreased BRCA1 levels in Cezanne-deficient cells can be restored by simultaneous knockdown of Ube2S (Fig 2F and 2G), we tested whether the increased cell sensitivity to PARPi caused by Cezanne-deficiency can be rescued by Ube2S depletion. Indeed, the increased cellular sensitivity of Cezanne-deficient cells to PARPi in colony survival or cell viability assay was restored to the control level when Ube2S was depleted in U2OS cells (S7C and S7D Fig). This result is also repeated in MDA-MB-231 cells (S7E Fig). Thus, the balanced activity of Ube2S and Cezanne appears to be critical for maintaining the levels of BRCA1 and regulating cellular sensitivity to PARPi. We therefore hypothesize that Ube2S overexpression may tip the balance and lead to deficiency in BRCA1. Indeed, overexpression of GFP-tagged Ube2S decreased BRCA1 protein level in both U2OS and MDA-MB-231 cells and rendered cells more sensitive to olaparib (Figs 6A, 6B, S7F, and S7G). When we examined the drug response data from the Genomics of Drug Sensitivity in Cancer (GDSC, https://www.cancerrxgene.org) along with gene expression data from the Cancer Cell Line Encyclopedia (CCLE) [48], it also showed that breast cancer cell lines with higher UBE2S expression exhibited enhanced sensitivity to olaparib with lower IC50 (Fig 6C). We then analyzed human breast cancer data from TCGA correlating Ube2S mRNA levels with HR-deficient mutational signature 3. We found that Ube2S-high group displayed much more prevalent mutational signature 3, indicative of dysfunctional HR (Fig 6D). In addition, a positive correlation of Ube2S expression with the 30-gene BRCAness gene expression signature was revealed in breast cancer and several additional tumor types (Figs 6E and S8A). Analysis of breast or lung tumors using KM-plotter showed that Ube2S high expression is strongly associated with poor survival in breast and lung cancer (S8B and S8C Fig). It is also noted that the association in breast cancer is more profound in ER+ breast cancers (S8D Fig). Together, these data indicate that dysregulation of K11 polyubiquitination by upregulation of Ube2S leads to reduced BRCA1 protein levels, BRCAness, and increased cellular sensitivity to PARPi.

## Discussion

Our study has identified a Cezanne- and APC/C-Ube2S-regulated K11-linked ubiquitin conjugation pathway that controls BRCA1 protein stability in a cell cycle-dependent manner (**Fig 6F**). It also indicates that perturbation of K11-linked BRCA1 ubiquitination in tumors is linked with BRCAness and may be explored for therapeutic purposes (**Fig 6F**).

### Cell cycle-dependent regulation of BRCA1 stability

Our results demonstrate that Cezanne counteracts the activity of APC/C and Ube2S in K11-ubiquitin conjugation of BRCA1. Loss of Cezanne leads to elevated K11 ubiquitination and degradation of BRCA1 (**Figs 1 and 2**), which is largely reduced when APC/C or Ube2S is inhibited in Cezanne-deficient cells (**Fig 3**). In addition, BRCA1 contains a KEN-box at its N-terminus through which it interacts with Cdh1 and is required for the K11 ubiquitination of BRCA1 (**Fig 3**). Inhibition of APC/C, depletion of Cdh1 or Ube2S also leads to an increase of BRCA1 protein level (**Fig 3**). Therefore, BRCA1 is likely a substrate of APC/C^Cdh1^, susceptible to the regulation of APC/C activity during cell cycle. Our results also suggest that BRCA1 variants in the KEN-box (110-112 aa) may show disrupted binding of BRCA1 to Cdh1 and become more stable in the cell. In a recent study classifying more than 2000 BRCA1 variants at the N-terminus with functional HR repair assays, the variants in the KEN box appear to have normal HR repair function [49]. It is still not clear whether these variants perturb other functions of BRCA1.

APC/C^Cdh1^ becomes active in late mitosis and remains active until the end of G1, leading to the degradation of its substrates during the mitotic exit and G1 [17,18,50]. Our study thus provides a mechanistic illustration for the earlier work which has shown that the steady-state levels of BRCA1 protein decline when cells exit mitosis and progress into G1 phase [1,2]. APC/C^Cdh1^ is inactivated at the G1/S boundary and its activity remains low during the S and G2 phase [17,18,50,51]. This allows for the accumulation of BRCA1 in S and G2 phase as it is for the other substrates of APC/C. DNA repair by HR mainly occurs in S/G2 when sister chromatid is available as a template for error-free recombination, whereas in G1, HR is highly suppressed [52,53]. Low abundance of BRCA1 protein is a key regulatory mechanism necessary to prevent HR in G1. In addition to K11-ubiquitin modification and protein degradation-mediated control of BRCA1 protein level in our study, previous studies have shown that BRCA1 mRNA levels are significantly lower in G1 phase cells [54,55]. A recent study reports that BRCA1 mRNA level is downregulated in G1 through RBM10-mediated RNA alternative splicing coupled with nonsense-mediated mRNA decay [56]. Additionally, it has been shown that in G1, BRCA1-dependent DNA-end resection to generate single-stranded DNA for homology search and strand invasion is inhibited since BRCA1 recruitment to DSBs is suppressed by 53BP1 and RIF1 [57]. Thus, multiple mechanisms exist to inhibit BRCA1 expression and function to suppress HR in G1.

It is interesting that, in addition to G1, Cezanne plays a role in maintaining BRCA1 protein levels in S but not in G2 phase of the cell cycle (**Fig 4**). Ubiquitin conjugation is a reversible process, the equilibrium of conjugation by E2/E3 and deconjugation by DUB is likely critical in determining the levels of ubiquitin modification of substrates. It is noted that Cezanne itself is a cell cycle-regulated protein and its expression is high during mitosis and declines as cells exit mitosis and progress through G1 and into S phase [15]. We speculate that the equilibrium of APC^Cdh1^ E3 and Cezanne DUB activity may be differentially regulated in G1 and S Phase of the cell cycle. In G1, the high APC/C^Cdh1^ activity surpasses the DUB activity of Cezanne, leading to BRCA1 K11-polyubiquitination and protein degradation resulting in low abundance of BRCA1. For S-phase cells, it is possible that the low abundance of Cezanne and the low APC/C activity are kept in balance to maintain appropriate BRCA1 protein levels. Disturbance of the balance by depletion of Cezanne results in excessive BRCA1 K11 ubiquitination and protein degradation. Additional E3 ligases and ubiquitin pathways may also be involved to contribute to the accumulation of BRCA1 in S phase cells. The abundance of BRCA1 in G2 is likely not susceptible to the regulation of K11 ubiquitination pathway since APC/C activity is low and depletion of Cezanne does not have much effect on BRCA1 protein levels in G2. The differential regulation of BRCA1 in S and G2 phase may be further explored in the future.

### Targeting Cezanne- and Ube2S-involved ubiquitination of BRCA1 with PARPi

PARPi therapy has shown promising efficacy in the treatment of *BRCA1/2*-mutant cancer. Tumors with BRCAness—tumors that share molecular features of germline *BRCA1/2*-mutant cancers—may share therapeutic vulnerabilities with *BRCA1/2* mutant cancers to PARPi treatment [35,36]. Our results indicate that dysregulation of the BRCA1 K11-ubiquitin pathway due to reduced Cezanne or elevated expression of Ube2S in tumors is associated with BRCAness and may be targeted for PARPi therapy (**Figs 5 and 6**).

Due to the critical roles of BRCA1 in DNA damage signaling and HR, reduced levels of BRCA1 likely results in BRCA1-deficiency and cells defective in HR. Our data indicate that the regulation of Cezanne on BRCA1 protein stability is independent of DNA damage since it occurs in both non-damaged and IR-treated cells (**Fig 2**). Our previous work has shown that Cezanne plays a role in DNA damage response by removing K11-ubiquitin conjugates on damaged chromatin catalyzed by RNF8 and Ube2S to promote the K63-ubiquitin-dependent recruitment of Abraxas/BRCA1-A complex to DSBs [13,14]. The protein stability of Abraxas or other BRCA1-A complex components aside of BRCA1, however, is not susceptible to the regulation of Cezanne [14]. The Abraxas/BRCA1-A complex plays a critical role in the recruitment of BRCA1 to DSBs and promotion of BRCA1 dimerization at DSBs for tumor suppression [58–63]. Thus, in cells responding to DNA damage, loss of Cezanne compromises BRCA1 function through both decreasing the abundance of BRCA1 and reducing the recruitment of BRCA1 to DNA damage sites. Previously, we have shown that Cezanne-depleted cells are defective in HR and exhibit increased cellular sensitivity to IR [14]. Cezanne-deficient cells are also hypersensitive to PARPi (S7 Fig). Importantly, our analyses of TCGA breast cancer dataset reveal that tumors with low expression of Cezanne exhibit a mutational signature 3 indicative of dysfunctional HR and are associated with a BRCAness signature and correlated with poor prognosis in breast cancer patients (**Fig 5**). Therefore, our data suggest that tumors with low levels of Cezanne may be targeted effectively with PARPi strategy. This is supported by our mice xenograft study showing that tumors with decreased levels of Cezanne are more sensitive to PARPi treatment (**Fig 5**).

Our results also indicate that the balance of K11 ubiquitination of BRCA1 through the countering activities of Ube2S conjugation and Cezanne deconjugation is critical for maintaining BRCA1 protein levels and cellular resistance to PARPi. Depletion of Ube2S reduces the excessive BRCA1 K11 ubiquitination, restores BRCA1 protein level, and rescues cellular sensitivity to PARPi in Cezanne-deficient cells (Figs 2 and S7). It is noted that, in our previous study, loss of Ube2S also rescues the defects of Cezanne-deficient cells in the recruitment of BRCA1-A complex to DNA damage sites [14]. Thus, the countering activities of Cezanne and Ube2S are critical for both the regulation of BRCA1 protein levels and BRCA1 recruitment to DNA damage site. Tipping the balance of K11 ubiquitination by overexpression of Ube2S led to reduced BRCA1 levels and increased cellular sensitivity to PARPi (Fig 6 and S7). Consistently, tumors with higher levels of Ube2S are associated with BRCAness and Ube2S^high^ breast tumor cell lines are more sensitive to PARPi (Fig 6). Together, our study suggests that the levels of Cezanne and Ube2S might be used as biomarkers for prediction of PARPi efficacy in therapy. Currently, it is still not clear whether BRCA1 low level caused by dysregulated Cezanne or Ube2S in tumors is directly linked with poor patient prognosis. Since Cezanne and Ube2S are likely to have multiple substrates, additional pathways may also be involved in the reduced survival among patients with Cezanne^low^ or Ube2S^high^ tumors. It is noted that the poor prognosis associated with Cezanne^low^ or Ube2S^high^ tumors are more prominent in ER+ breast tumors, whereas most *BRCA1*-mutated breast cancers are ER-. It remains to be studied whether Cezanne^low^ or Ube2S^high^ tumors exhibit features similar to that of a subset of BRCA1-deficient ER+ breast tumors [64–66]. Additionally, due to the opposing enzymatic activities of Cezanne and Ube2S, both amount of Cezanne and Ube2S may need to be considered for prediction of PARPi efficacy in tumors since the effect of Cezanne low in tumors on PARPi sensitivity may be nullified by simultaneous low level of Ube2S.

In summary, our study has illustrated a ubiquitin K11-linked modification pathway that regulates BRCA1 protein stability through Cezanne counteracting the activity of APC/C^Cdh1^ and Ube2S during cell cycle. It suggests that tumors with dysregulation of K11-linked ubiquitination of BRCA1 due to reduced Cezanne or elevated Ube2S levels may be effectively targeted by PARPi therapy.

## Materials and methods

### Cell culture and transfection

U2OS cells were grown in McCoy’s 5A with L-glutamine medium (Corning, Cat# 10-050-CV) with 10% fetal bovine serum (Gibco, Cat# 10437-028). 293T, Hela and MDA-MB-231 cells were maintained in Dulbecco’s modified Eagle’s medium (DMEM) (Corning, Cat# 10-013-CV) with 10% fetal bovine serum. A549 and H1299 were grown in RPMI 1640 medium with L-glutamine medium (Corning, Cat# 10-040-CV) with 10% fetal bovine serum (FBS). Lipofectamine RNAiMAX Transfection Reagent (Thermo Fisher, Cat# 13778150) was used for transfection based on manufacture’s protocol. For plasmid transfection, Lipofectamine 2000 Transfection Reagent (Thermo Fisher, Cat# 11668019) was used based on manufacture’s protocol. For generation of stable cell lines expressing control or shRNAs against Cezanne, cells were infected with lentivirus containing corresponding shRNA constructs followed by selection with puromycin (1 µg/ml) for about one week. For generation of inducible shCdh1 stable cell line, U2OS cells were infected with lentivirus expressing shRNA against Cdh1 under a tetracyclin-inducible promoter and selection was performed with puromycin (1 µg/ml). Doxcyline (2 µg/ml) was added for induction of the Cdh1 shRNA. Cezanne KO U2OS cell line was previously generated [14]. For Cycloheximide-chase assay, cells were treated with 100 μg/ml cycloheximide (Sigma-Aldrich, C7698). For MG132 treatment, cells were treated with 20 µM MG132 (Sigma, Cat# 474790) for 6 h before harvest. For the effect of APC/C inhibitor proTAME, cells were treated with proTAME (Boston Biochem, Cat#I-440) (25 µM) for 18 h.

### Plasmids, siRNAs, shRNAs, and antibodies

Flag-tagged wildtype BRCA1 plasmid is a gift from Dr. Yanfen Hu [5]. BRCA1 mutants in the KKEN region were generated by site-directed mutagenesis using a QuikChange II site-directed mutagenesis kit (Agilent Technologies). Primers used for the mutagenesis are:

4A fw: ATGATAGAAACTTCATCTTTTAGATGTTCAGGAGAGTTAGCTGCCGCTGCTGCAAAATTATAGCTGTTTGCATACTCCAAACCTGTGTC
rev: GACACAGGTTTGGAGTATGCAAACAGCTATAATTTTGCAGCAGCGGCAGCTAACTCTCCTGAACATCTAAAAGATGAAGTTTCTATCAT,

GFP-tagged Cezanne WT and DUB-inactive CH (C194S/H358R) mutant expression constructs were previously published [14]. HA-tagged-WT, K11-, K48, or K63 Ub were previously published [13,14]. For generation of GFP-CDH1(Fzr1) expression construct, pENTR-CDH1(Fzr1) was purchased from the MDACC shRNA and ORFeome Core and recombined into MSCV-GFP retroviral expression vector by LR recombination.

A scrambled siRNA (Dharmacon, D-001810-01-20) was used as the control (siNC). siRNAs targeting Cezanne, Ube2S, Ube2C and APC2 were published previously [13,14], including: Cezanne siRNA#1, 5′-AGGUCUCUCUCUAUGAAGC-3′, siRNA#2, 5′-CUUCUGUGUAUACCAGCCC-3′; Ube2S siRNA#1, 5′-GGUCUUUCCCAACGAGGAG-3′, siRNA#2, 5′-CAAGGAGGUGACGACACUG-3′; Ube2C siRNA 5′-CCUACUCAAAGCAGGUCAC-3′; APC2 siRNA#1, 5′-GAGAUGAUCCAGCGUCUGUUU-3′, siRNA#2, 5′-GACAUCAUCACCCUCUAUAUU-3′; siRNF8, 5′-GGGUUUGGAGAUAGCCCAAGGAGAA-3′. Lentivial shRNA plasmids were purchased from the MDACC shRNA and ORFeome Core. Cezanne shRNA#1, TGAGCAAGGACAAAGACGT was previously published [14]. Additional shRNAs used are: Cdh1 shRNA, TCTGTGAGTAGCCGTGCGT; Cezanne shRNA#2, TCATCTTCTGTGTATACCA, shRNA#3, TGCTATAGGAATCAGCCAC, shRNA#4, CTCTCTCTATGAAGCTGCG. The inducible lentiviral vector used for generation of Cdh1 shRNA inducible expression under a tetracycline-inducible promoter is previously published [67].

Antibodies used are: BRCA1 (Cat#sc-6954, Santa Cruz), Cezanne (Cat#sc-514402, Santa Cruz), Ube2S (Cat#9630, Cell Signaling), Ube2C (Cat#WH0011065M1, Sigma), HA (Cat#9661S, Cell Signaling), Flag (Cat#F7425, Sigma), GFP (Cat#A11122, Invitrogen), Cyclin B1 (Cat#sc245, Santa Cruz), Cyclin A (Cat#sc596, Santa Cruz), CDH1 (Invitrogen, Cat#34-2000), APC2 (Cell Signaling, Cat#12301), BARD1 (Proteintech, Cat#22964-1AP).

### Quantitative real-time PCR (qPCR)

Total RNA was isolated from cells using the Vezol-Pure Total RNA Isolation Kit (Vazyme), and cDNA was synthesized from 1 µg of total RNA using iScript cDNA synthesis kit (Bio-Rad). Fluorescence real-time PCR analysis was conducted using Taq Pro Universal SYBR qPCR Master Mix (Vazyme) and performed on the Bio-Rad C1000 thermal cycler (CFX-96 real-time PCR detection systems; Bio-Rad). Primers used for BRCA1 were previously described [56]: 5′-CAACATGCCCACAGATCAAC-3′ and 5′-ATGGAAGCCATTGTCCTCTG-3′. β-actin was used as an internal control with primers 5′-CACAGAGCCTC GCCTTTGCC-3′ and 5′-ACCCATGCCCACCATCACG-3′. Relative expression was calculated using the ΔΔCt method.

### Cell lysis and immunoprecipitation

Cells were lysed using NETN buffer (50 mM Tris-HCl at pH 8.0, 150 mM sodium chloride, 1 mM EDTA, 0.5% nonidet P-40, 1 mM dithiothreitol [DTT], 1 mM PMSF, 5 mM NaF, 1 mM Na3VO4, 50 mM β-glyceral, protease inhibitor cocktails (Roche) and centrifuged at 15,000 rpm for 10 min at 4 °C. The supernatant was used as total cell lysate. For immunoprecipitation, cells were lysed in IP-lysis buffer (50 mM Tris–HCl, pH 7.6; 150 mM NaCl; 2.5 mM CaCl_2_; 2.5 mM MgCl_2_; 0.5% NP-40; 1 mM phenylmethylsulfonyl fluoride) with DNaseI (10 U/ml, ThermoFisher Scientific, Cat#90083). The supernatant was collected and used for an incubation with Flag- or HA-beads or indicated antibodies followed by protein A/G beads (Millipore) at 4 °C overnight. After incubation, beads were washed with IP-lysis buffer for 4 × 15 min. Samples were eluted by 2× SDS-PAGE sample loading buffer at 95 °C for 5 min. For immunoprecipitation under denaturing condition, cells were lysed in denaturing lysis buffer (20 mM Tris, pH 7.4, 50 mM NaCl, 0.5% Nonidet P-40, 0.5% deoxycholate, 0.5% SDS, 1 mM EDTA, protease inhibitor and 20 mM N-ethylmaleimide). The lysate was sonicated on ice and centrifuged at 21,000*g* for 10 min at 4 °C. The supernatant was collected and diluted 5× with IP lysis buffer (SDS was diluted to 0.1% in the lysis buffer) for immunoprecipitation with indicated antibodies at 4 °C overnight.

### In vitro deubiquitinating (DUB) assay

Flag-BRCA1 was purified from 293T cells transiently transfected with both Flag-BRCA1 and Myc-K11 Ub plasmids for 48 h and treated with MG132 for 6 h under denaturing condition as described above. Flag beads were used for immunoprecipitation. Flag IP under denaturing condition was also performed with 293T cells expressing an empty vector and was used as negative controls in the DUB assay. HA-Cezanne WT and CH mutant were immunoprecipitated from 293T cells transiently transfected with the indicated plasmids respectively. After HA immunopriciptation and washes, HA-Cezanne WT and CH mutant protein were eluted from HA beads with 1 mg/ml HA peptides 2 × 15 min at 4 ℃. For in vitro DUB assay, purified WT or CH mutant Cezanne was added to the reaction with K11 ubiquitinated Flag-BRCA1 in DUB buffer (50 mM Tris-HCl, pH 7.4, 5 mM MgCl_2_, 50 mM NaCl, and 5 mM DTT) at 37 °C with rotation for 1 h. After the reaction, Flag beads were collected and washed with DUB buffer for 3 × 10 min. 2× SDS-PAGE sample loading buffer were then added and incubated at 95 °C for 5 min.

### Cell cycle synchronization

Nocodazole arrest was used to synchronize cells at G2/M phase. Cells were treated with nocodazole (200 ng/ml) for 24 h. For cell cycle progression during mitotic exit and progression into G1 phase, nocodazole-arrested cells were washed with warm medium and released into fresh medium and collected at indicated times for analyses. Double-thymidine block was used to synchronize cells at G1/S. Cells were treated with 2 mM thymidine for 16 h, washed and released into fresh medium for 8 h, followed by a second thymidine (2 mM) block for 16 h. For cell cycle progression after G1/S, double-thymidine-treated cells were washed and released into fresh medium and collected at indicated times for analyses. For synchronizing U2OS cells at G1/S, cells were first serum-starved for 24 h before the double-thymidine block described above was performed. For the experiment assessing K11- or K48-ubiquitination of BRCA1 throughout cell cycle, A549 cells transfected with HA-tagged K11- or K48 ubiquitin were synchronized at G1/S using double-thymidine block, washed with warm medium, and released into fresh medium containing nocodazole (200 ng/ml) for 10 h. The cells were then washed with warm medium and released into fresh medium for an additional 4 h. Cells at indicated time cell cycle phase G1/S (0 h), S (4 h), G2/M (10 h), and next G1 (14 h) were collected for analyses. The distribution of cell cycle was determined by staining cells with propidium iodide (PI) and analyzed by flow cytometry.

### Clonogenic survival and cell viability assay

For clonogenic survival assay, cells were plated at low density and cultured in medium containing indicated doses of Olaparib. The medium was changed every 2 days for about 2 weeks. Colonies were fixed and stained with 2% methylene blue and 50% ethanol. Colony formation efficiency was normalized to the non-treated control for the calculation of the percentage of survival. Cell viability assay was carried out using Cell Counting Kit- 8 (CCK-8) kit (Dojindo). 1,000 ~ 2,000 indicated cells were seeded into each well of a 96-well plate. After overnight incubation, cells were cultured in medium with indicated doses of Olaparib. The assay was performed following the manufacturer’s instructions and the plates were analyzed with CLARIOstar (BMG LABTECH) at 450 nm to measure the absorbance. The experiment was repeated at least three times.

### Mice xenograft study

Mice were housed and handled in accordance with protocol 000012470RN03 approved by the Institutional Animal Care and Use Committee (IACUC) of the MD Anderson Cancer Center. A total of 2 * 10^6^ control (sh-NC) or Cezanne-depleted (sh-Cezanne-1 and sh-Cezanne-2) MDA-MB-231 cells diluted in 100 μl of 1:1 Matrigel/phosphate-buffered saline (PBS) were inoculated subcutaneously into right flank of 5-week-old female Swiss nude mice. After 4 weeks, mice were randomly assigned to vehicle or Olaparib treatment groups. Olaparib (50 mg/kg/day intraperitoneally) were given for consecutive 28 days. Tumor size was measured every 4 days for 4 weeks and tumor volume was calculated by formula: *V* = *L* * *W*^2^/*2* (*L*: long diameter of the tumor; *W*: short diameter of the tumor). Tumor weight at the end of the treatment was measured.

### TCGA datasets

TCGA RNA-Seq and methylation data were downloaded from Broad Institute Firehose website (https://gdac.broadinstitute.org/, last accessed 2019). Patient survival data were obtained from a previous TCGA pan-cancer clinical data study [45].

### Correlation of mutational signatures to Cezanne and UBE2S expression levels

BRCA mutation signature scores were downloaded from mSignatureDB (http://tardis.cgu.edu.tw/msignaturedb) [40]. Breast cancer tumors were grouped into low, medium, and high expression groups based on Cezanne or UBE2S RNA-Seq data using Kmeans function of R software.

### Correlation of the BRCAness gene expression signature to Cezanne expression levels

Gene expression of a reported 60-gene BRCAness gene expression signature from a previous ovarian cancer study [44] was examined in TCGA BRCA samples grouped by BRCA1 and BRCA2 deficiency status (mutation, deletion, hyper-methylation, and lowest expressions versus wild type) as well as breast cancer subtypes (basal versus luminal) to narrow down signature to 30 top genes for better reflecting differences in BRCA status (APEX1, BST2, CCL4, CD1D, DAD1, ECHS1, ENG, GFI1, GGH, HDAC1, HLA-A, HLA-B, HMGN2, IDUA, LDHA, LGMN, MGST2, MGST3, NMI, PPP1CC, PSTPIP1, RAN, RGS1, SAT1, SERPINF2, SH3BGRL, SKP1, SRGN, TNF, WAS). A BRCAness signature score was then calculated as the sum of MAD-based Z scores of 30 signature genes for each tumor sample.

### Prognostic value of Cezanne and UBE2S genes in breast cancers

Survival analysis was carried out using the online KM-plotter resource (https://kmplot.com/analysis/) [46,47] as well as TCGA BRCA RNA-Seq and clinical data. For KM-plotter, all breast cancer cases, or ER status by array, were used and all microarrays probes for Cezanne and UBE2S were tested using median separation of patients. For TCGA, disease-specific survival (DSS) was used, samples were stratified by Kmeans 2 separation of Cezanne or UBE2S gene expression levels. KM plots were generated using the R survival package.

### Correlation of Cezanne and UBE2S expression levels to PARP inhibitor sensitivity in CCLE cell lines

Cancer cell line drug response data were downloaded from GDSC website (https://www.cancerrxgene.org/downloads/bulk_download, v17_fitted_dose_response). Cell line gene expression data (TPM) were downloaded from CCLE (https://sites.broadinstitute.org/ccle/, last accessed 2019). Cell lines of breast tissue type were selected and stratified into UBE2S high and low expression groups based on Kmeans 2 separation using UBE2S gene expression TPM values. Box plots were made for grouped cell lines having olaparib treatment data (GDSC drug ID 1495), and tested using Student *t* test to examine IC50 differences between groups.

### Statistics

Statistical analysis was performed with GraphPad Prism 10. One-way Anova, two-way Anova, or *t* test was used for statistical analyses as indicated. *P* < 0.05 was considered statistically significant. Visualization of gene expression data by heatmaps and other plots including box plots and scatter plots were done using R software (R v4.0.5) packages pheatmap (v1.0.12) and ggplot2 (v4.0.0).

## Supporting information

S1 Fig Cezanne regulates BRCA protein level. **(A, B)** Immunoblots confirming Cezanne knockdown reduces BRCA1 protein level in cells. **(C)** Cezanne knockdown reduces BRCA1 protein level in cells untreated or treated with ionizing radiation (IR). “siCezanne-Mix” is a mixture of siCezanne-1 and 2. **(D)** Treatment of proteasome inhibitor MG132 (20 µM, 6 h) restores BRCA1 level in Cezanne knockdown cells. **(E)** qPCR analysis of BRCA1 mRNA levels in indicated siRNAs-treated cells (*n* = 4) in various cell lines. **(F)** Cezanne C-terminus zinc finger (ZF) domain is critical for Cezanne interaction with BRCA1. BRCA1 IP was performed from lysates of 293T cells expressing HA-tagged Cezanne WT or different deletion mutants. A diagram of the Cezanne deletion mutants (top panel) and western blots with indicated antibodies (lower panel) are shown. “*”indicates band with correct size. The data underlying the graphs shown in the figure can be found in S1 Data.
(TIFF)

S2 Fig Cezanne regulates BRCA1 K11-linked ubiquitination. **(A)** Cezanne knockdown does not affect K63- or K48-linked ubiquitination of BRCA1. U2OS cells transfected with HA-tagged K11-, K48- or K63-Ub were subsequently transfected with indicated siRNAs. Cells were then treated with MG132 (20 µM, 6 h) before harvest. BRCA1 IP was performed under denaturing condition. **(B)** Increased K11- ubiquitination of BRCA1 in Cezanne depleted cells untreated or treated with IR (10 Gy, 2 h). Cells were treated with MG132 (20 µM, 6 h) before harvest. BRCA1 IP was performed under denaturing condition. **(C)** Increased K11-ubiquitination of BRCA1 in Cezanne depleted A549 and MDA-MB-231 cells. Cells were treated with MG132 (20 µM, 6 h) before harvest. **(D)** A silver-staining gel showing purified Flag-BRCA1 on Flag beads purified under denaturing condition from 293T cells expressing both Flag-BRCA1 and myc-K11 Ub. **(E)** Immunoblot of an in vitro DUB assay showing that Cezanne WT but not CH mutant deubiquitinates K11 Ub modified BRCA1. Purified Flag-BRCA1 shown above was used in the reaction with HA-tagged Cezanne WT and CH mutant immunoprecipitated from 293T cells expressing HA-Cezanne WT or CH respectively. **(F)** Ube2S knockdown leads to an increase of BRCA1 protein level. Total lysates were examined by western blots. Relative amount of BRCA1 is measured by Image J and quantified from three independent experiments (*n* = 3). The data underlying the graph shown in the figure can be found in S1 Data
(TIFF)

S3 Fig BRCA1 K11 ubiquitination is regulated by APC/C^Cdh1^. **(A)** RNF8 depletion does not lead to an increase of BRCA1 levels. **(B)** RNF8 depletion does not restore BRCA1 protein level in Cezanne-deficient cells. **(C)** APC/C inhibitor treatment restores BRCA1 protein level in Cezanne-depleted cells. U2OS cells transfected with indicated siRNAs were either untreated or treated with proTAME (25 µM, 18h). Relative amount of BRCA1 is measured by Image J and quantified from three independent experiments. **(D)** APC/C inhibitor, ProTAME, treatment leads to an increase of BRCA1 levels. Relative amount of BRCA1 is measured by Image J and quantified from three independent experiments. **(E)** Knockdown of APC2 leads to an increase of BRCA1 protein level. Relative amount of BRCA1 is measured by Image J and quantified from three independent experiments. **(F)** BRCA1 possesses KEN box at its N-terminus. Alignment of BRCA1 KEN box sequence from human and several additional species. **(G)** BRCA1 4A mutant interacts with BARD1. 293T cells expressing Flag-BRCA1 WT or 4A mutant were used for BARD1 IP or Flag IP. **(H)** BRCA1 C61G mutant interacts with Cdh1. 293T cells expressing GFP-tagged BRCA1 WT or C61G mutant were used for GFP IP. The data underlying the graphs shown in the figure can be found in S1 Data
(TIFF)

S4 Fig Regulation of BRCA1 by Cezanne is cell cycle dependent. **(A)** Flow cytometry analyses of control or Cezanne knockdown U2OS cells synchronized in G1, S and G2/M phase of the cell cycle shown in Fig 4A. G1 cells were collected at 4 h after release from nocodazole arrested cells; S cells were collected at 4 h after release from double thymidine block; G2/M cells were collected from nocodazole arrested cells. **(B)** Cell cycle distribution of control or Cezanne knockdown U2OS cells released from nocodazole treatment at indicated times in Fig 4B. **(C, D)** BRCA1 protein level reduction in Cezanne-deficient A549 cells during mitotic exit to the G1 phase of cell cycle. Western blots of control or Cezanne knockdown cells released from nocodazole arrested cells at indicated times are shown (C). Cell cycle distribution of cells is shown (D). **(E)** Cell cycle distribution of double thymidine synchronized A549 cells and cells released into fresh medium containing nocodazole at indicated times in Fig 4C. **(F, G)** Reduced BRCA1 protein level in G1/S and S phase Cezanne-deficient Hela cells. Hela Cells were synchronized in G1/S with double thymidine block and released into medium containing nocodazole at indicated times (F). Cell cycle distribution is shown in (G).
(TIFF)

S5 Fig Cezanne regulates BRCA1 K11—but not K48—ubiquitination in G1 and S phase of the cell cyle. **(A)** Cell cycle distribution of control or Cezanne knockdown A549 cells expressing HA-K11 Ub synchronized in G1/S, S, G2/M and the next G1 phase of the cell cycle shown in Fig 4D. **(B, C)** Cezanne does not regulates BRCA1 K48-ubiquitination throughout the cell cycle. A549 cells expressing HA-K48 Ub and transfected with indicated siRNAs were synchronized following the illustrated scheme in Fig 4D. Cell cycle distribution of control or Cezanne knockdown A549 cells synchronized in G1/S, S, G2/M and the next G1 phase of the cell cycle are shown (B). BRCA1 IP was performed under denaturing condition (C).
(TIFF)

S6 Fig Down regulation of Cezanne is linked with BRCAness and associated with poor prognosis in human cancers. **(A)** Mutational signature 3 contribution in BRCA1 deficient breast cancer. TCGA BRCA tumors were grouped into different categories based on BRCA1 deletion, promoter methylation, mutation, and low expression status and compared to the remaining tumors (considered as BRCA1 wild type) for mutational signature 3 contribution. BRCA1 deletion and promoter methylation tumors were determined by TCGA copy number and DNA methylation data, respectively. BRCA1 mutation cases were determined using TCGA gene mutation data. A cutoff for BRCA1 low expression tumors was determined by the BRCA1 expression level in BRCA1 deletion and methylation cases. **(B)** Correlation of Cezanne expression to a 30-gene BRCAness signature in lung adenocarcinomas (LUAD). Left, heatmap of BRCAness signature genes in TCGA LUAD tumors ordered by Cezanne expression. Right, scatter plot of Cezanne expression against BRCAness signature scores. **(C)** Negative correlation of Cezanne expression with BRCAness signature in various tumors. Association R score is listed for various tumors. **(D)** Kaplan–Meier overall survival (OS) plot of lung cancer patients stratified by Cezanne expression level by median using the KM Plotter. Affymetrix ID229488_at was used for analyses. Data were from lung cancer patients with low (*n* = 575) and high (*n* = 569) Cezanne expression. Patient number at risk at different times of analyses is indicated at the bottom of the plots. Similar results were obtained with Affymetrix ID227436_at. **(E)** Kaplan-Meier curves showing disease specific survival (DSS) from TCGA BRCA patients separated into ER+ and ER- groups by *K*-means clustering (*k* = 2) of ESR1 gene expression. Within each ER group, patients were further stratified into high and low Cezanne expression using *K*-means clustering (*k* = 2). **(F)** Kaplan–Meier relapse free survival (RFS) plot of breast cancer patients stratified by Cezanne expression level by median and ER status by array using the KM Plotter. Affymetrix ID229488_at was used for analyses. The data underlying the graphs shown in the figure can be found in S1 Data
(TIFF)

S7 Fig Down regulation of Cezanne or upregulation of Ube2S leads to reduced BRCA1 protein level and increased cellular sensitivity to PARPi. **(A)** Depletion of BRCA1 or Cezanne in MDA-MB-231 cells leads to increased cellular sensitivity to PARPi. Immunoblots confirming the effect of BRCA1 or Cezanne knockdown is shown (left panel). Colony survival assay was performed, and quantifications (*n* = 3) are shown with mean ± SD (right panel). Data is a representative of three independent experiments. **(B)** Knockdown of Cezanne in MDA-MB-231 cells used in mice xenograft experiments in Fig 5E. **(C)** Depletion of Ube2S rescues the increased cellular sensitivity of Cezanne-deficient U2OS cells to olaparib detected by colony formation assay. Quantifications (*n* = 3) are shown with mean ± SD. Data is a representative of three independent experiments. **(D)** Depletion of Ube2S rescues the increased cellular sensitivity of Cezanne-deficient cells to olaparib detected by cell viability assay 4-day after treatment with olaparib. Quantifications (*n* = 4) are shown with mean ± SD. Data is a representative of three independent experiments. **(E)** Depletion of Ube2S rescues the increased cellular sensitivity of Cezanne-deficient MDA-MB-231 cells to olaparib detected by colony formation assay. Immunoblots confirming the effect of the depletion of Cezanne or Ube2S and on BRCA1 protein level is shown (left panel). Quantifications (*n* = 3) are shown with mean ± SD. Data is a representative of three independent experiments. **(F, G)** Upregulation of Ube2S in MDA-MB-231 cells leads to reduced BRCA1 protein level and increased cellular sensitivity to PARPi. Immunoblots showing the effect of expression of GFP-Ube2S on BRCA1 protein level is shown in (F). The colony formation assay was performed and quantified (*n* = 3) with mean ± SD in (G). Data are representative of three independent experiments. Two-way Anova with Tukey’s multiple comparisons test was used for statistics in (A) (C) (D) (E). Ordinary Two-way Anova was used for statistics in (G). The data underlying the graphs shown in the figure can be found in S1 Data
(TIFF)

S8 Fig Upregulation of Ube2S is associated with BRCAness and poor prognosis in human cancers. **(A)** Positive correlation of Ube2S expression with BRCAness signature in various tumors. Association R score is listed for various tumors. **(B)** Kaplan–Meier relapse free survival plot of breast cancer patients stratified by Ube2S expression level by median using the KM Plotter. Affymetrix ID202779_s_at was used for analyses. Data were from breast cancer patients with low (*n* = 2,467) and high (*n* = 2,462) Ube2S expression. Patient number at risk at different times of analyses is indicated at the bottom of the plots. **(C)** Kaplan–Meier overall survival plot of lung cancer patients stratified by Ube2S expression level by median using the KM Plotter. Affymetrix ID202779_s_at was used for analyses. Patient number at risk at different times of analyses is indicated at the bottom of the plots. **(D)** Kaplan–Meier relapse free survival (RFS) plot of breast cancer patients stratified by Cezanne expression level by median and ER status by array using the KM Plotter. Affymetrix ID202779_at was used for analyses. The data underlying the graphs shown in the figure can be found in S1 Data
(TIFF)

S1 Raw Images Original western blots images for Figs 1–6 and S1–S8. (PDF)

S1 Data Numerical data used in Figs 1–6 and S1–S8. (XLSX)

## Abbreviations

APC/C: anaphase-promoting complex/cyclosome
CCLE: Cancer Cell Line Encyclopedia
CH: Cezanne
DMEM: Dulbecco’s modified Eagle’s medium
DSBs: double-strand breaks
DUB: deubiquitinating
ER: estrogen receptor
GDSC: Genomics of Drug Sensitivity in Cancer
HR: homologous recombination repair
KO: knockout
OTU: ovarian tumor
PARP: poly(ADP-ribose) polymerases
PARPi: PARP inhibitors
PBS: phosphate-buffered saline
qPCR: quantitative real-time PCR
