# Supplementary figures and images for: The deubiquitinating enzyme Cezanne stabilizes BRCA1 by counteracting APC/C and Ube2S-dependent Lys11-linked ubiquitination
Source: PLoS Biol. 2025 Dec 8;23(12):e3003545. doi: 10.1371/journal.pbio.3003545 (PMC12685207; doi:10.1371/journal.pbio.3003545)

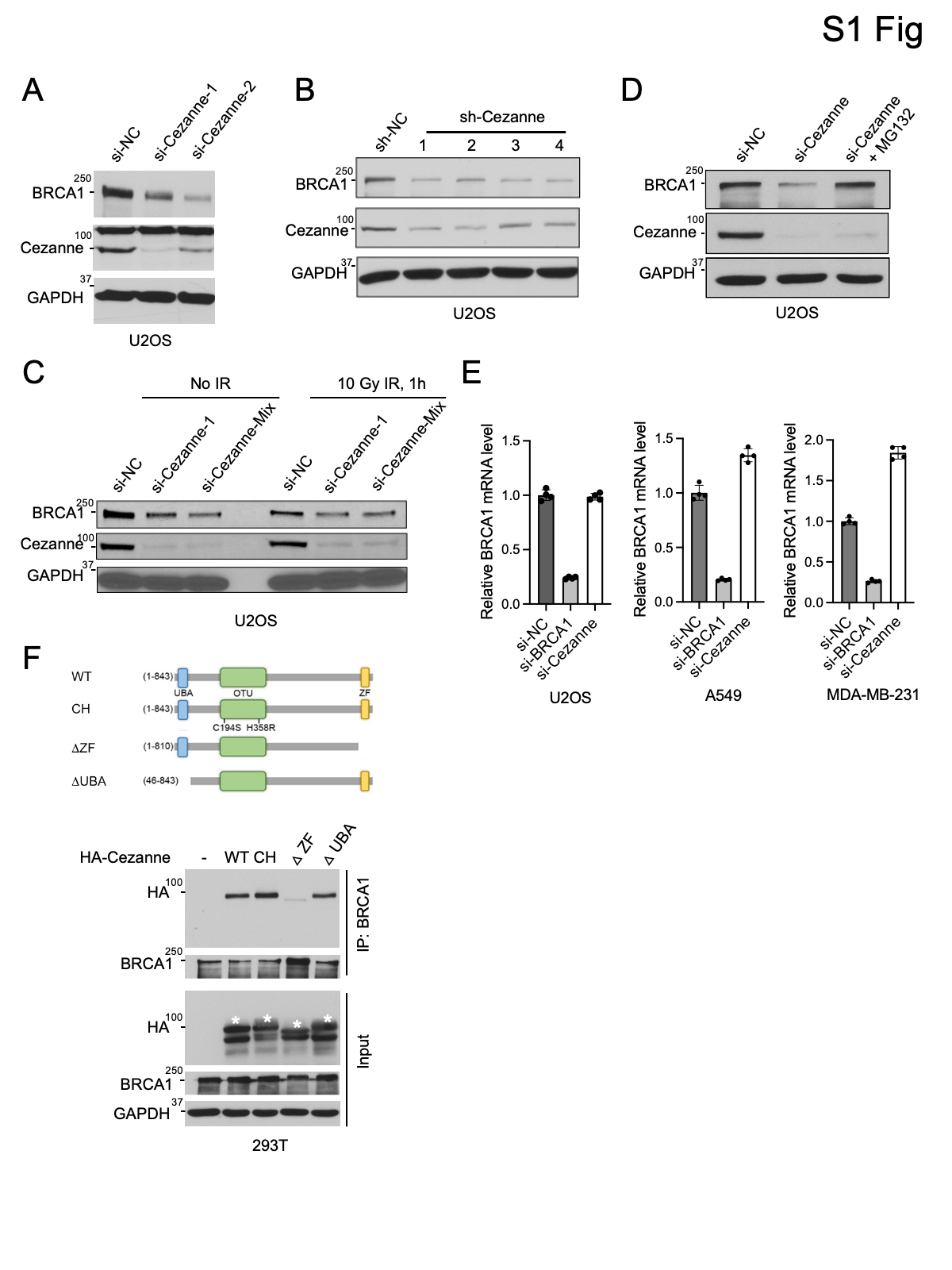

Supplement: S1 Fig — (A, B) Immunoblots confirming Cezanne knockdown reduces BRCA1 protein level in cells. (C) Cezanne knockdown reduces BRCA1 protein level in cells untreated or treated with ionizing radiation (IR). “siCezanne-Mix” is a mixture of siCezanne-1 and 2. (D) Treatment of proteasome inhibitor MG132 (20 µM, 6 h) restores BRCA1 level in Cezanne knockdown cells. (E) qPCR analysis of BRCA1 mRNA levels in indicated siRNAs-treated cells (n = 4) in various cell lines. (F) Cezanne C-terminus zinc finger (ZF) domain is critical for Cezanne interaction with BRCA1. BRCA1 IP was performed from lysates of 293T cells expressing HA-tagged Cezanne WT or different deletion mutants. A diagram of the Cezanne deletion mutants (top panel) and western blots with indicated antibodies (lower panel) are shown. “*”indicates band with correct size. The data underlying the graphs shown in the figure can be found in S1 Data. (TIFF) [file pbio.3003545.s001.tiff]

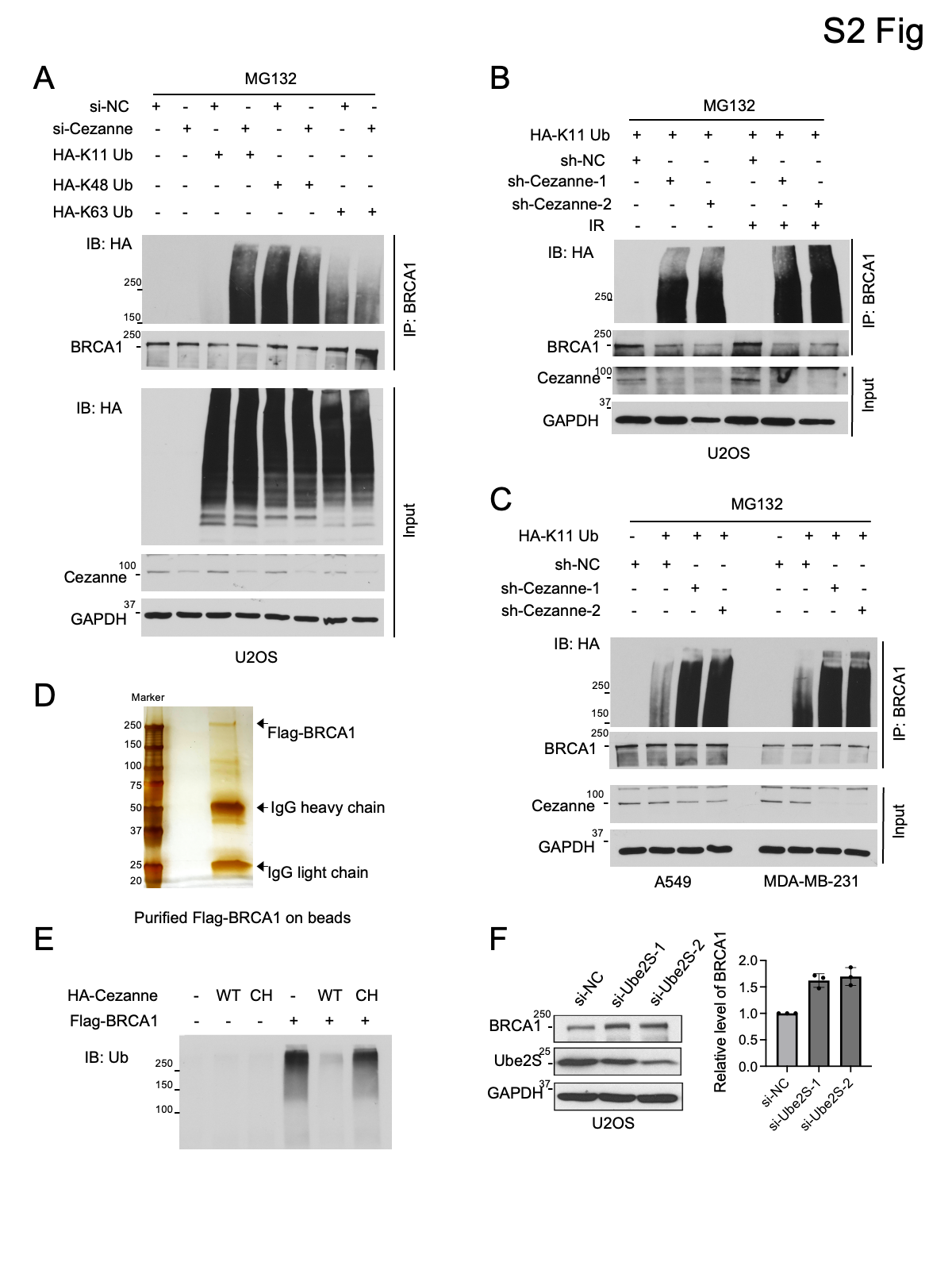

Supplement: S2 Fig — (A) Cezanne knockdown does not affect K63- or K48-linked ubiquitination of BRCA1. U2OS cells transfected with HA-tagged K11-, K48- or K63-Ub were subsequently transfected with indicated siRNAs. Cells were then treated with MG132 (20 µM, 6 h) before harvest. BRCA1 IP was performed under denaturing condition. (B) Increased K11- ubiquitination of BRCA1 in Cezanne depleted cells untreated or treated with IR (10 Gy, 2 h). Cells were treated with MG132 (20 µM, 6 h) before harvest. BRCA1 IP was performed under denaturing condition. (C) Increased K11-ubiquitination of BRCA1 in Cezanne depleted A549 and MDA-MB-231 cells. Cells were treated with MG132 (20 µM, 6 h) before harvest. (D) A silver-staining gel showing purified Flag-BRCA1 on Flag beads purified under denaturing condition from 293T cells expressing both Flag-BRCA1 and myc-K11 Ub. (E) Immunoblot of an in vitro DUB assay showing that Cezanne WT but not CH mutant deubiquitinates K11 Ub modified BRCA1. Purified Flag-BRCA1 shown above was used in the reaction with HA-tagged Cezanne WT and CH mutant immunoprecipitated from 293T cells expressing HA-Cezanne WT or CH respectively. (F) Ube2S knockdown leads to an increase of BRCA1 protein level. Total lysates were examined by western blots. Relative amount of BRCA1 is measured by Image J and quantified from three independent experiments (n = 3). The data underlying the graph shown in the figure can be found in S1 Data (TIFF) [file pbio.3003545.s002.tiff]

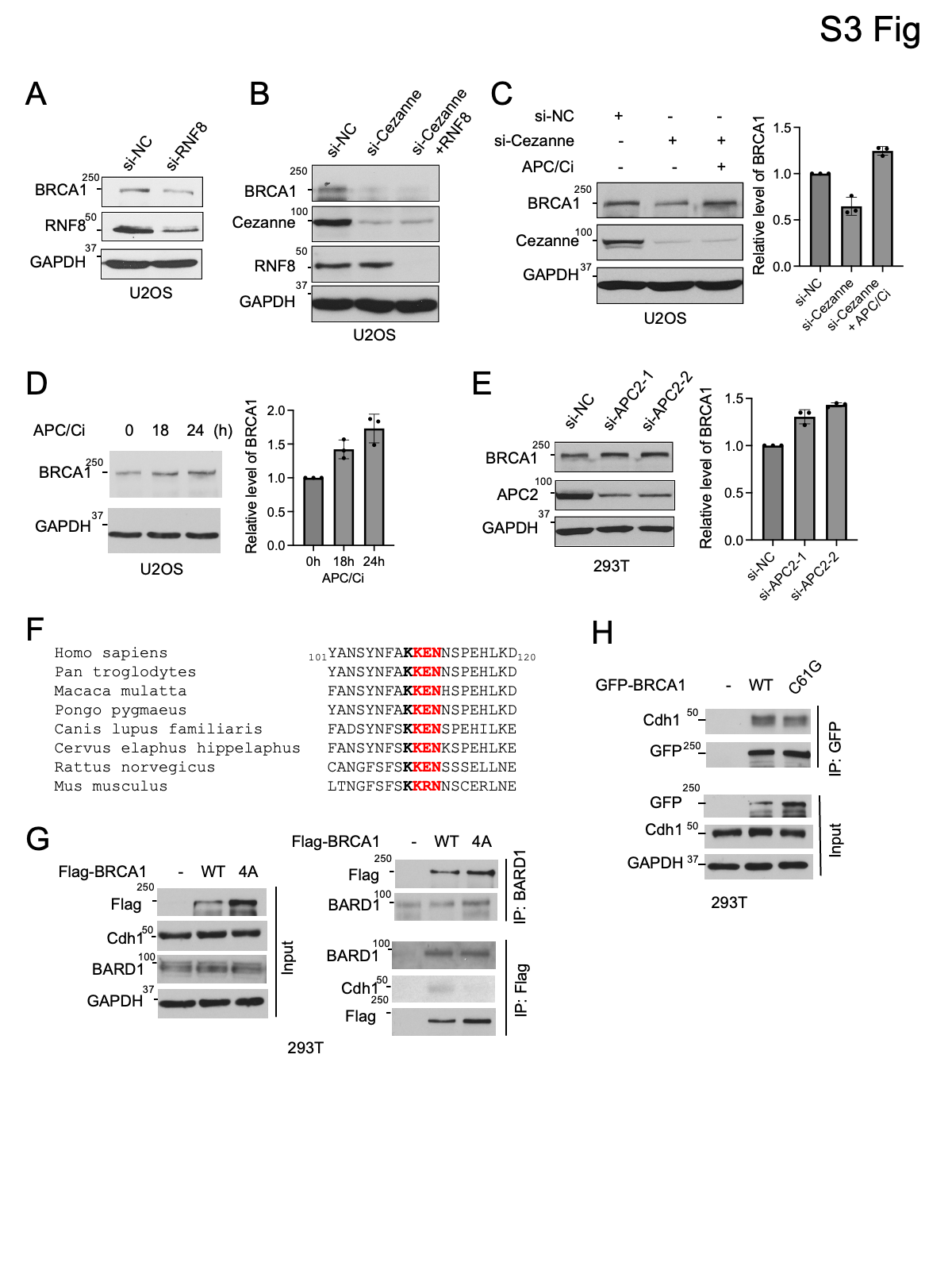

Supplement: S3 Fig — (A) RNF8 depletion does not lead to an increase of BRCA1 levels. (B) RNF8 depletion does not restore BRCA1 protein level in Cezanne-deficient cells. (C) APC/C inhibitor treatment restores BRCA1 protein level in Cezanne-depleted cells. U2OS cells transfected with indicated siRNAs were either untreated or treated with proTAME (25 µM, 18h). Relative amount of BRCA1 is measured by Image J and quantified from three independent experiments. (D) APC/C inhibitor, ProTAME, treatment leads to an increase of BRCA1 levels. Relative amount of BRCA1 is measured by Image J and quantified from three independent experiments. (E) Knockdown of APC2 leads to an increase of BRCA1 protein level. Relative amount of BRCA1 is measured by Image J and quantified from three independent experiments. (F) BRCA1 possesses KEN box at its N-terminus. Alignment of BRCA1 KEN box sequence from human and several additional species. (G) BRCA1 4A mutant interacts with BARD1. 293T cells expressing Flag-BRCA1 WT or 4A mutant were used for BARD1 IP or Flag IP. (H) BRCA1 C61G mutant interacts with Cdh1. 293T cells expressing GFP-tagged BRCA1 WT or C61G mutant were used for GFP IP. The data underlying the graphs shown in the figure can be found in S1 Data (TIFF) [file pbio.3003545.s003.tiff]

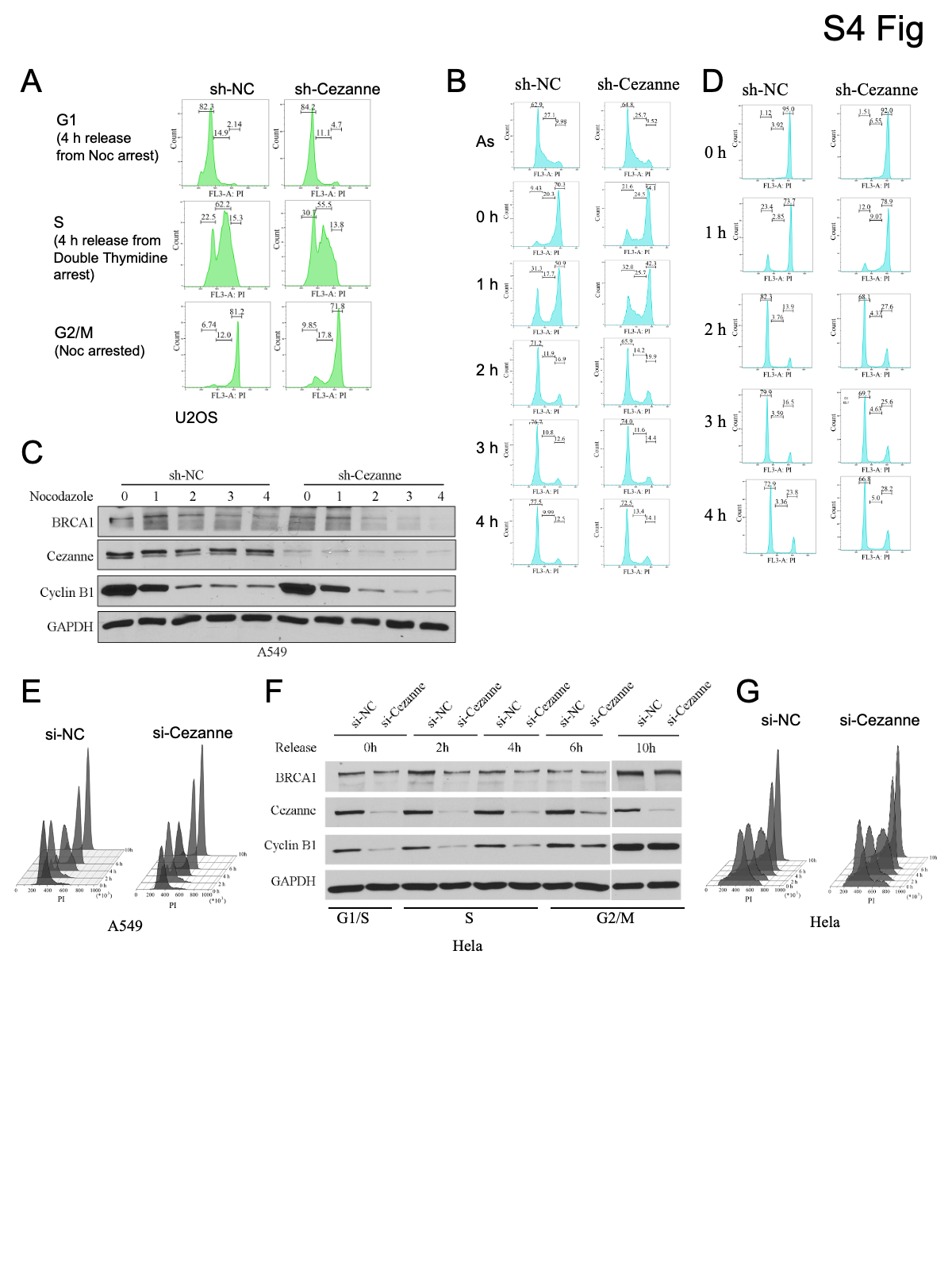

Supplement: S4 Fig — (A) Flow cytometry analyses of control or Cezanne knockdown U2OS cells synchronized in G1, S and G2/M phase of the cell cycle shown in Fig 4A. G1 cells were collected at 4 h after release from nocodazole arrested cells; S cells were collected at 4 h after release from double thymidine block; G2/M cells were collected from nocodazole arrested cells. (B) Cell cycle distribution of control or Cezanne knockdown U2OS cells released from nocodazole treatment at indicated times in Fig 4B. (C, D) BRCA1 protein level reduction in Cezanne-deficient A549 cells during mitotic exit to the G1 phase of cell cycle. Western blots of control or Cezanne knockdown cells released from nocodazole arrested cells at indicated times are shown (C). Cell cycle distribution of cells is shown (D). (E) Cell cycle distribution of double thymidine synchronized A549 cells and cells released into fresh medium containing nocodazole at indicated times in Fig 4C. (F, G) Reduced BRCA1 protein level in G1/S and S phase Cezanne-deficient Hela cells. Hela Cells were synchronized in G1/S with double thymidine block and released into medium containing nocodazole at indicated times (F). Cell cycle distribution is shown in (G). (TIFF) [file pbio.3003545.s004.tiff]

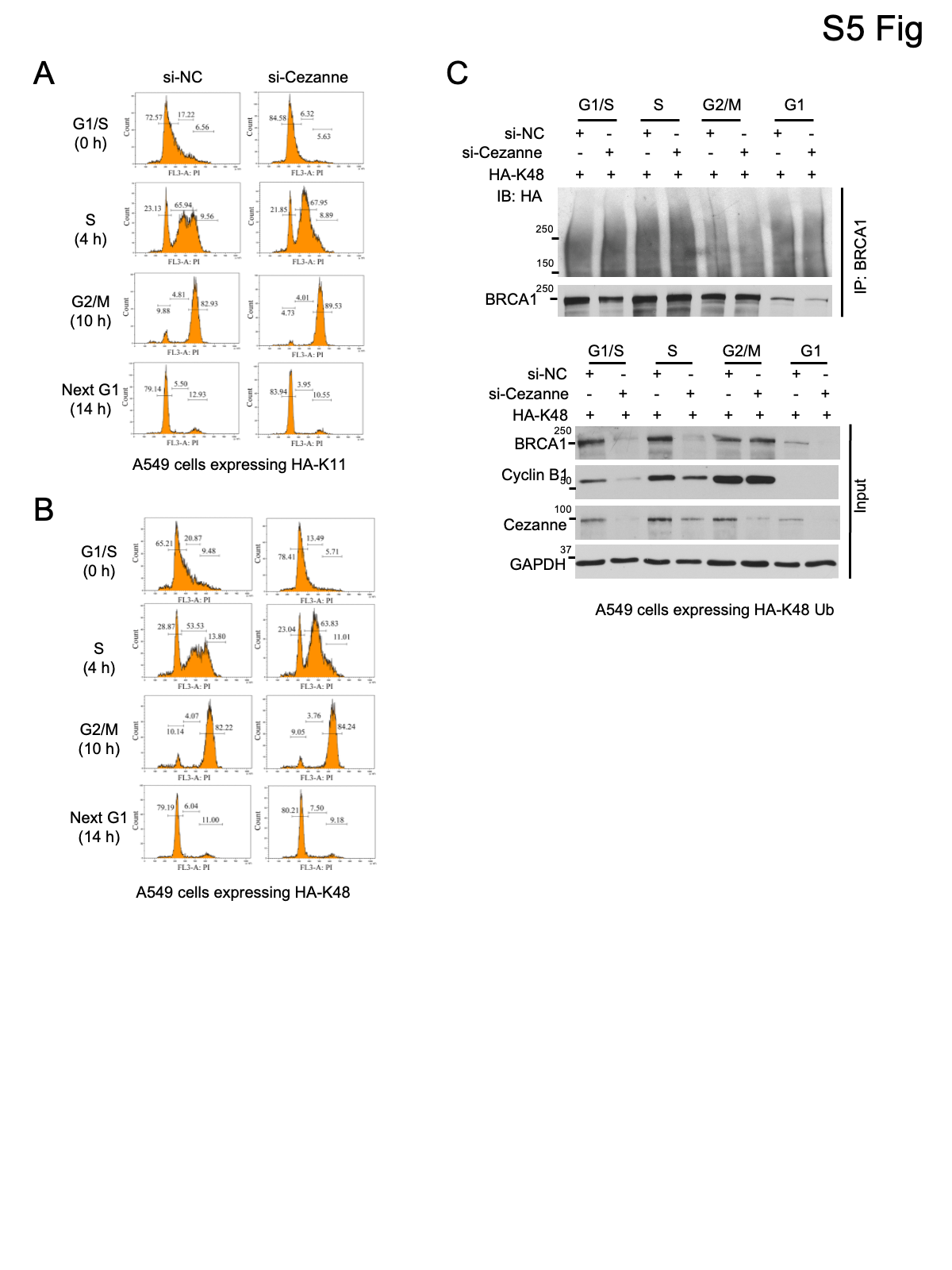

Supplement: S5 Fig — (A) Cell cycle distribution of control or Cezanne knockdown A549 cells expressing HA-K11 Ub synchronized in G1/S, S, G2/M and the next G1 phase of the cell cycle shown in Fig 4D. (B, C) Cezanne does not regulates BRCA1 K48-ubiquitination throughout the cell cycle. A549 cells expressing HA-K48 Ub and transfected with indicated siRNAs were synchronized following the illustrated scheme in Fig 4D. Cell cycle distribution of control or Cezanne knockdown A549 cells synchronized in G1/S, S, G2/M and the next G1 phase of the cell cycle are shown (B). BRCA1 IP was performed under denaturing condition (C). (TIFF) [file pbio.3003545.s005.tiff]

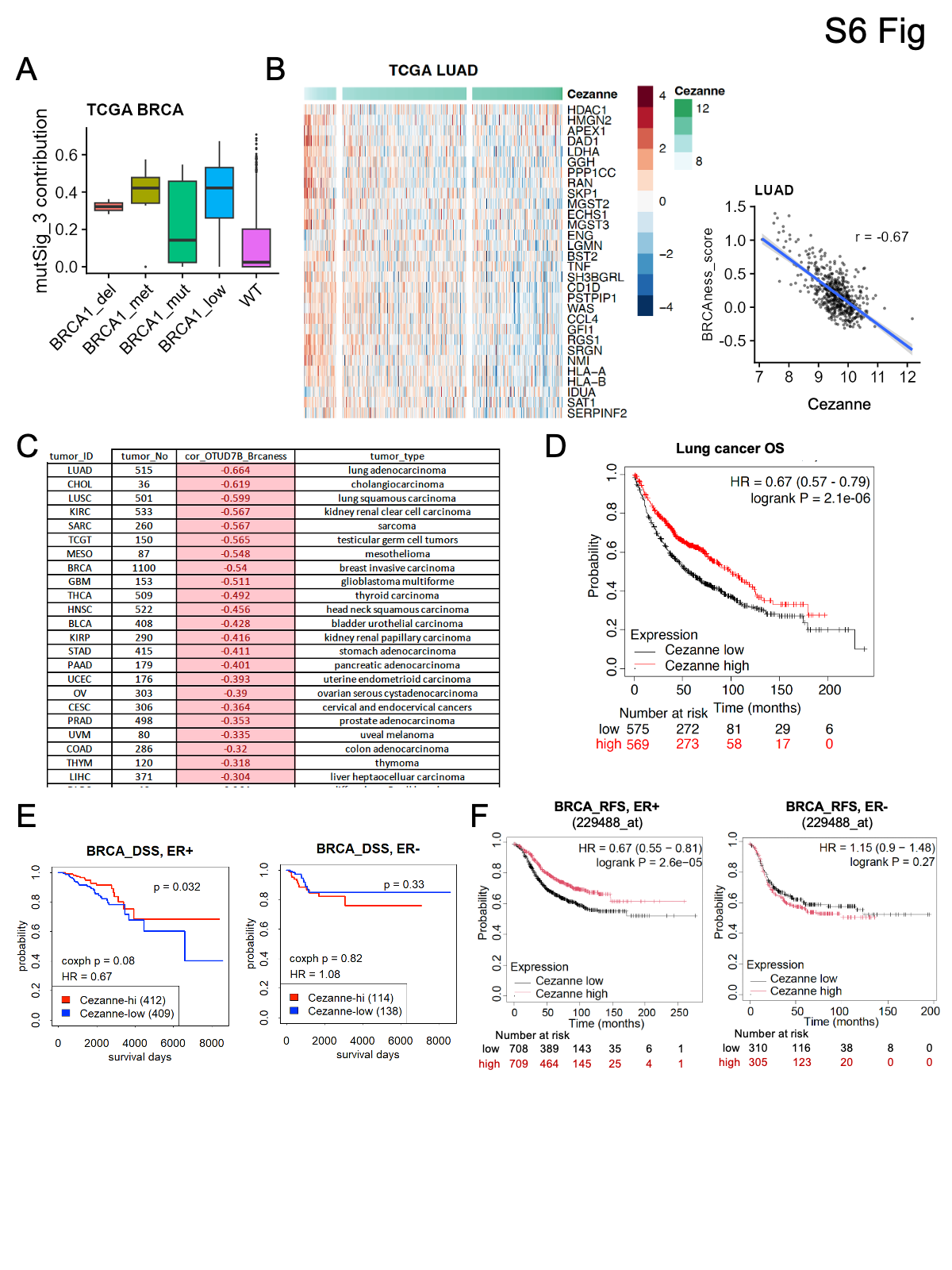

Supplement: S6 Fig — (A) Mutational signature 3 contribution in BRCA1 deficient breast cancer. TCGA BRCA tumors were grouped into different categories based on BRCA1 deletion, promoter methylation, mutation, and low expression status and compared to the remaining tumors (considered as BRCA1 wild type) for mutational signature 3 contribution. BRCA1 deletion and promoter methylation tumors were determined by TCGA copy number and DNA methylation data, respectively. BRCA1 mutation cases were determined using TCGA gene mutation data. A cutoff for BRCA1 low expression tumors was determined by the BRCA1 expression level in BRCA1 deletion and methylation cases. (B) Correlation of Cezanne expression to a 30-gene BRCAness signature in lung adenocarcinomas (LUAD). Left, heatmap of BRCAness signature genes in TCGA LUAD tumors ordered by Cezanne expression. Right, scatter plot of Cezanne expression against BRCAness signature scores. (C) Negative correlation of Cezanne expression with BRCAness signature in various tumors. Association R score is listed for various tumors. (D) Kaplan–Meier overall survival (OS) plot of lung cancer patients stratified by Cezanne expression level by median using the KM Plotter. Affymetrix ID229488_at was used for analyses. Data were from lung cancer patients with low (n = 575) and high (n = 569) Cezanne expression. Patient number at risk at different times of analyses is indicated at the bottom of the plots. Similar results were obtained with Affymetrix ID227436_at. (E) Kaplan-Meier curves showing disease specific survival (DSS) from TCGA BRCA patients separated into ER+ and ER- groups by K-means clustering (k = 2) of ESR1 gene expression. Within each ER group, patients were further stratified into high and low Cezanne expression using K-means clustering (k = 2). (F) Kaplan–Meier relapse free survival (RFS) plot of breast cancer patients stratified by Cezanne expression level by median and ER status by array using the KM Plotter. Affymetrix ID229488_at was used for analy [file pbio.3003545.s006.tiff]

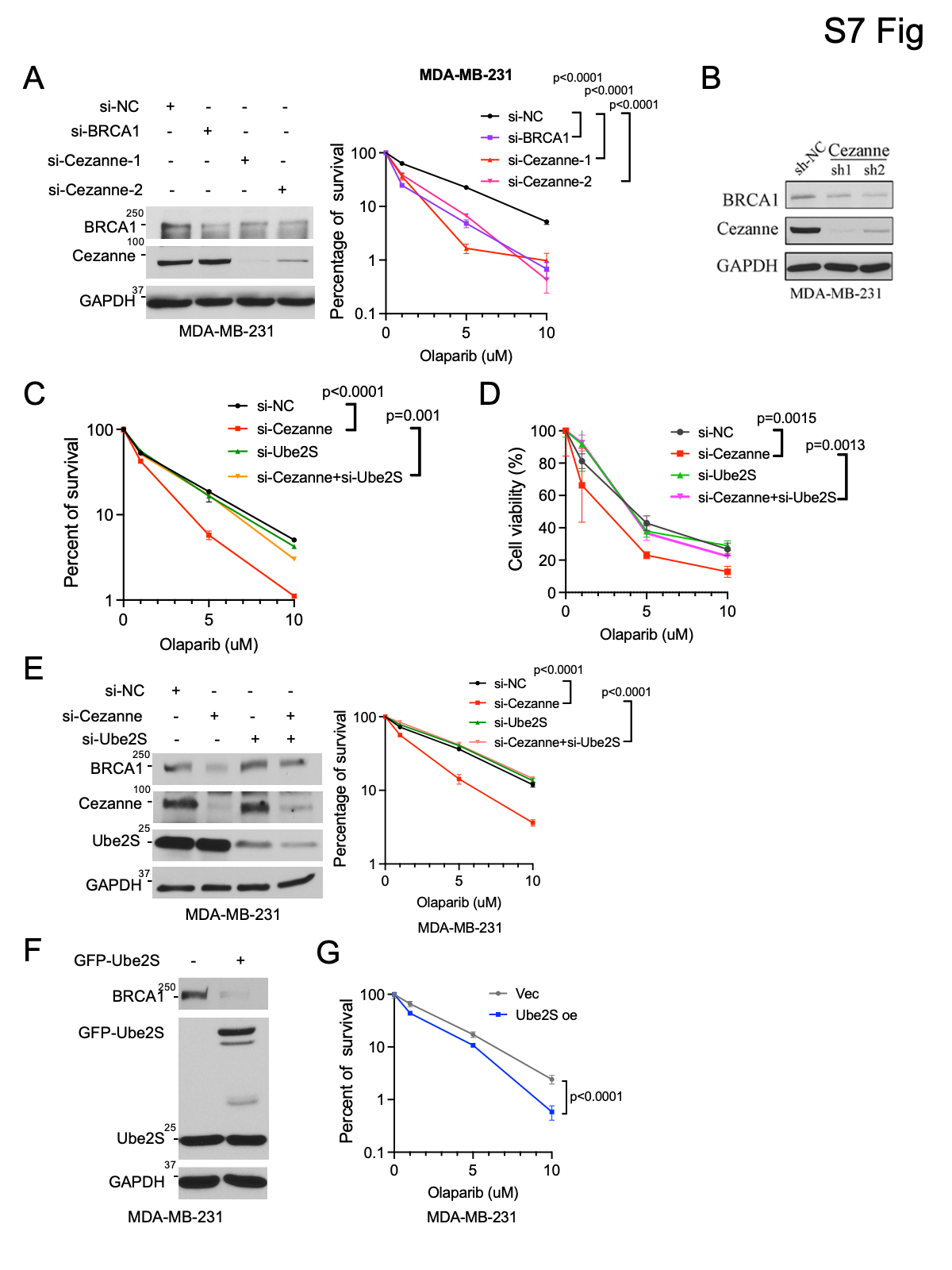

Supplement: S7 Fig — (A) Depletion of BRCA1 or Cezanne in MDA-MB-231 cells leads to increased cellular sensitivity to PARPi. Immunoblots confirming the effect of BRCA1 or Cezanne knockdown is shown (left panel). Colony survival assay was performed, and quantifications (n = 3) are shown with mean ± SD (right panel). Data is a representative of three independent experiments. (B) Knockdown of Cezanne in MDA-MB-231 cells used in mice xenograft experiments in Fig 5E. (C) Depletion of Ube2S rescues the increased cellular sensitivity of Cezanne-deficient U2OS cells to olaparib detected by colony formation assay. Quantifications (n = 3) are shown with mean ± SD. Data is a representative of three independent experiments. (D) Depletion of Ube2S rescues the increased cellular sensitivity of Cezanne-deficient cells to olaparib detected by cell viability assay 4-day after treatment with olaparib. Quantifications (n = 4) are shown with mean ± SD. Data is a representative of three independent experiments. (E) Depletion of Ube2S rescues the increased cellular sensitivity of Cezanne-deficient MDA-MB-231 cells to olaparib detected by colony formation assay. Immunoblots confirming the effect of the depletion of Cezanne or Ube2S and on BRCA1 protein level is shown (left panel). Quantifications (n = 3) are shown with mean ± SD. Data is a representative of three independent experiments. (F, G) Upregulation of Ube2S in MDA-MB-231 cells leads to reduced BRCA1 protein level and increased cellular sensitivity to PARPi. Immunoblots showing the effect of expression of GFP-Ube2S on BRCA1 protein level is shown in (F). The colony formation assay was performed and quantified (n = 3) with mean ± SD in (G). Data are representative of three independent experiments. Two-way Anova with Tukey’s multiple comparisons test was used for statistics in (A) (C) (D) (E). Ordinary Two-way Anova was used for statistics in (G). The data underlying the graphs shown in the figure can be found in S1 Data (TIFF) [file pbio.3003545.s007.tiff]

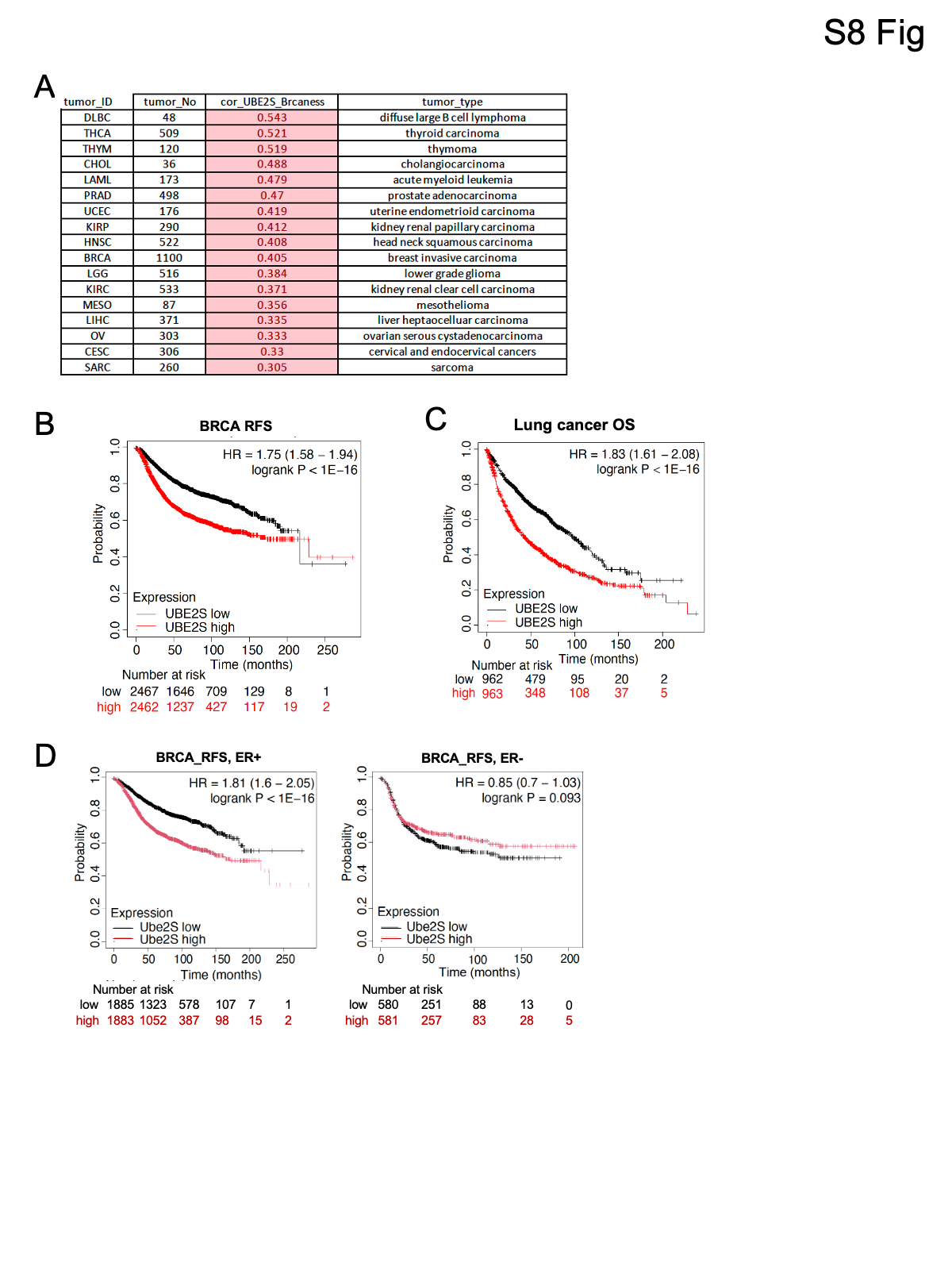

Supplement: S8 Fig — (A) Positive correlation of Ube2S expression with BRCAness signature in various tumors. Association R score is listed for various tumors. (B) Kaplan–Meier relapse free survival plot of breast cancer patients stratified by Ube2S expression level by median using the KM Plotter. Affymetrix ID202779_s_at was used for analyses. Data were from breast cancer patients with low (n = 2,467) and high (n = 2,462) Ube2S expression. Patient number at risk at different times of analyses is indicated at the bottom of the plots. (C) Kaplan–Meier overall survival plot of lung cancer patients stratified by Ube2S expression level by median using the KM Plotter. Affymetrix ID202779_s_at was used for analyses. Patient number at risk at different times of analyses is indicated at the bottom of the plots. (D) Kaplan–Meier relapse free survival (RFS) plot of breast cancer patients stratified by Cezanne expression level by median and ER status by array using the KM Plotter. Affymetrix ID202779_at was used for analyses. The data underlying the graphs shown in the figure can be found in S1 Data (TIFF) [file pbio.3003545.s008.tiff]
