# Supplementary material for: The deubiquitinating enzyme Cezanne stabilizes BRCA1 by counteracting APC/C and Ube2S-dependent Lys11-linked ubiquitination
Source: PLoS Biol. 2025 Dec 8;23(12):e3003545. doi: 10.1371/journal.pbio.3003545 (PMC12685207; doi:10.1371/journal.pbio.3003545)

Fig 1A

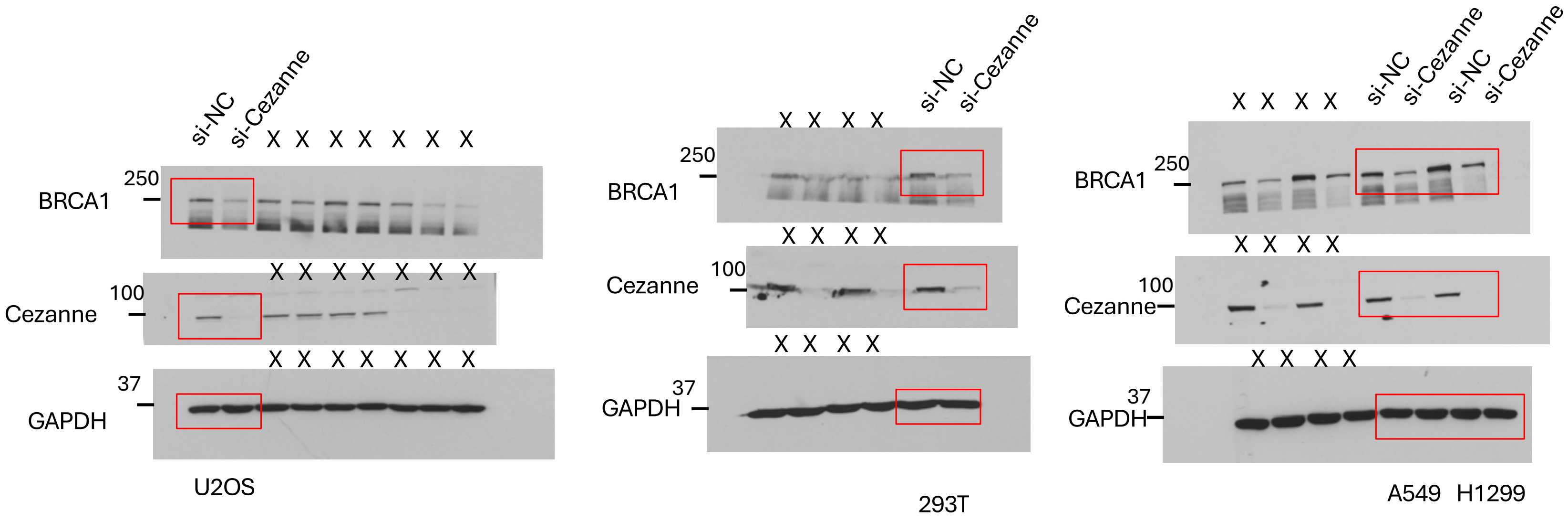

si-NC
si-Cezanne
X X X X X X X
BRCA1
250
Cezanne
100
GAPDH
37
U2OS
X X X X
si-NC
si-Cezanne
BRCA1
250
Cezanne
100
GAPDH
37
293T
X X X X
si-NC
si-Cezanne
si-NC
si-Cezanne
BRCA1
250
Cezanne
100
GAPDH
37
A549
H1299

Fig 1B

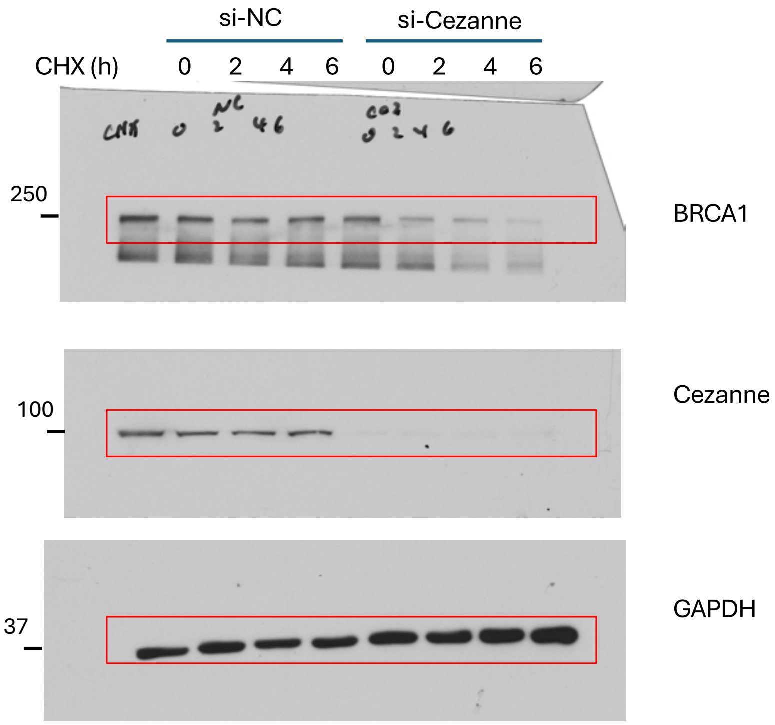

si-NC
si-Cezanne
CHX (h)
0 2 4 6 0 2 4 6
250
BRCA1
100
Cezanne
37
GAPDH

Fig 1C

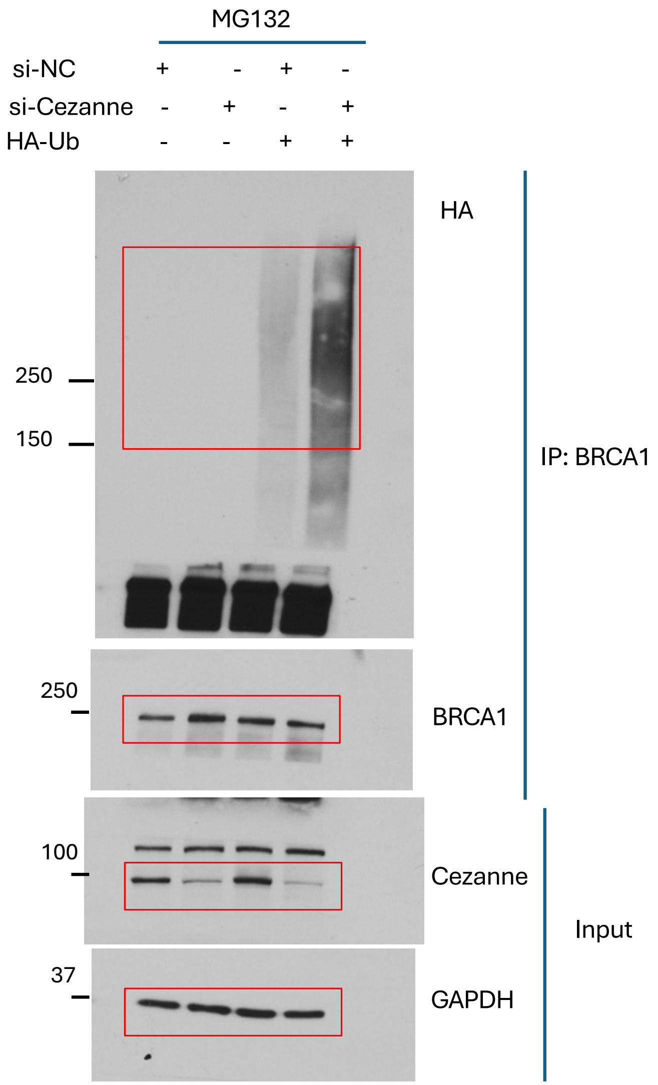
MG132
si-NC + - + -
si-Cezanne - + - +
HA-Ub - - + +
HA
250
150
IP: BRCA1
250
BRCA1
100
Cezanne
Input
37
GAPDH

Fig 1D

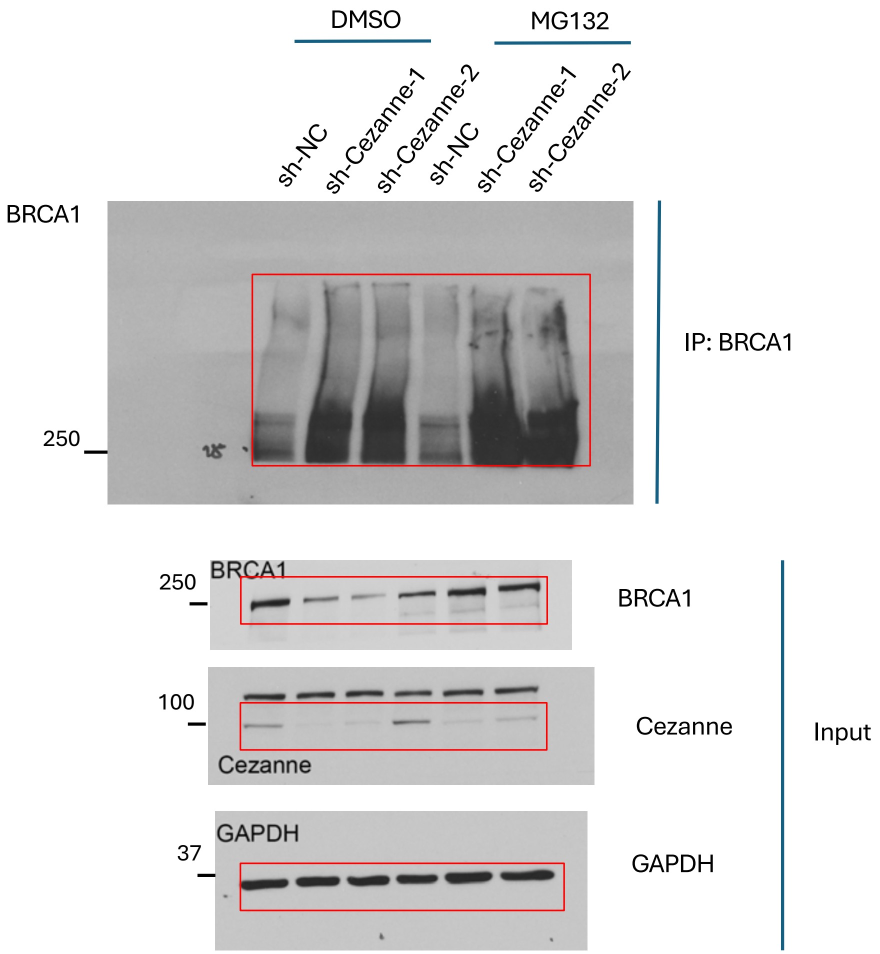
DMSO
MG132
sh-NC
sh-Cezanne-1
sh-Cezanne-2
sh-NC
sh-Cezanne-1
sh-Cezanne-2
BRCA1
250
IP: BRCA1
250
BRCA1
BRCA1
100
Cezanne
Cezanne
37
GAPDH
GAPDH
Input

Fig 1E

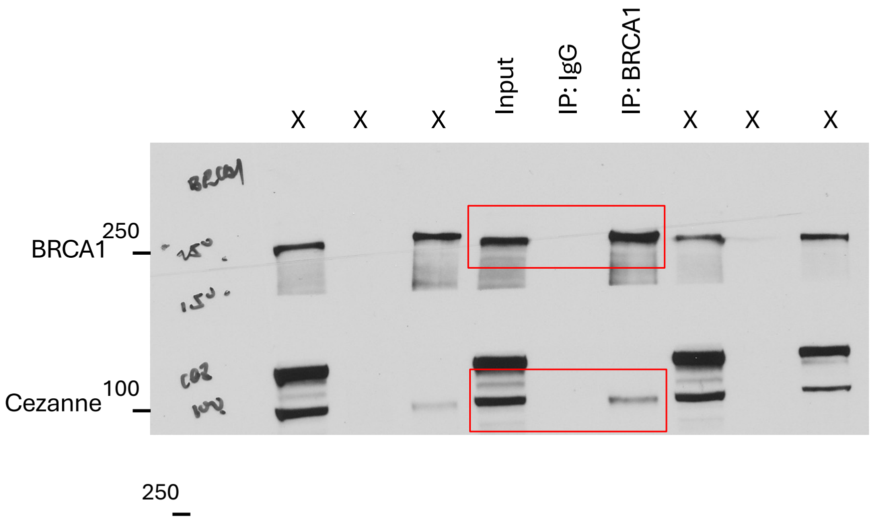
X
X
X
Input
IP: IgG
IP: BRCA1
X
X
X
BRCA1
250
Cezanne
100
250

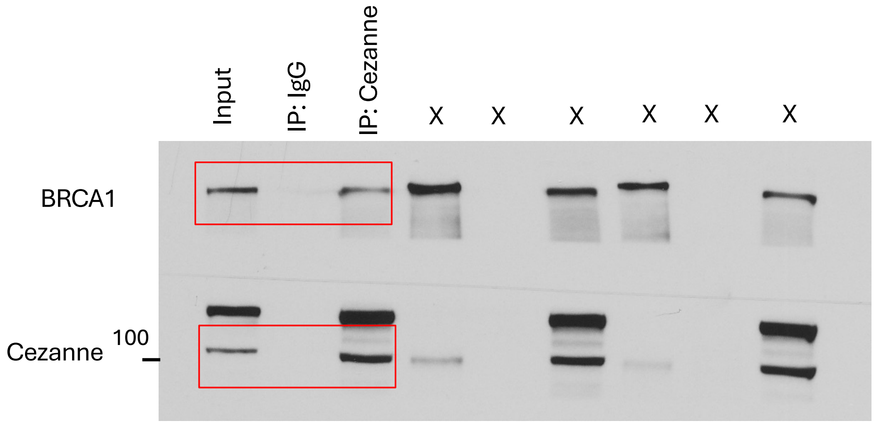
Input
IP: IgG
IP: Cezanne
X
X
X
X
X
X
BRCA1
Cezanne
100

Fig 2A

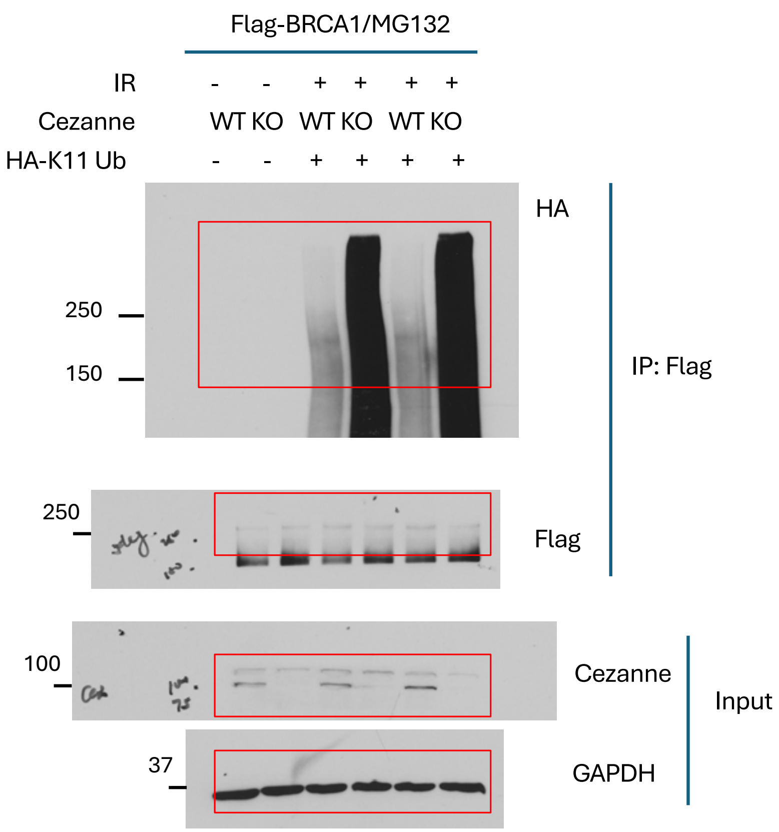
Flag-BRCA1/MG132
IR - - + + + +
Cezanne WT KO WT KO WT KO
HA-K11 Ub - - + + + +
HA
250
150
IP: Flag
250
Flag
100
Cezanne
Input
37
GAPDH

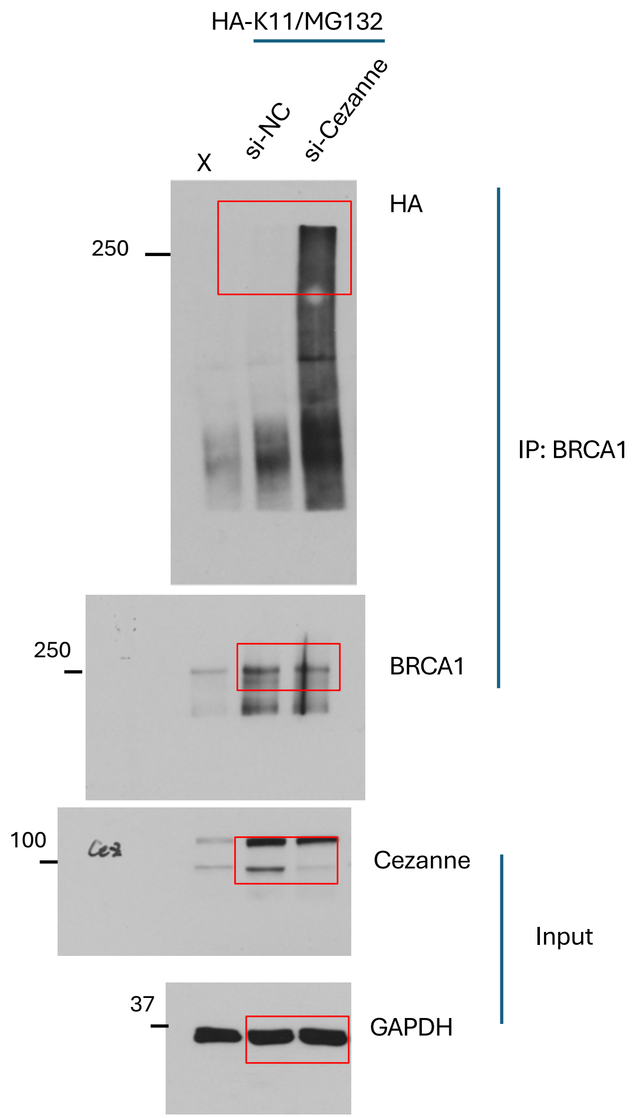
HA-K11/MG132
X
si-NC
si-Cezanne
250
HA
IP: BRCA1
250
BRCA1
100
Cez
Cezanne
Input
37
GAPDH

Fig 2B

Fig 2C

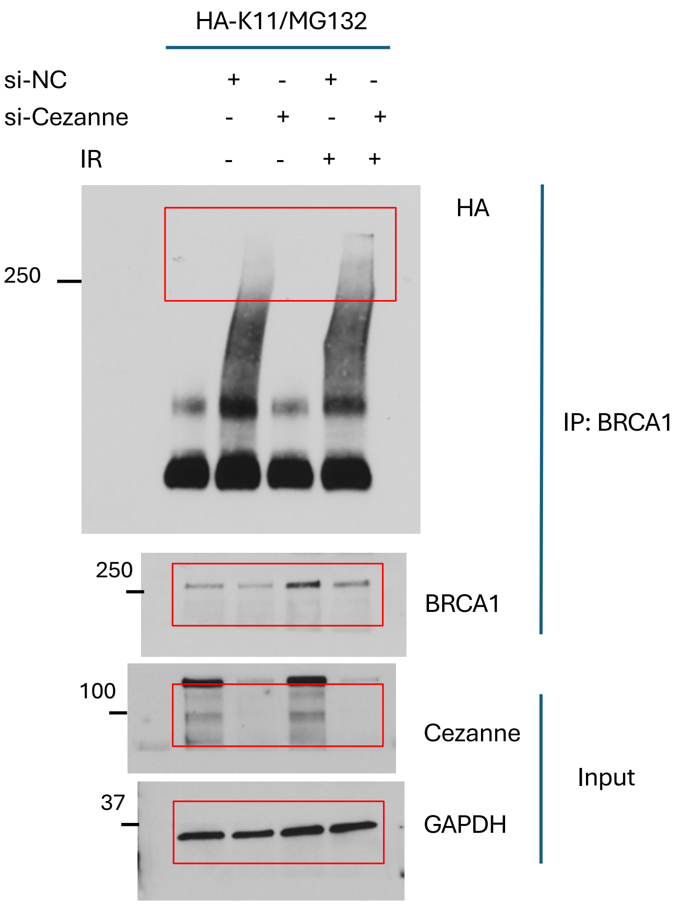

HA-K11/MG132
si-NC + - + -
si-Cezanne - + - +
IR - - + +
250
HA
IP: BRCA1
250
BRCA1
100
Cezanne
Input
37
GAPDH

Fig 2D

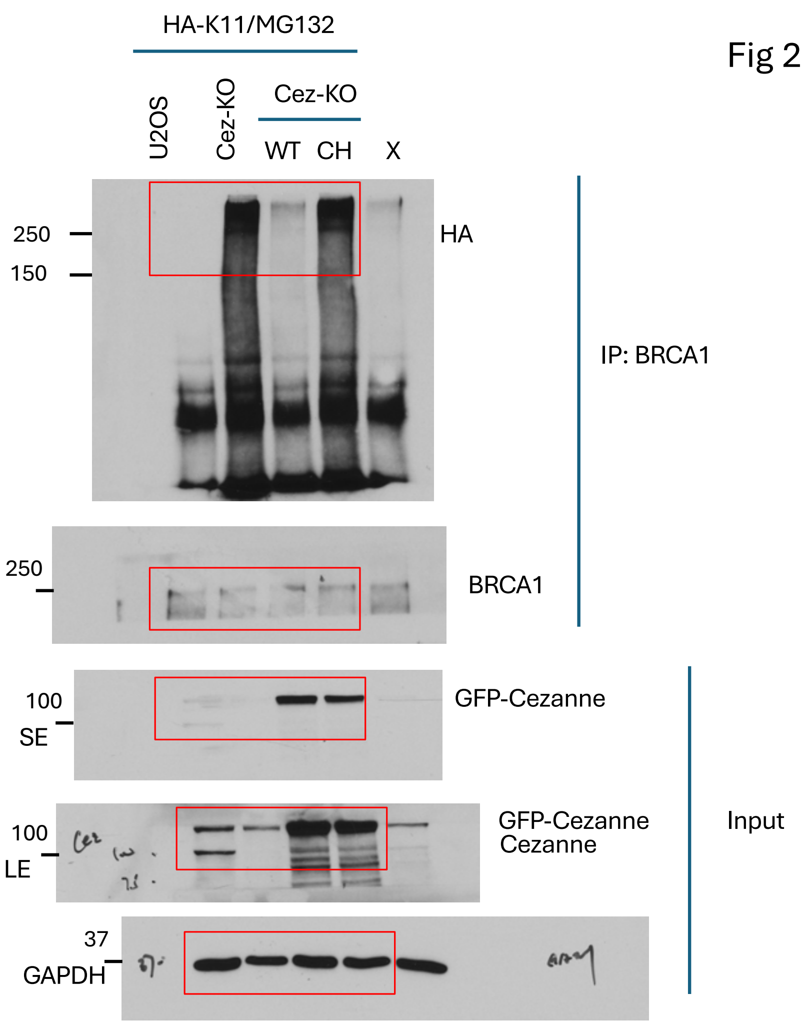
HA-K11/MG132
U2OS
Cez-KO
Cez-KO
WT CH
X
250
150
HA
IP: BRCA1
250
BRCA1
100
SE
GFP-Cezanne
100
LE
GFP-Cezanne
Cezanne
Input
37
GAPDH

Fig 2E

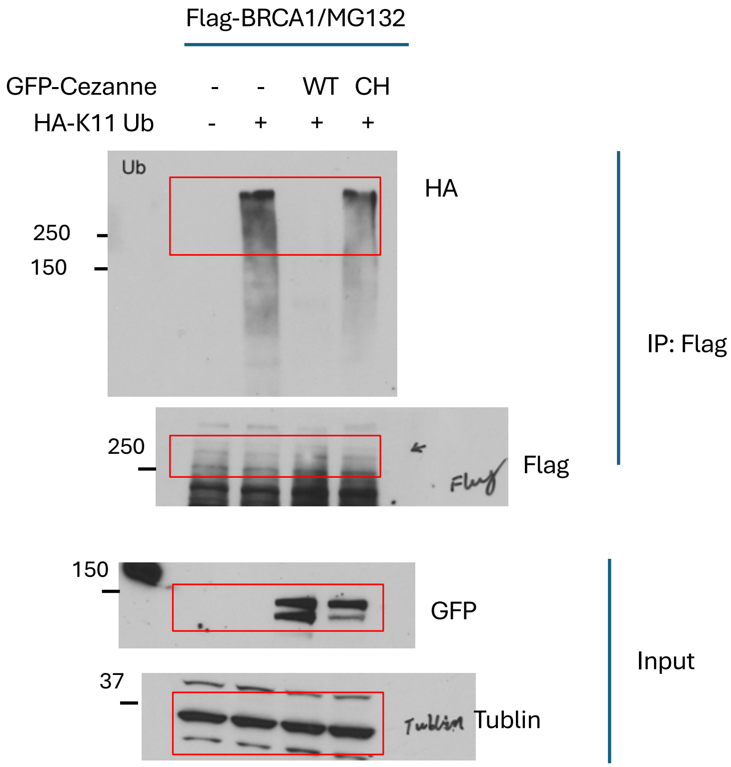
Flag-BRCA1/MG132
GFP-Cezanne - - WT CH
HA-K11 Ub - + + +
Ub
250
150
HA
250
Flag
IP: Flag
150
GFP
37
Tublin
Tublin
Input

Fig 2F

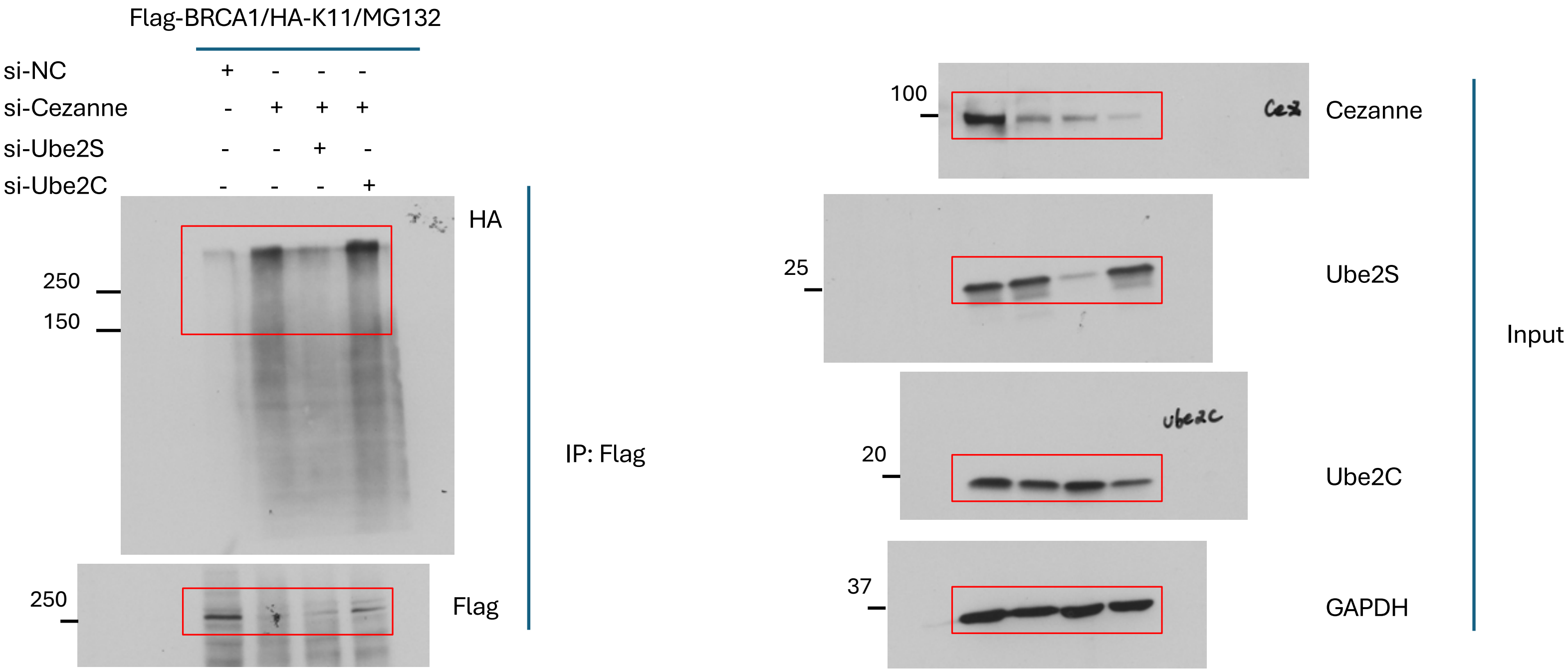
Flag-BRCA1/HA-K11/MG132
si-NC + - - -
si-Cezanne - + + +
si-Ube2S - - + -
si-Ube2C - - - +
HA
250
150
250
Flag
IP: Flag
100
Cezanne
25
Ube2S
20
Ube2C
37
GAPDH
Input

Fig 2G

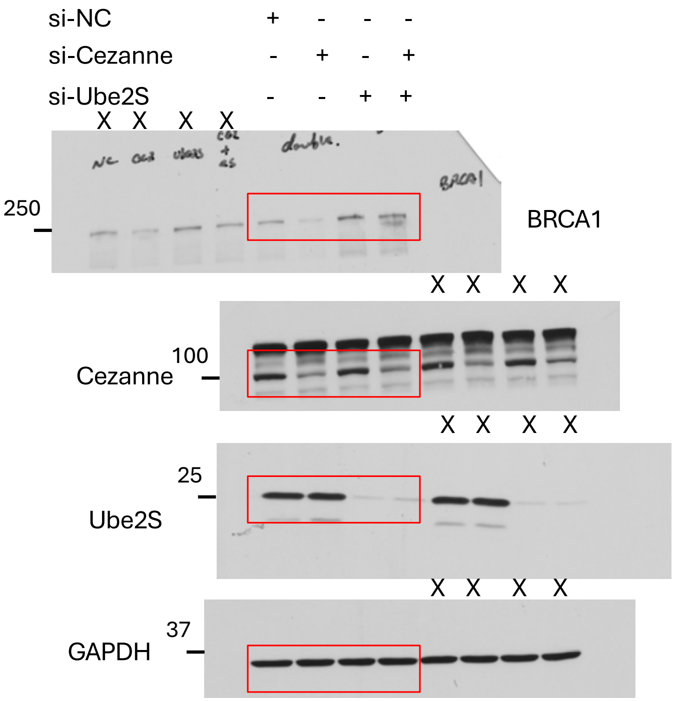

si-NC + - - -
si-Cezanne - + - +
si-Ube2S - - + +
250
BRCA1
Cezanne
100
25
Ube2S
GAPDH
37

Fig 3A

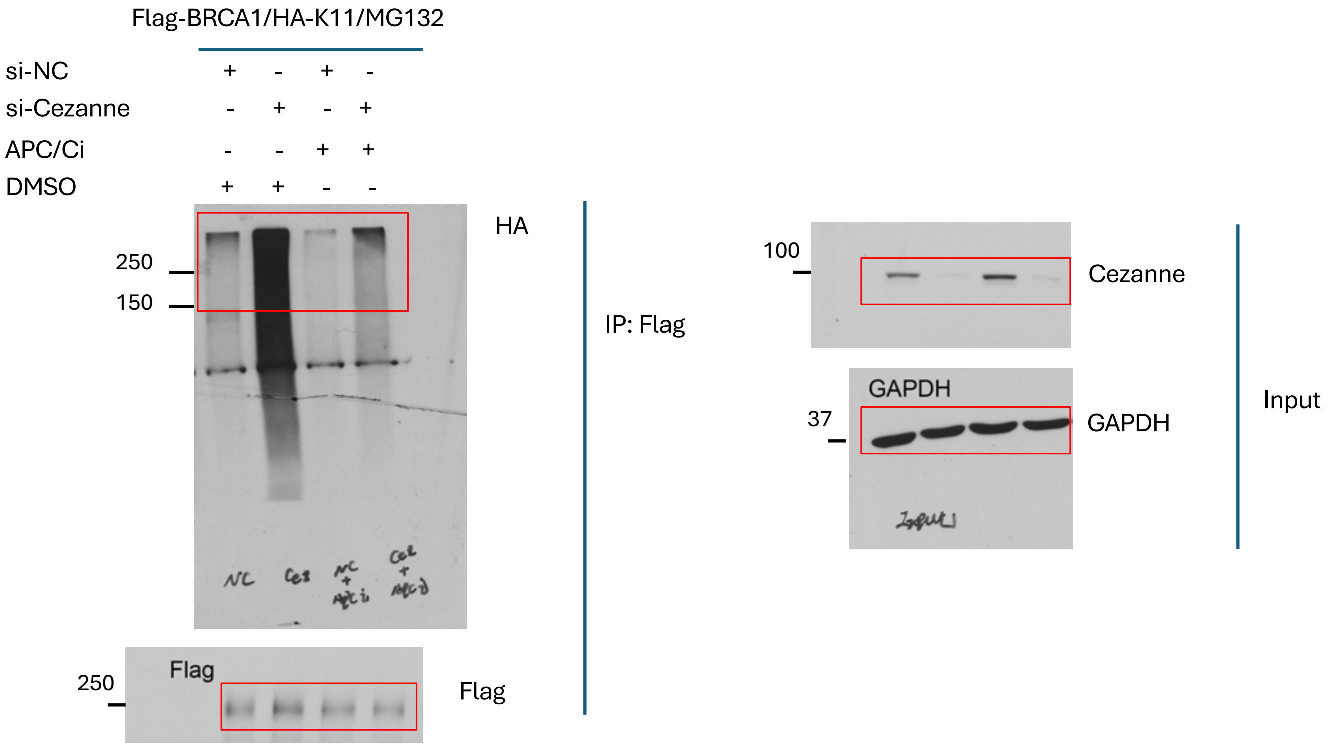
Flag-BRCA1/HA-K11/MG132
si-NC + - + -
si-Cezanne - + - +
APC/Ci - - + +
DMSO + + - -
250
150
HA
250
Flag
Flag
IP: Flag
100
Cezanne
GAPDH
37
GAPDH
Input

Fig 3B

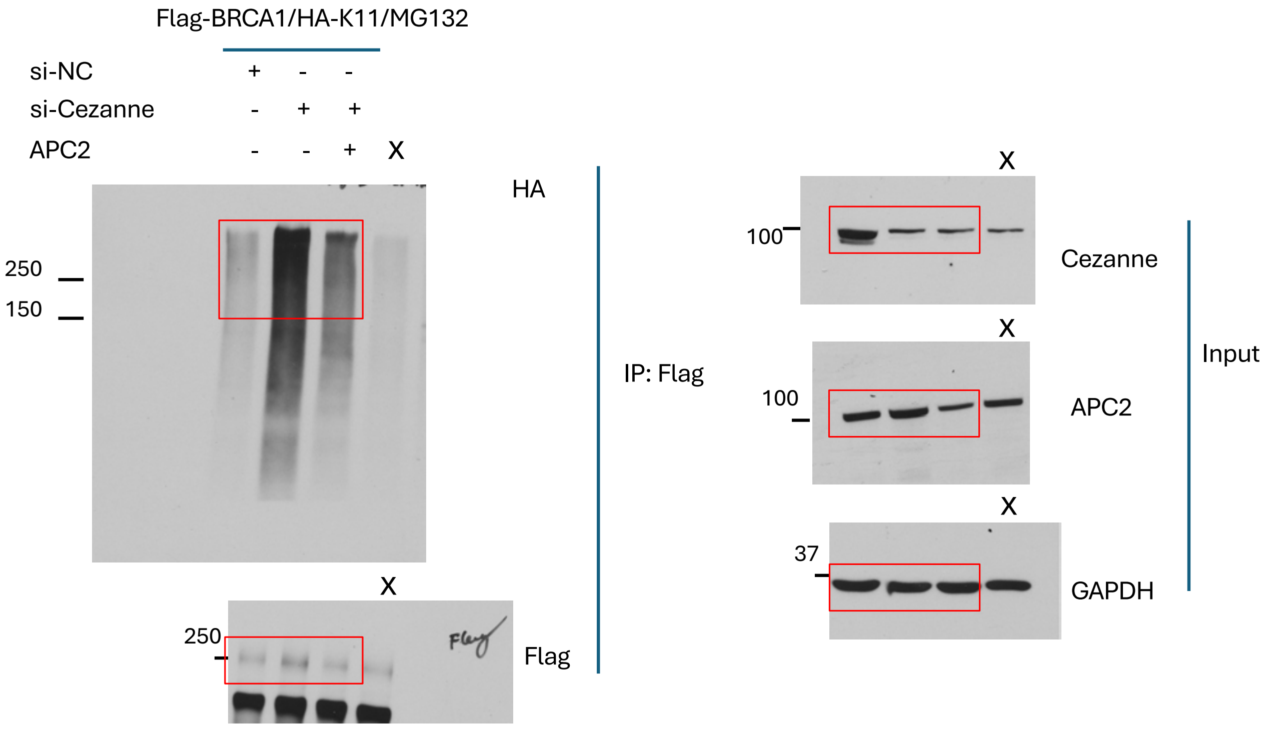
Flag-BRCA1/HA-K11/MG132
si-NC + - -
si-Cezanne - + +
APC2 - - + X
HA
250
150
X
250
Flag
IP: Flag
X
100
Cezanne
X
100
APC2
X
37
GAPDH
Input

Fig 3D

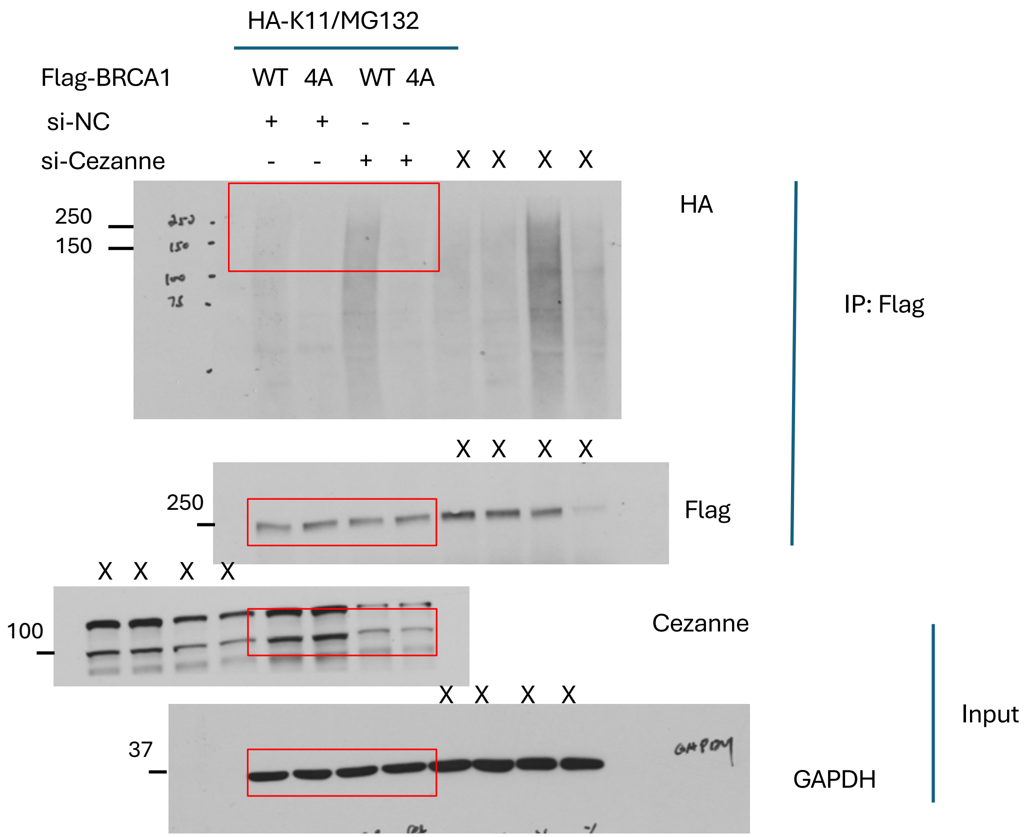
HA-K11/MG132
Flag-BRCA1
WT 4A WT 4A
si-NC
+ + - -
si-Cezanne
- - + +
X X X X
250
150
HA
IP: Flag
X X X X
250
Flag
X X X X
100
Cezanne
X X X X
Input
37
GAPDH

Fig 3E

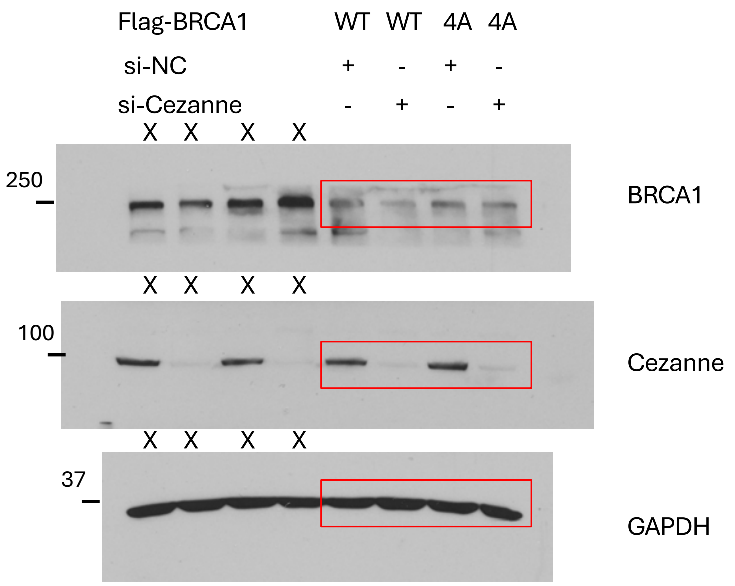

Flag-BRCA1 WT WT 4A 4A
si-NC + - + -
si-Cezanne - + - +
X X X X
250
BRCA1
X X X X
100
Cezanne
X X X X
37
GAPDH

Fig 3F

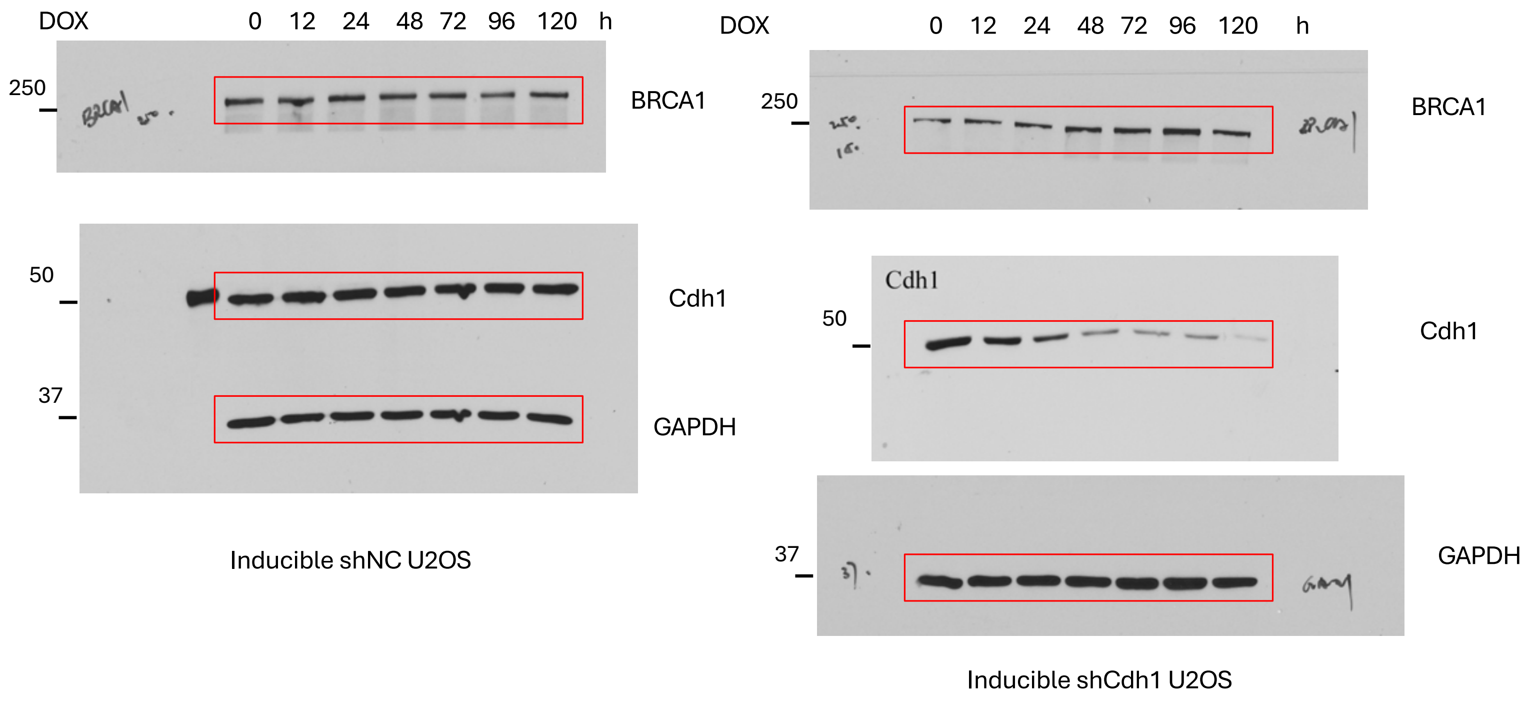
DOX
0
12
24
48
72
96
120
h
250
BRCA1
50
Cdh1
37
GAPDH
Inducible shNC U2OS
DOX
0
12
24
48
72
96
120
h
250
BRCA1
Cdh1
50
Cdh1
37
GAPDH
Inducible shCdh1 U2OS

Fig 3G

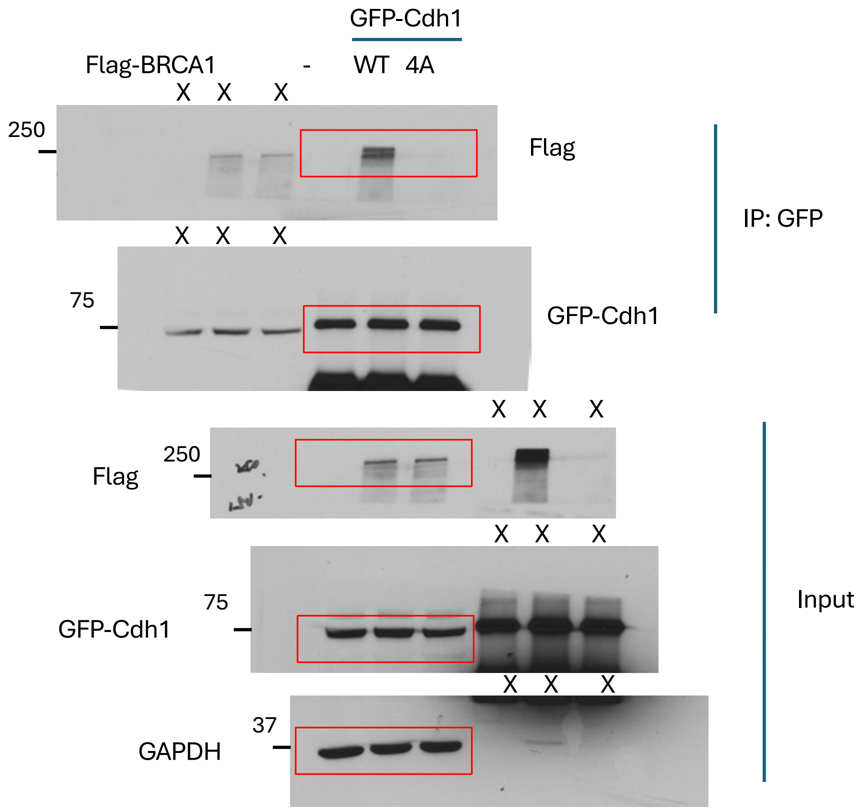
GFP-Cdh1
Flag-BRCA1
-
WT
4A
X X X
250
Flag
IP: GFP
X X X
75
GFP-Cdh1
X X X
Flag
250
X X X
75
GFP-Cdh1
Input
X X X
37
GAPDH

Fig 3H

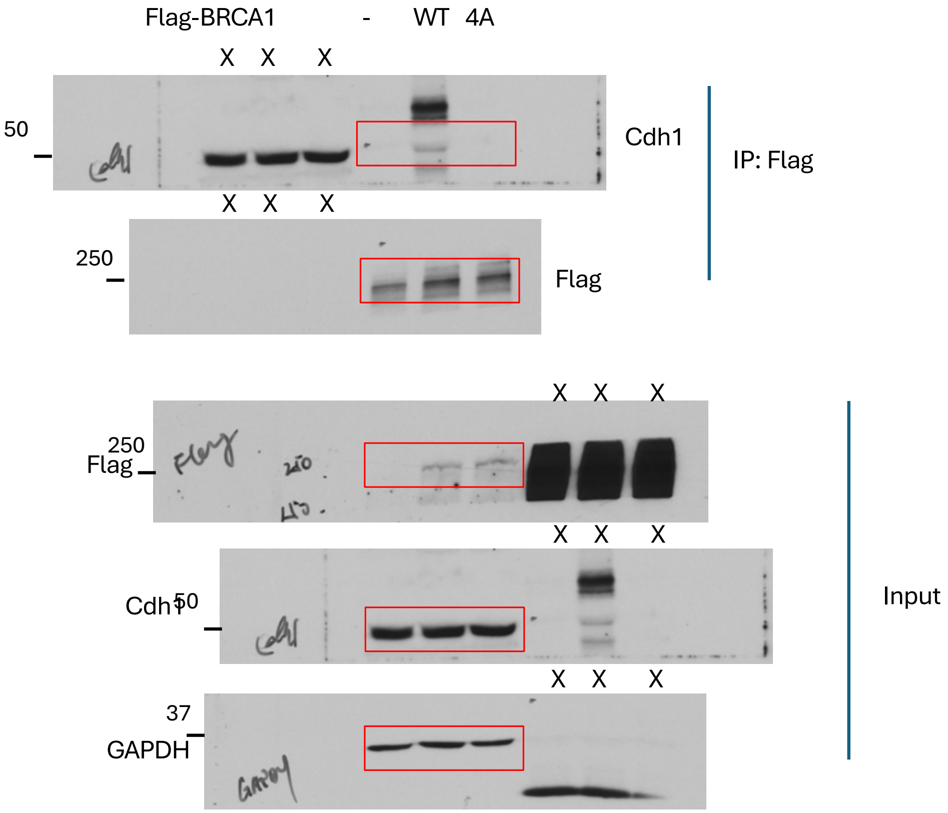
Flag-BRCA1
-
WT
4A
X X X
50
Cdh1
IP: Flag
X X X
250
Flag
X X X
250
Flag
X X X
Cdh1
50
X X X
37
GAPDH
Input

Fig 4A

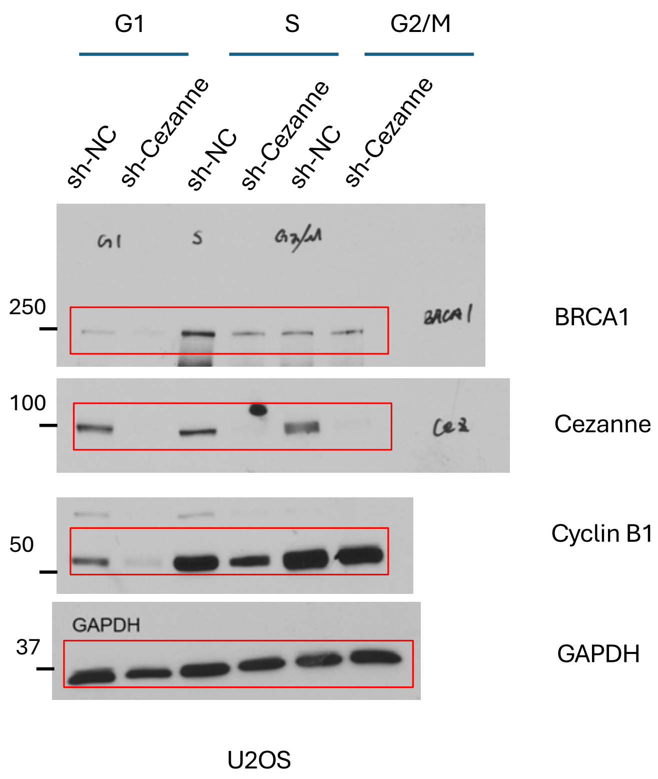
G1
S
G2/M
sh-NC
sh-Cezanne
sh-NC
sh-Cezanne
sh-NC
sh-Cezanne
250
BRCA1
100
Cezanne
50
Cyclin B1
GAPDH
37
GAPDH
U2OS

Fig 4B

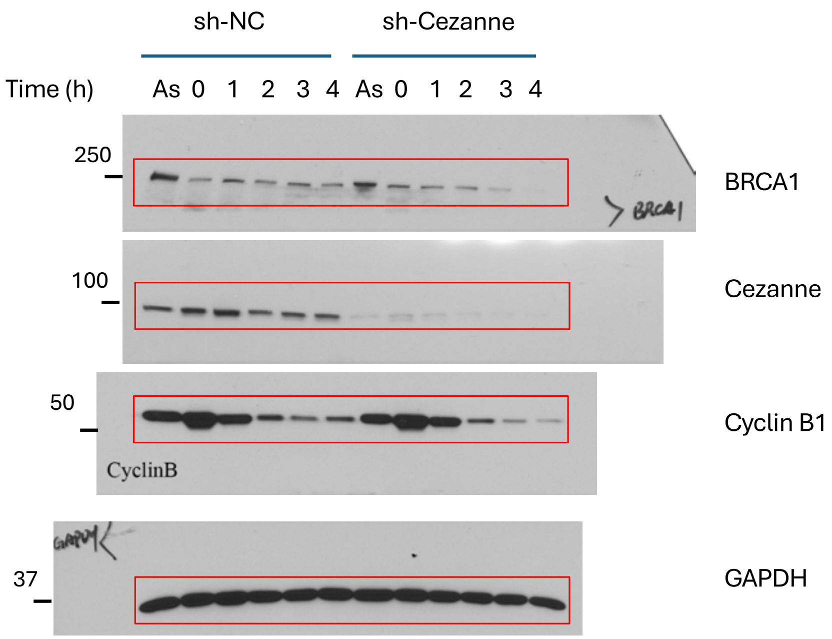

sh-NC
sh-Cezanne
Time (h)
As 0 1 2 3 4 As 0 1 2 3 4
250
BRCA1
100
Cezanne
50
CyclinB
Cyclin B1
37
GAPDH

U2OS

Fig 4C

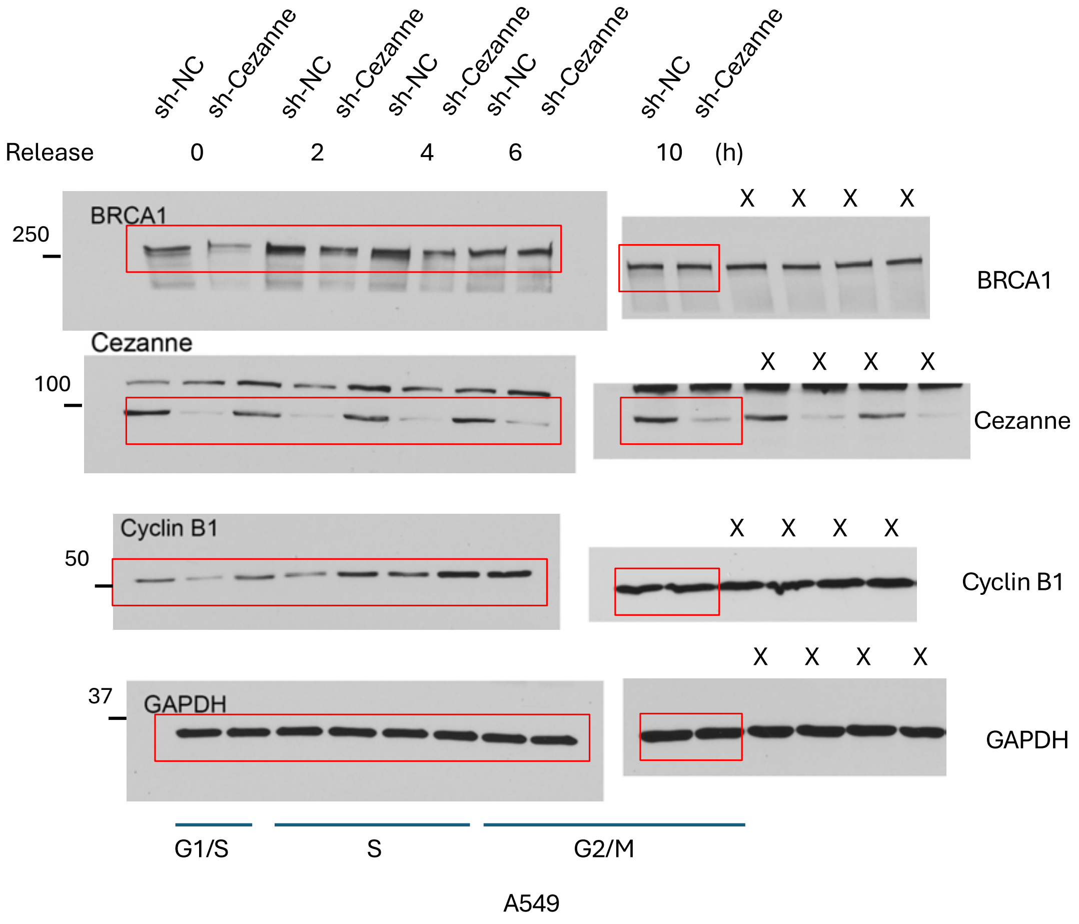

sh-NC
sh-Cezanne
sh-NC
sh-Cezanne
sh-NC
sh-Cezanne
sh-NC
sh-Cezanne
sh-NC
sh-Cezanne
Release
0
2
4
6
10
(h)
X X X X
BRCA1
250
BRCA1
Cezanne
100
X X X X
Cezanne
Cyclin B1
50
X X X X
Cyclin B1
37
GAPDH
X X X X
GAPDH
G1/S
S
G2/M
A549

Fig 4D

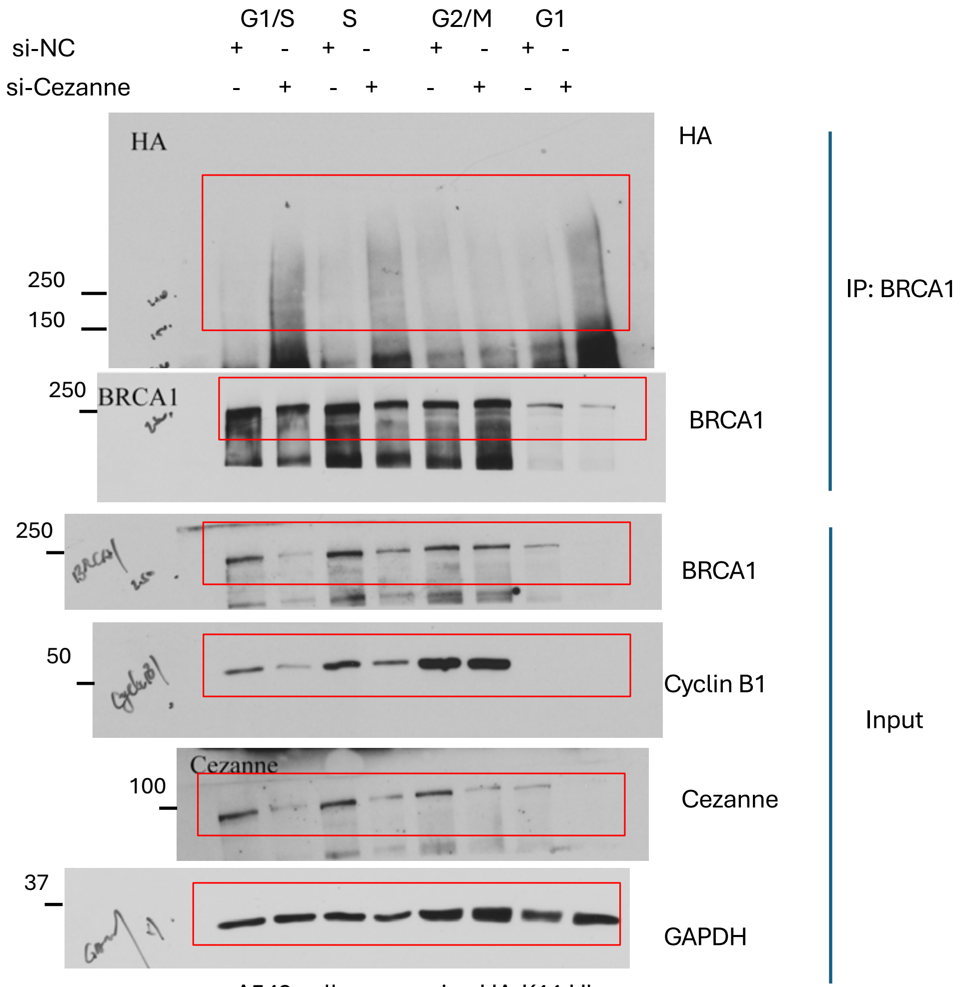

A549 cells expressing HA-K11 Ub

Fig 6A

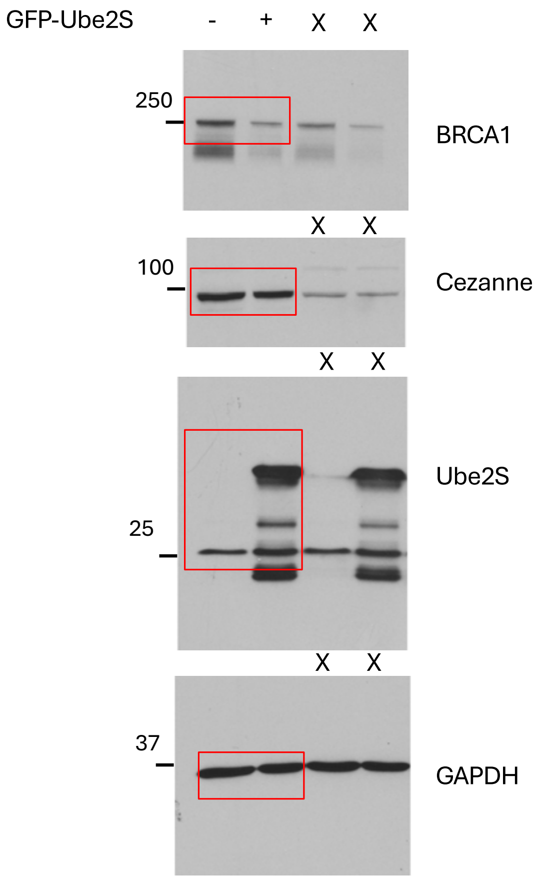

Fig S1A

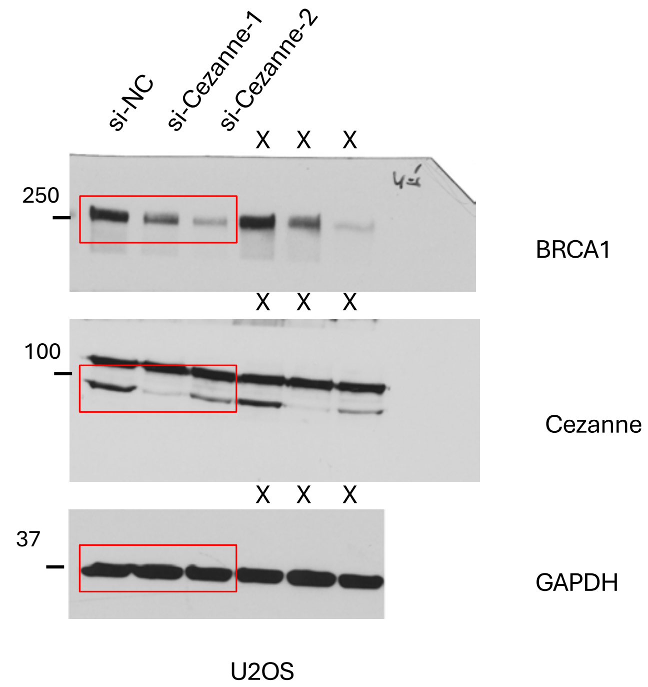

si-NC
si-Cezanne-1
si-Cezanne-2
X X X
250
BRCA1
X X X
100
Cezanne
X X X
37
GAPDH
U2OS

Fig S1B

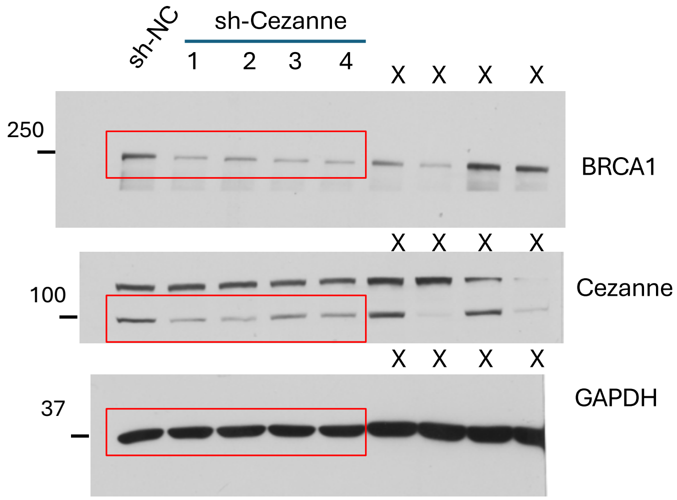

sh-NC
sh-Cezanne
1
2
3
4
X X X X
250
BRCA1
X X X X
100
Cezanne
X X X X
37
GAPDH

Fig S1C

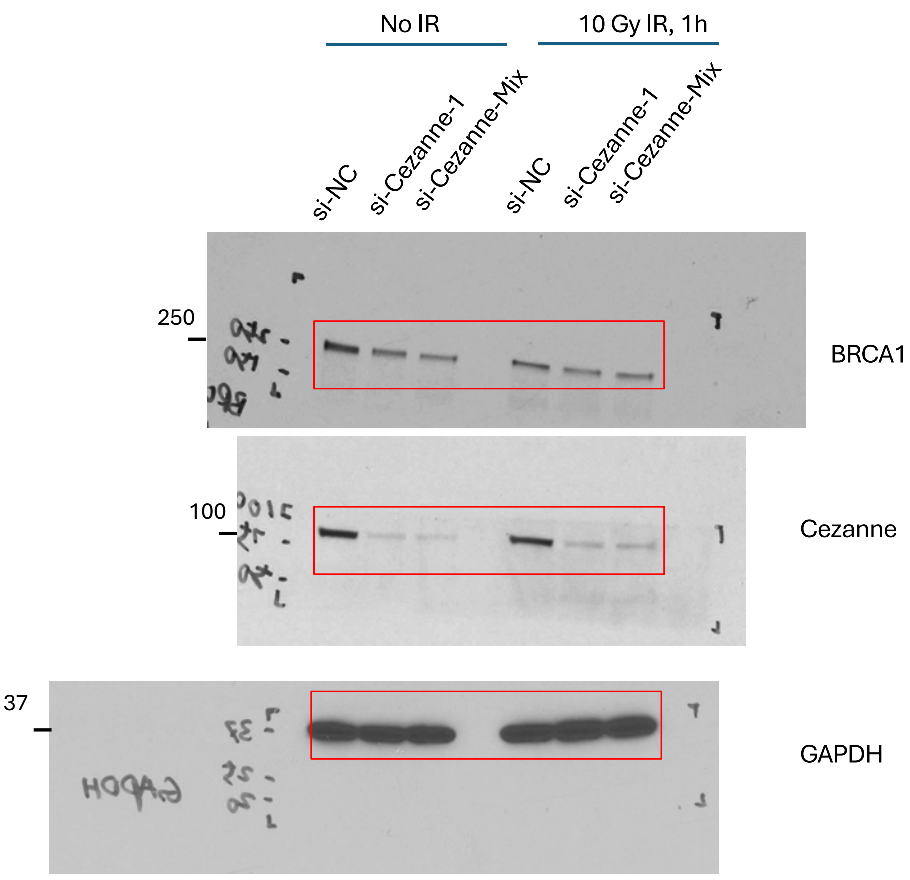
No IR
10 Gy IR, 1h
si-NC
si-Cezanne-1
si-Cezanne-Mix
si-NC
si-Cezanne-1
si-Cezanne-Mix
250
BRCA1
100
Cezanne
37
GAPDH

U2OS

Fig S1D

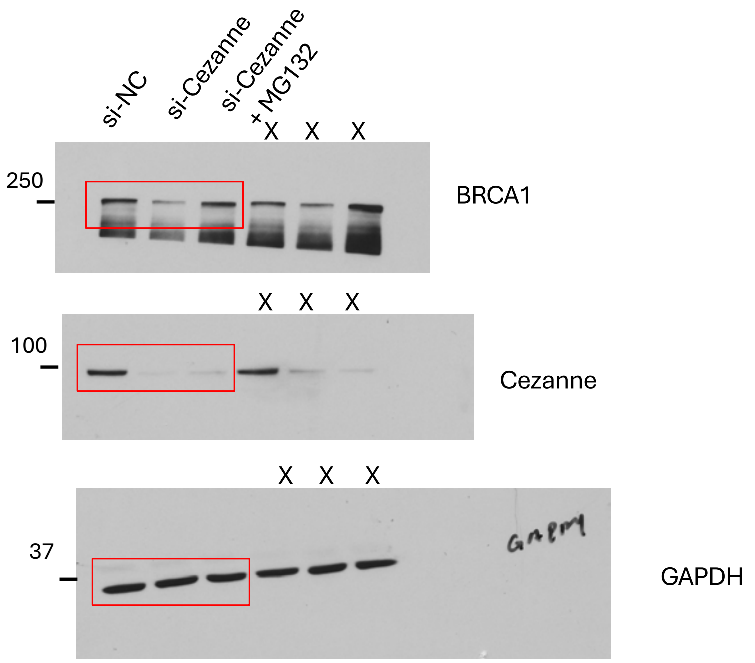
si-NC
si-Cezanne
si-Cezanne
+ MG132
X X X
250
BRCA1
X X X
100
Cezanne
X X X
37
GAPDH

Fig S1F

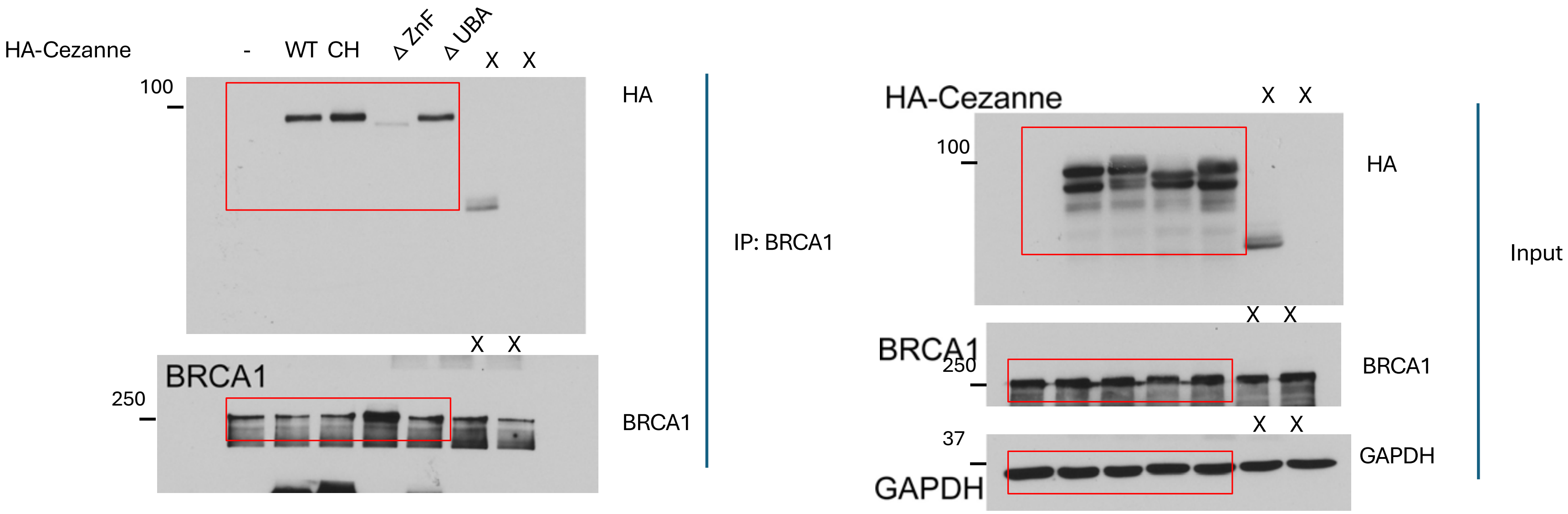

HA-Cezanne
-
WT
CH
Δ ZnF
Δ UBA
X
X
100
HA
BRCA1
250
BRCA1
IP: BRCA1
HA-Cezanne
100
HA
BRCA1
250
BRCA1
37
GAPDH
GAPDH
Input

Fig S2A

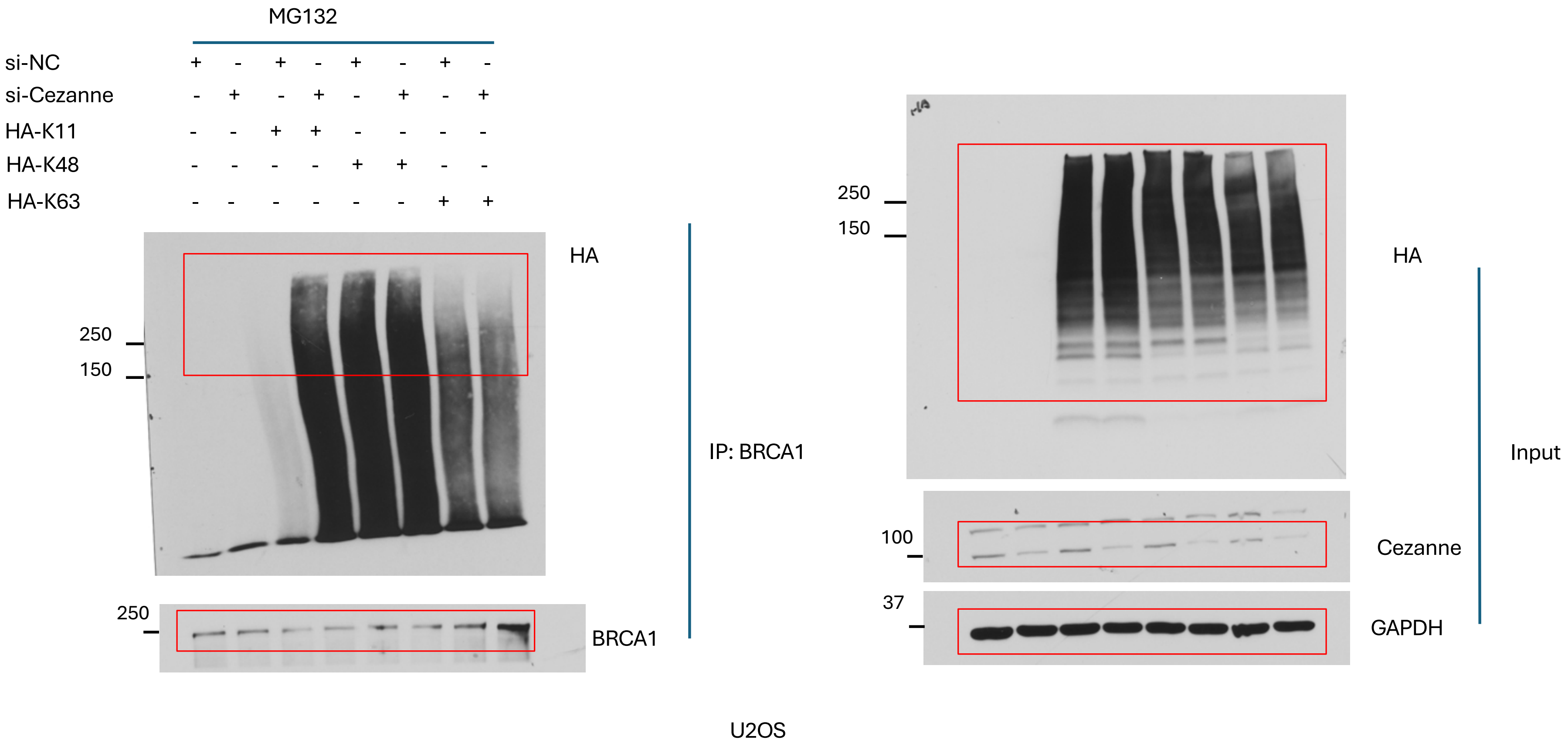

MG132
si-NC + - + - + - + -
si-Cezanne - + - + - + - +
HA-K11 - - + + - - - -
HA-K48 - - - - + + - -
HA-K63 - - - - - - + +
250
150
HA
250
BRCA1
IP: BRCA1
250
150
HA
100
Cezanne
37
GAPDH
Input
U2OS

Fig S2B

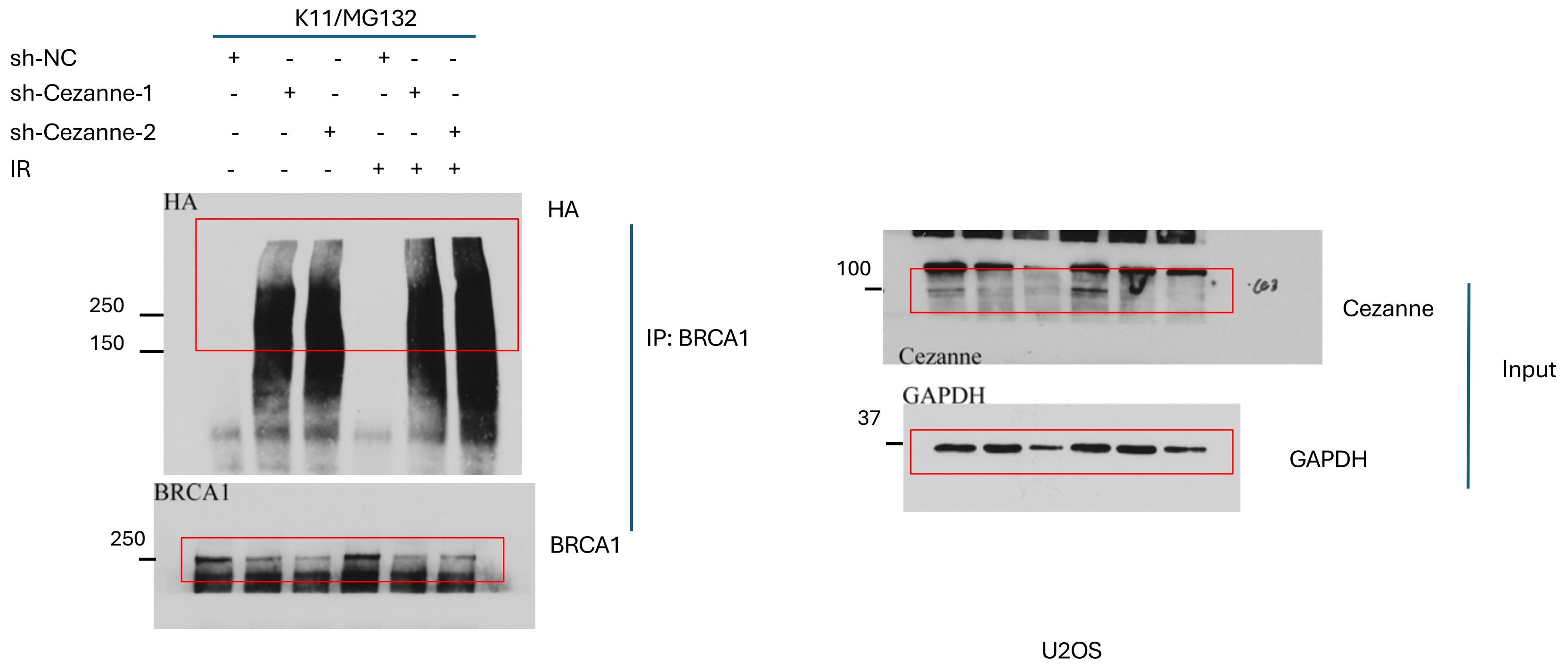
K11/MG132
sh-NC + - - + - -
sh-Cezanne-1 - + - - + -
sh-Cezanne-2 - - + - - +
IR - - - + + +
HA
250
150
HA
IP: BRCA1
BRCA1
250
BRCA1
100
Cezanne
Cezanne
Input
GAPDH
37
GAPDH
U2OS

Fig S2C

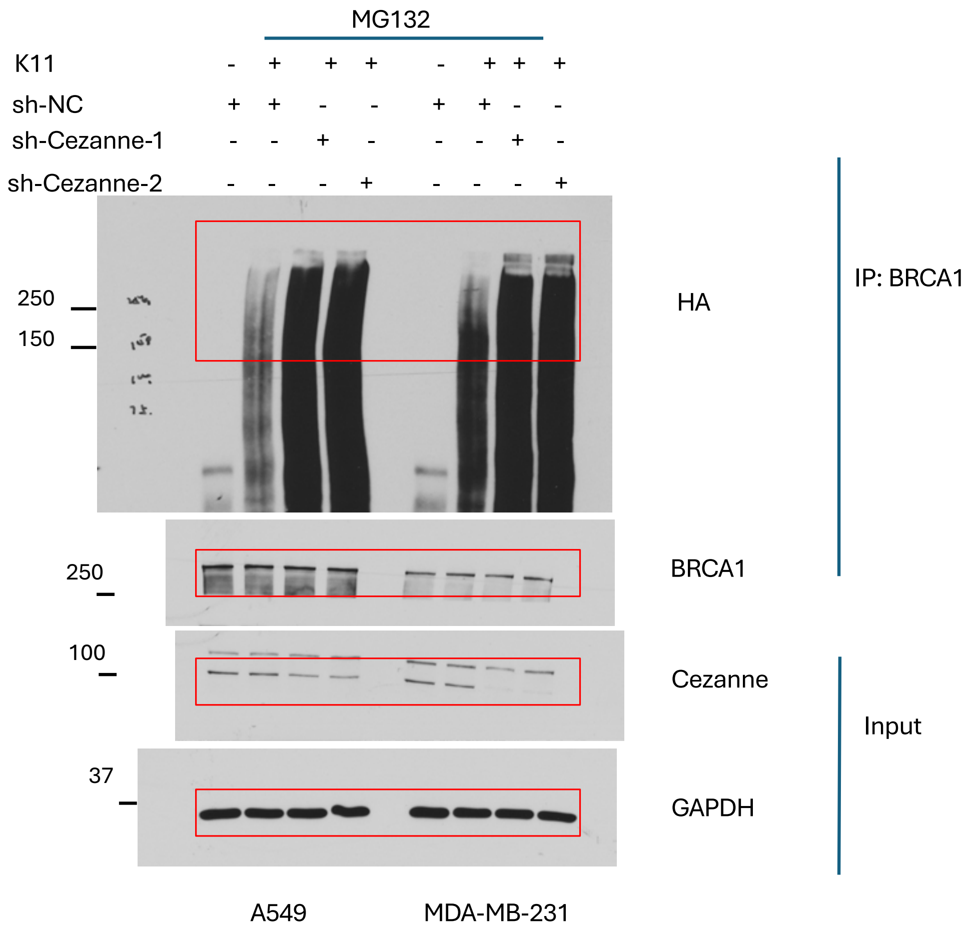
MG132
K11
sh-NC
sh-Cezanne-1
sh-Cezanne-2
250
150
HA
IP: BRCA1
250
BRCA1
100
Cezanne
Input
37
GAPDH
A549
MDA-MB-231

Fig S2D

Marker

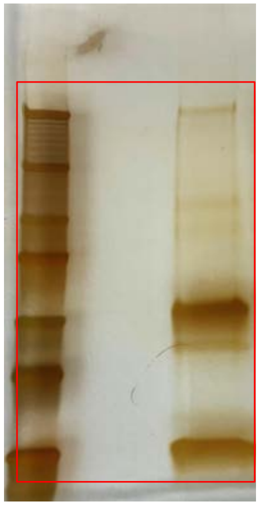

Purified Flag-BRCA1 on beads

Fig S2E

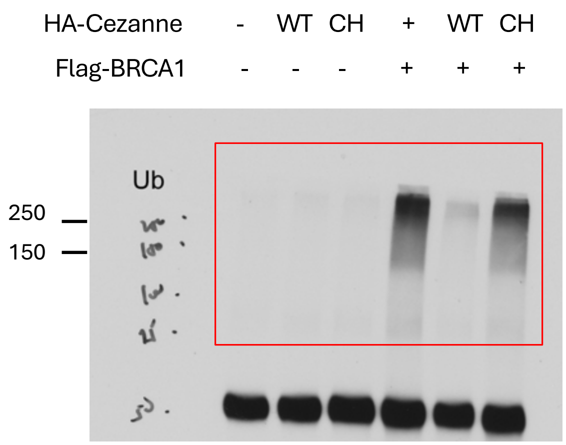

Fig S2F

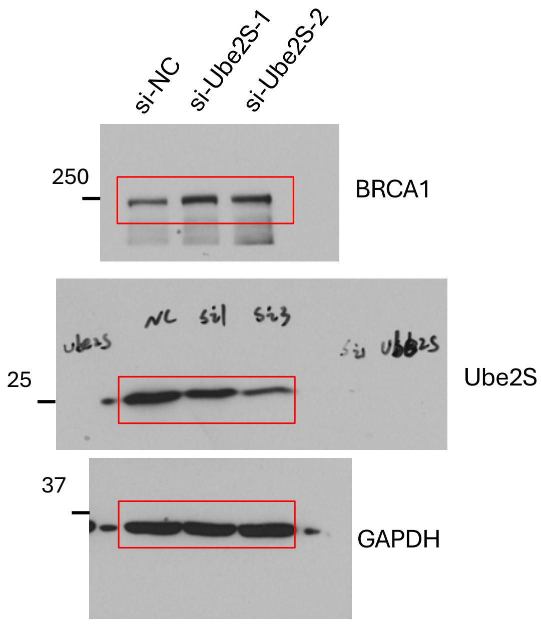

U2OS

Fig S3A

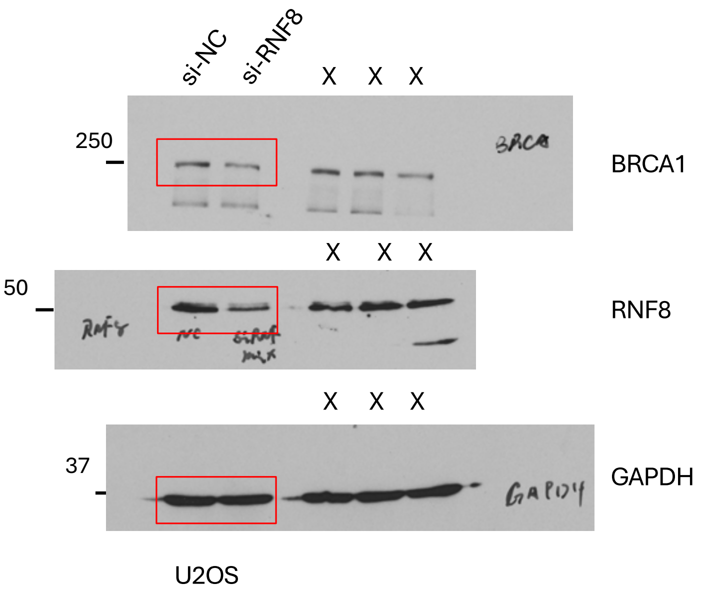

Fig S3B

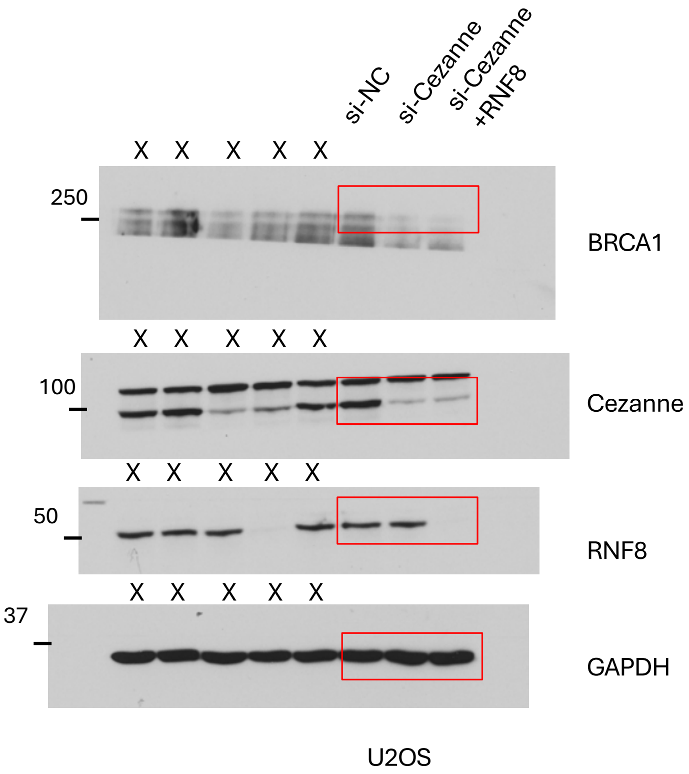

si-NC
si-Cezanne
si-Cezanne +RNF8
X X X X X
250
BRCA1
X X X X X
100
Cezanne
X X X X X
50
RNF8
X X X X X
37
GAPDH
U2OS

Fig S3C

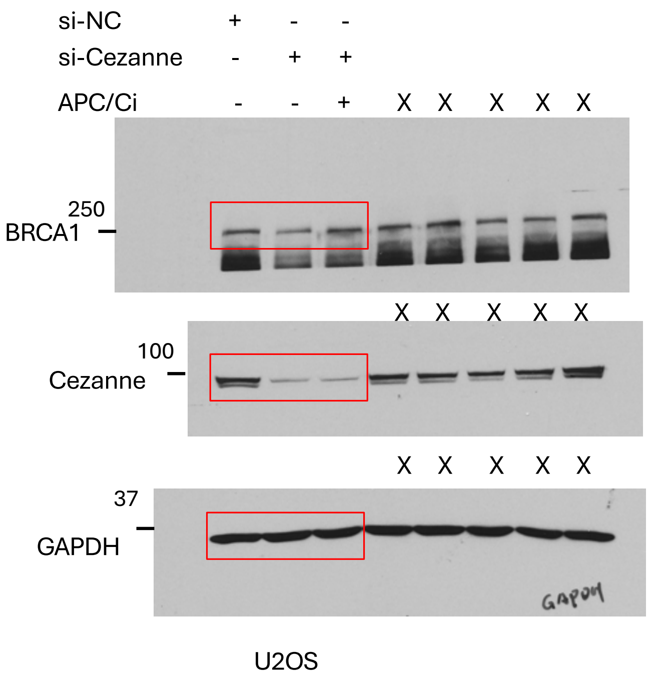
si-NC + - -
si-Cezanne - + +
APC/Ci - - +
X X X X X
250
BRCA1
X X X X X
100
Cezanne
X X X X X
37
GAPDH
U2OS

Fig S3D

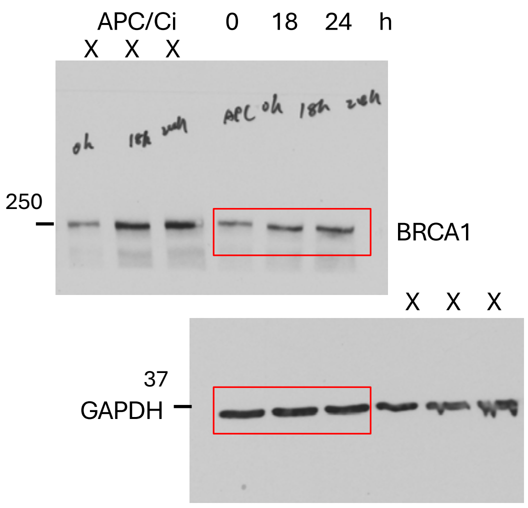

APC/Ci
0
18
24
h
X X X
oh
18h
24h
APC oh
18h
24h
250
BRCA1
X X X
37
GAPDH

U2OS

Fig S3E

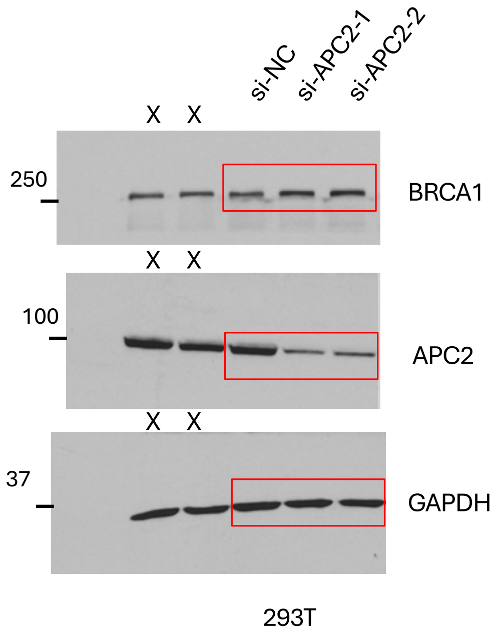

si-NC
si-APC2-1
si-APC2-2
X X
250
BRCA1
X X
100
APC2
X X
37
GAPDH
293T

Fig S3G

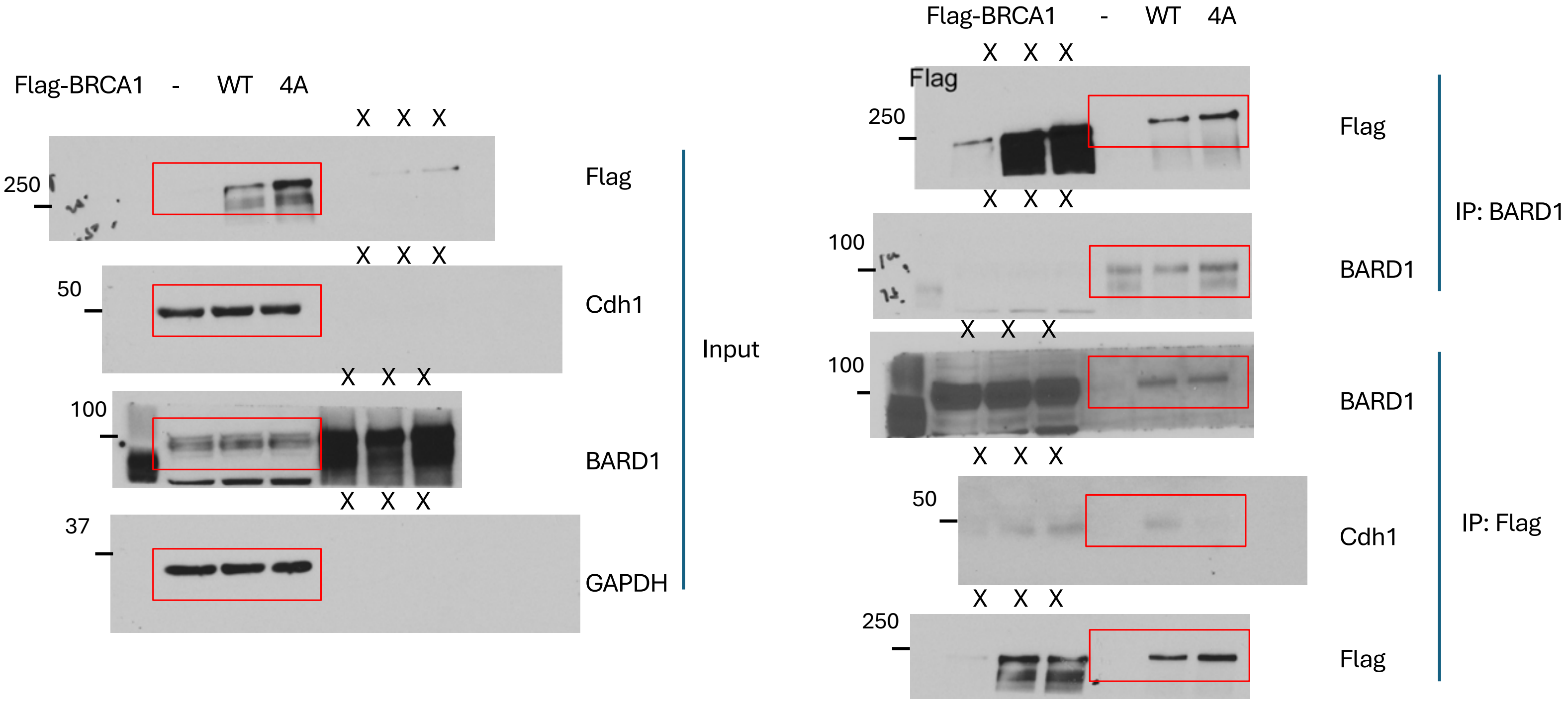

Flag-BRCA1 - WT 4A
X X X
250
Flag
50
Cdh1
100
BARD1
37
GAPDH
Input
Flag-BRCA1 - WT 4A
Flag
250
Flag
100
BARD1
IP: BARD1
100
BARD1
50
Cdh1
IP: Flag
250
Flag

293T

Fig S3H

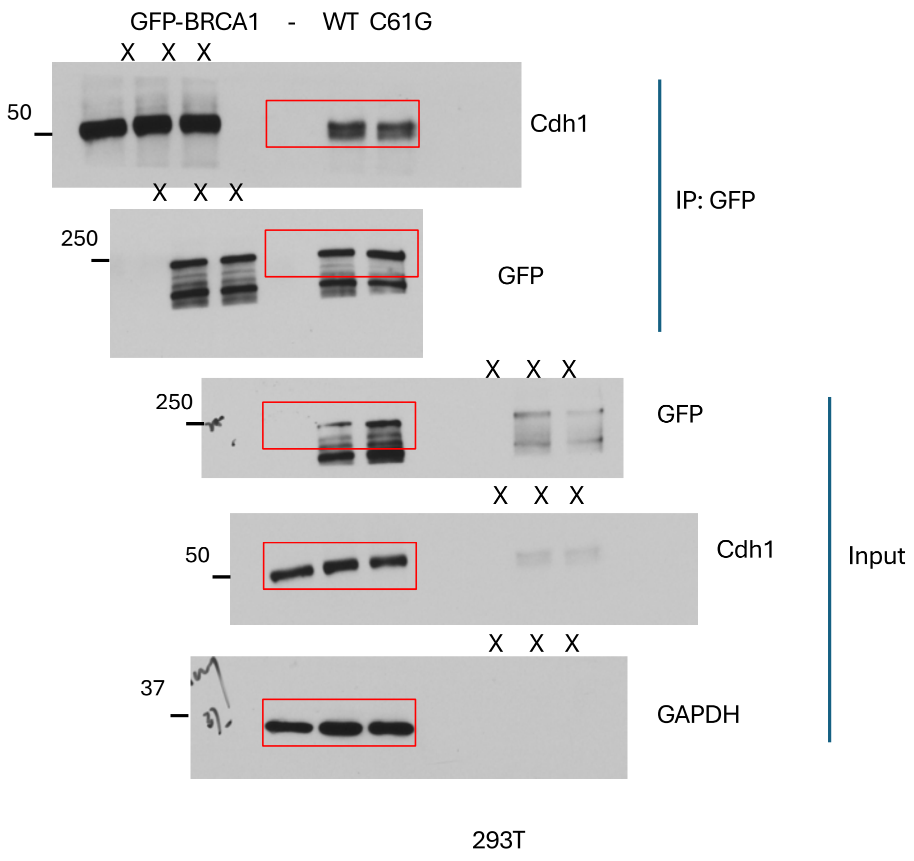
GFP-BRCA1
-
WT
C61G
X X X
50
Cdh1
X X X
250
GFP
IP: GFP
X X X
250
GFP
X X X
50
Cdh1
Input
X X X
37
GAPDH
293T

Fig S4C

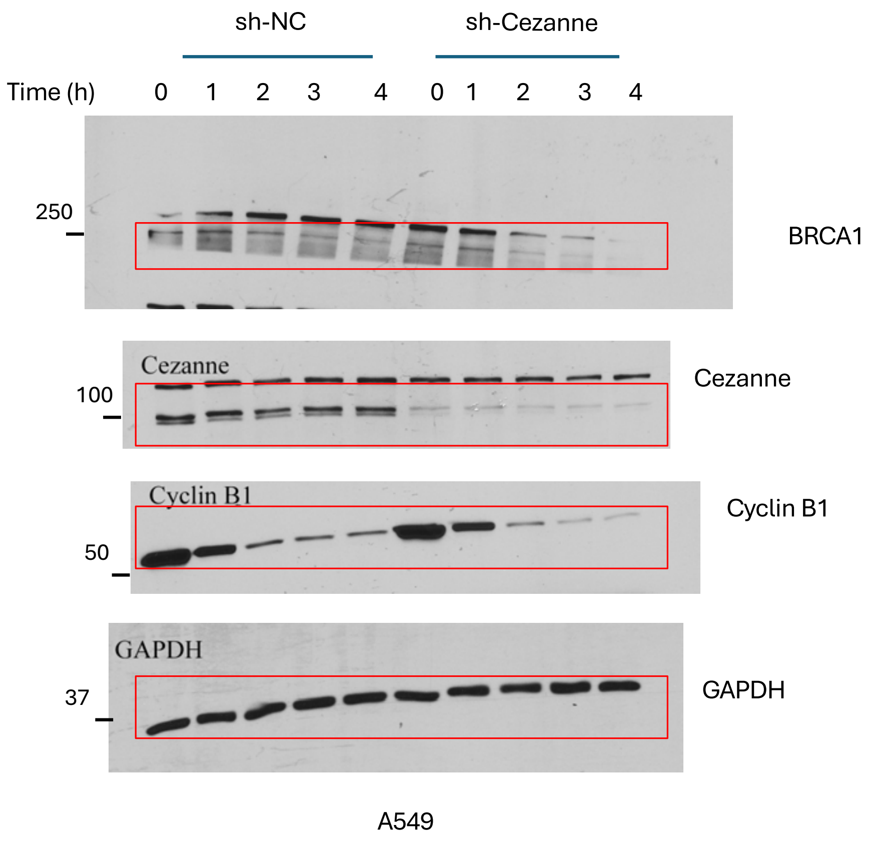

sh-NC
sh-Cezanne
Time (h)
0
1
2
3
4
0
1
2
3
4
250
BRCA1
Cezanne
100
Cezanne
Cyclin B1
50
Cyclin B1
GAPDH
37
GAPDH
A549

Fig S4F

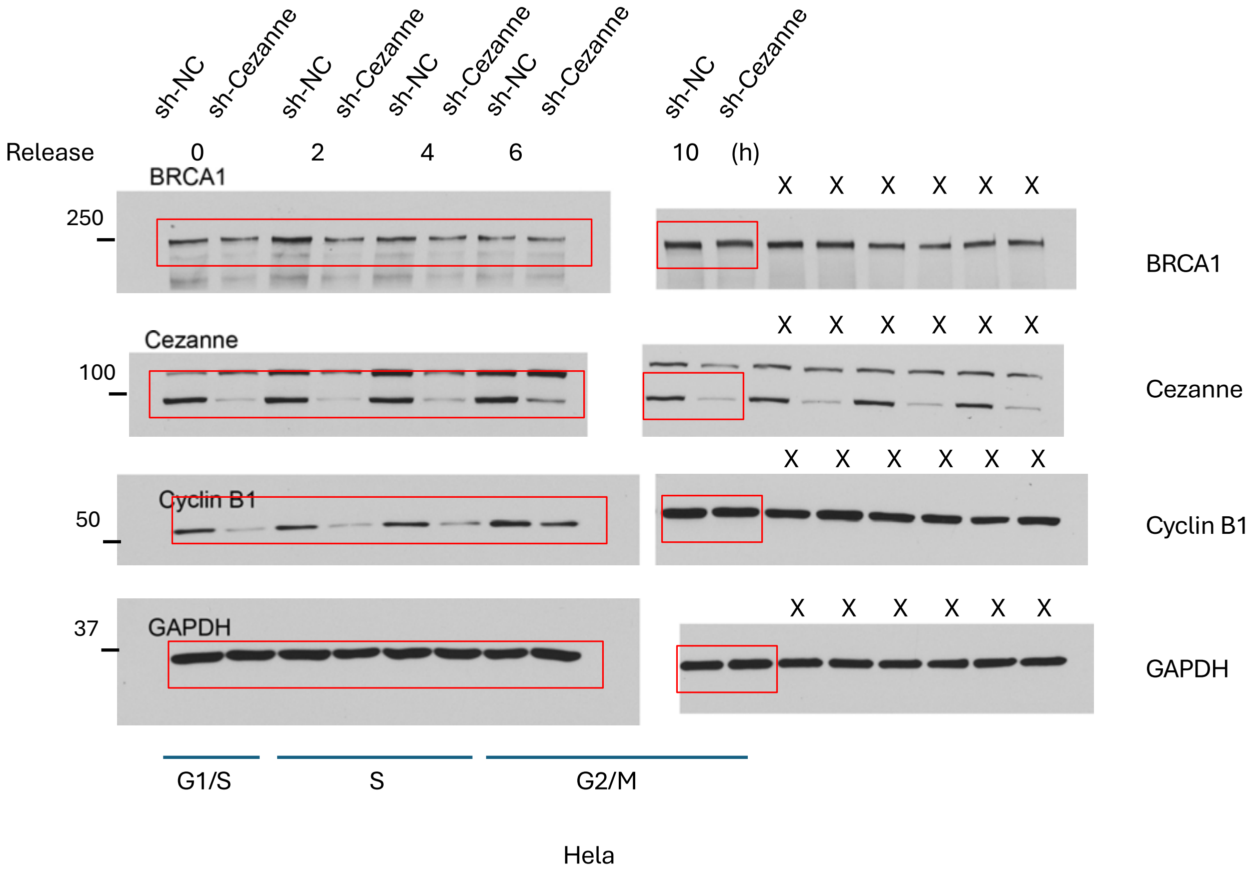
sh-NC
sh-Cezanne
sh-NC
sh-Cezanne
sh-NC
sh-Cezanne
sh-NC
sh-Cezanne
sh-NC
sh-Cezanne
Release
0
2
4
6
10
(h)
BRCA1
250
X X X X X X
BRCA1
Cezanne
100
X X X X X X
Cezanne
Cyclin B1
50
X X X X X X
Cyclin B1
GAPDH
37
X X X X X X
GAPDH
G1/S
S
G2/M
Hela

Fig S5C

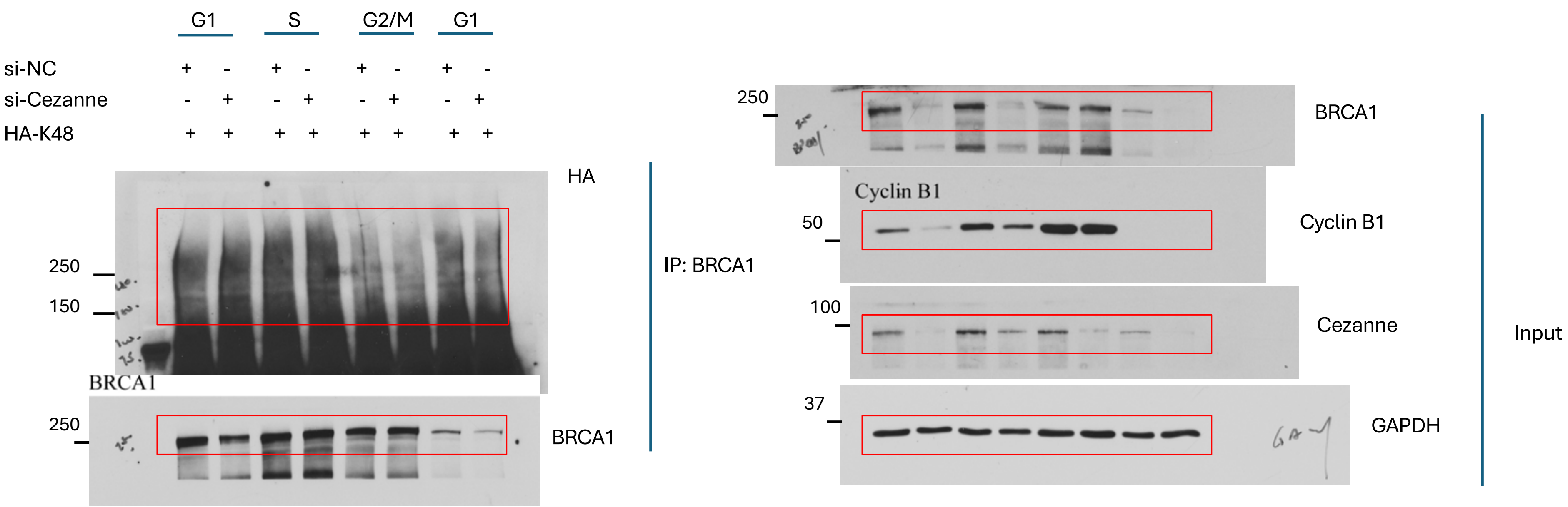

A549 cells expressing HA-K48 Ub

Fig S7A

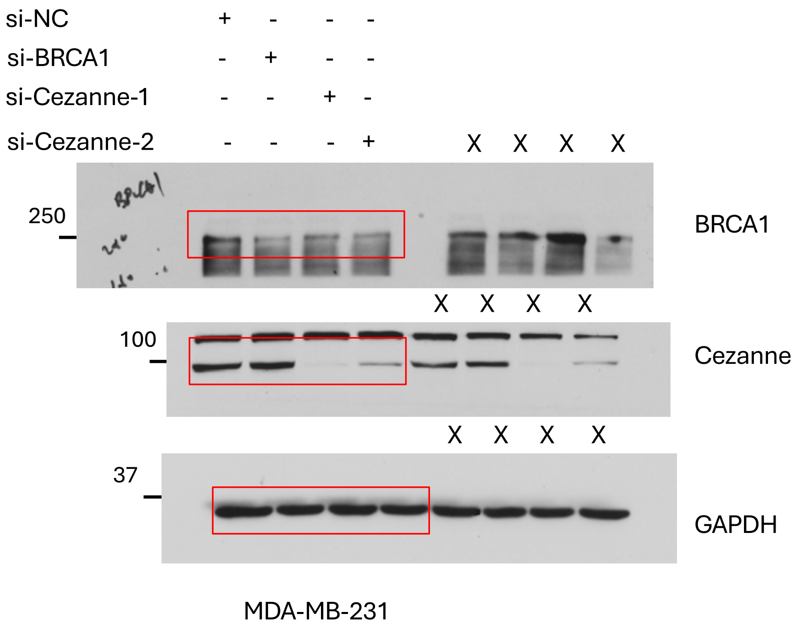

si-NC + - - -
si-BRCA1 - + - -
si-Cezanne-1 - - + -
si-Cezanne-2 - - - +
X X X X
250
BRCA1
X X X X
100
Cezanne
X X X X
37
GAPDH
MDA-MB-231

Fig S7B

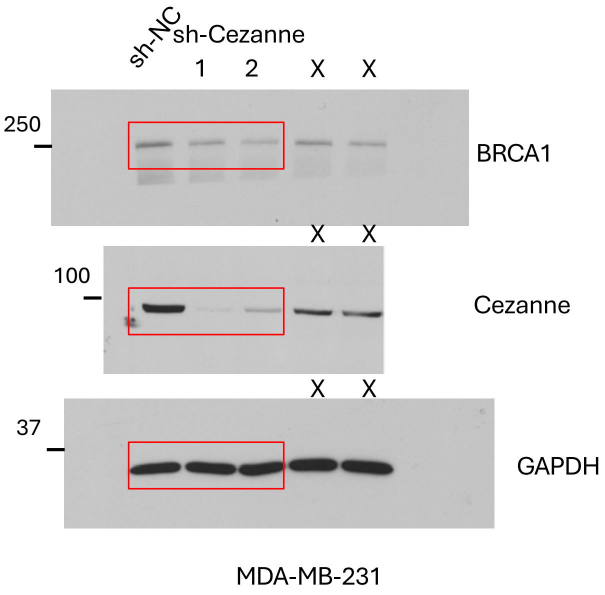
sh-NC
sh-Cezanne
1
2
X
X
250
BRCA1
X
X
100
Cezanne
X
X
37
GAPDH
MDA-MB-231

Fig S7E

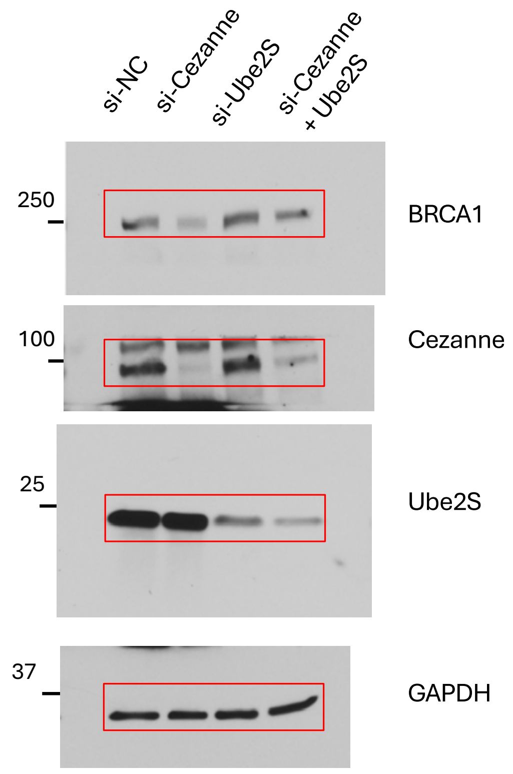

MDA-MB-231

Fig S7F

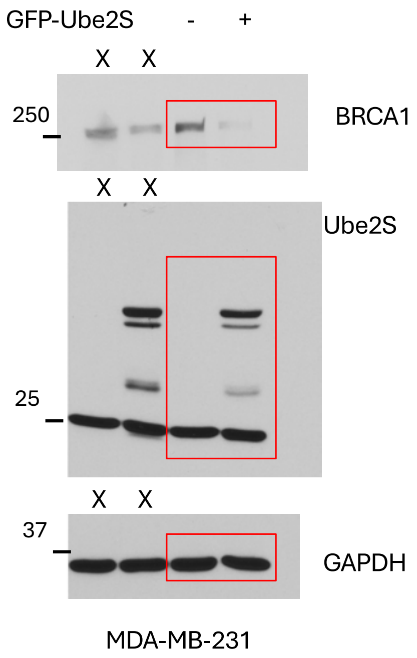

Supplement: S1 Raw Images — (PDF) [file pbio.3003545.s009.pdf]
